# Alkaline earth atom doping-induced changes in the electronic and magnetic properties of graphene: a density functional theory study†

**DOI:** 10.1039/d0ra08115a

**Published:** 2021-02-03

**Authors:** Ace Christian F. Serraon, Julie Anne D. Del Rosario, Po-Ya Abel Chuang, Meng Nan Chong, Yoshitada Morikawa, Allan Abraham B. Padama, Joey D. Ocon

**Affiliations:** Laboratory of Electrochemical Engineering, Department of Chemical Engineering, College of Engineering, University of the Philippines Diliman Quezon City 1101 Philippines afserraon@up.edu.ph jdocon@up.edu.ph +63 981 8500 loc. 3213; Thermal and Electrochemical Energy Laboratory, School of Engineering, University of California Merced CA 95343 USA; School of Engineering, Chemical Engineering Discipline, Monash University Malaysia Bandar Sunway Selangor Darul Ehsan 47500 Malaysia; Department of Precision Engineering, Graduate School of Engineering, Osaka University Suita Osaka 565-0871 Japan; Institute of Mathematical Sciences and Physics, College of Arts and Sciences, University of the Philippines Los Baños Laguna 4031 Philippines abpadama@up.edu.ph

## Abstract

Density functional theory was used to investigate the effects of doping alkaline earth metal atoms (beryllium, magnesium, calcium and strontium) on graphene. Electron transfer from the dopant atom to the graphene substrate was observed and was further probed by a combined electron localization function/non-covalent interaction (ELF/NCI) approach. This approach demonstrates that predominantly ionic bonding occurs between the alkaline earth dopants and the substrate, with beryllium doping having a variant characteristic as a consequence of electronegativity equalization attributed to its lower atomic number relative to carbon. The ionic bonding induces spin-polarized electronic structures and lower workfunctions for Mg-, Ca-, and Sr-doped graphene systems as compared to the pristine graphene. However, due to its variant bonding characteristic, Be-doped graphene exhibits non-spin-polarized p-type semiconductor behavior, which is consistent with previous works, and an increase in workfunction relative to pristine graphene. Dirac half-metal-like behavior was predicted for magnesium doped graphene while calcium doped and strontium doped graphene were predicted to have bipolar magnetic semiconductor behavior. These changes in the electronic and magnetic properties of alkaline earth doped graphene may be of importance for spintronic and other electronic device applications.

## Abstract 2

Alkaline earth atom dopants on graphene induce work function tuning and spin polarized electronic properties by ionic bonding.

## Graphical abstract

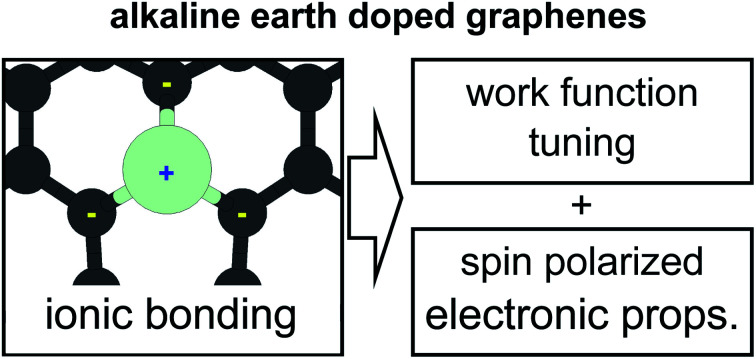

## 1. Introduction

Theoretical research for alkaline earth dopants in graphene has had a relatively recent history beginning with studies on beryllium doping in graphenes.^1^ This and succeeding studies^2^ showed that Be-graphenes have indirect p-type semiconducting behavior. This implies that the substitutional doping of Be atoms on graphene induce a band gap opening on the material, which is attributed to electron localization due to electron transfer from the Be atom to the graphene substrate.

Be-graphene was further studied for tunability with concentration effects and co-doping with a view towards use as potential anodes for lithium-ion^3^ and sodium-ion batteries.^4^ In particular, further theoretical development on beryllium–nitrogen co-doped graphene is being pursued in order to determine the feasibility of this material for metal-ion battery chemistries.^5^ In addition, the adsorption and migration behavior of other alkaline earth metals (particularly Mg and Ca) on pristine and defective graphene have been examined for divalent metal ion battery chemistries.^6^

The application of alkaline earth doped graphenes in hydrogen adsorption for H_2_ storage applications were also explored *via* theoretical studies.^7–9^ Doping alkaline earth graphene on divacancy sites^9^ or on top of the pristine graphene substrate^8^ were found to induce hydrogen adsorption. For graphene oxides, oxygenous sites on the material were found to be areas where magnesium dopant are likely attached. It was also found that this interaction induces polarization on H_2_, making adsorption more favorable.^7^

Co-doping beryllium with sulfur^10^ and with nitrogen,^11^ were also investigated for band-gap tuning. Optical properties, such as the optical transparency, for the Be–N co-doped graphene were found to be tunable *via* this co-doping approach.^12^ A similar study on the co-doping of magnesium and nitrogen was also conducted by Rafique and colleagues^13^ where magnetic coupling was found as the main mode of interaction and half-metallic properties were observed. Similar studies on doping AE-atoms on graphene have also been done on boron nitride, a two-dimensional material similar to graphene.^14^

Theoretical studies on NH_3_, PH_3_ and AsH_3_ molecule adsorption on alkaline earth doped graphenes for sensing applications have been conducted^15^ using dispersion corrected density functional theory as implemented in Dmol3 (ref. 16) with the double numerical polarization basis set. It was found that the alkaline earth dopants enhance the chemisorption of these molecules. Selectivity for the various compounds considered depends on the dopant. This may also indicate that alkaline earth doped graphenes can increase the chemical reactivity of graphene-based materials.

Recently, experimental studies with synthesized graphene doped with alkaline earth elements have been done. In particular, Ca-doped graphene has been synthesized *via* chemical vapor deposition for possible application in optoelectronics and organic liquid emitting diodes.^17^ Doping calcium on graphene was found to have reduced the work function of the material, implying an n-type doping behavior. Ca clusters of around 30 nm was also observed and the doped graphene material was utilized to fabricate an organic field effect transistor and observations were done in an N_2_ atmosphere.

Magnesium doped carbon quantum dots in the range of 2–8 nanometers in diameter, on the other hand, were synthesized *via* a procedure consisting of mechanical ball milling, ultrasonication and freeze-drying of a mixture of cellulose and Mg powder.^18^ These carbon quantum dots were found to be able to selectively detect iron (Fe^3+^) ions *via* changes in observed bright blue (*λ* ≈ 456 nm) photoluminescence upon exposure to ultraviolet light. This detection was attributed to metal chelation on the carbon quantum dots. This may be causing an electron transfer between Fe^3+^ and the Mg-CQD, extinguishing the Mg-CQD's photoluminescence. Detection levels of around 50 ppb for Fe^3+^ were obtained.

These recent studies are indicative of the interest in using alkaline earth group of elements as dopants for various applications. This study hence also intends to contribute to this body of knowledge by theoretically determining the properties induced by doping. While previous theoretical studies have attempted to screen alkaline earth dopants for electronic, optical and magnetic properties,^19,20^ these studies sometimes give conflicting results. For instance, while the study by Rafique and their team^19^ predict for the emergence of magnetic properties for some alkaline-earth doped graphenes and postulated ionic bonding as the source of magnetic properties in alkaline earth doped graphenes, Arsent'ev and colleagues^20^ do not predict magnetic properties for any alkaline earth dopant and was silent on the possible chemical interactions. Thus, the electronic properties of the alkaline earth doped graphene can still be described and understood better through elucidating the nature of chemical interactions within these materials. As a further example, while previous work on Be-graphenes^1,4^ indicate a band gap opening, other work indicates possible half-metallic behavior.^19^ Thus, it may also be possible that the differing computational parameters and details play a role in the discrepancies between the previous theoretical works.

In addition, the current experimental studies, while alluding to possible electronic structure-derived properties such as the presence of a band-gap (as in the case of photoluminescence in Mg-GQD^18^) or the nature of charge carriers in the material (as in the case of n-type doping in Ca-graphene^17^), did not directly provide a description of the electronic properties of the material by experimental methods. Further, the experimentally realized atomic-scale structure of these materials have not yet fully examined in detail. Also, while theoretical studies have been conducted on alkaline earth doped graphene, there is still a knowledge gap with respect to how more precisely the interactions induced by alkaline earth dopants on graphene can be described and how it gives rise to electronic and magnetic properties predicted for or observed on the material.

Thus, theoretical study is needed to provide a more thorough probing of the chemical interactions between the alkaline earth atoms and the graphene substrate to determine the modifications in the electronic and magnetic properties in these graphene based systems. This will allow for a more fundamental understanding of the patterns and a general description of the atomic interactions with a detailed analysis of the electronic properties. It is relevant to explain the observed properties by providing insights as to why these properties emerge and elucidate similarities and differences in each different dopant atom considered. Further, examining alkaline earth element dopants as a group in this manner will elucidate trends which can guide future experiments and development of the utilization of these materials in graphene.

## 2. Computational details

Alkaline earth doped graphene was modeled as a supercell of 4 × 4 unit cells of graphene, implying that each supercell contains 32 atoms. The supercell has one carbon atom substituted with an alkaline earth atom (*i.e.* beryllium, magnesium, calcium, and strontium). The larger supercell compared to previous work was chosen in order to minimize dopant–dopant interactions while still being computationally economical and to emphasize the dopant–substrate interaction. This difference in supercell size *versus* previous studies^19,20^ may have a significant effect especially when considering the comparability of the results presented in this work. It was also assumed that there is one adsorbate per supercell. The unit cell lattice constant was determined to be 2.470 Å, close to the accepted value of 2.46 Å for graphene. Thus, the 4 × 4 supercell has a lattice dimension of 9.88 Å. This was kept constant while all the atoms in the supercell were allowed to relax. A vacuum spacing of 15 Å was used to minimize spurious interlayer interactions introduced by the periodic boundary conditions assumed by the software.

Quantum ESPRESSO, a free and open source software suite, was used in order to implement first-principles numerical calculations *via ab initio* spin-polarized density functional theory.^21,22^ Pseudopotentials^23^ using projector augmented wave (PAW) were used in order to describe electron–ion interactions for all atomic species in the system. The Perdew, Burke and Enzerhof (PBE)^24^ formulation for the generalized gradient approximation (GGA) was used to account for the energy contributions resulting from quantum effects and electron–electron interaction *via* exchange correlation. The PAW-PBE pseudopotentials used were those provided by PSLibrary.^25^ Kinetic energy cutoffs was determined by convergence testing to be 550 eV for the wave function and 5500 eV for the charge density. Results at higher cutoff (1200 eV for the wavefunction and 12 000 eV for the charge density, respectively) agree with those obtained at lower cutoff (550 eV) and thus the results are also verified at that higher cutoff (Fig. H1–H21 and Tables H1 and H2†). The relatively high cutoff energy used for verification for this study was necessitated by the explicit treatment of the semicore states (1s orbital) for the Be pseudopotential in PSLibrary. Convergence criteria for energy in the self consistent calculations was set to be 1.36 × 10^−5^ eV.

Hellman–Feynman forces were minimized to below a threshold of 0.01 eV Å^−1^*via* geometric relaxation. All atoms were unrestricted for the geometric relaxation. A 4 × 4 × 1 *Γ*-centered Monkhorst–Pack^26^ grid sampling of the Brillouin zone, along with a Gaussian smearing of 0.14 eV. A denser *k*-point grid of 24 × 24 × 1 *k* points was used in order to determine density of states. Dipole correction^27^ was included to further minimize errors due to induced interlayer interactions by periodic images across the vacuum space. Convergence test results can be found in Fig. S1.†

VESTA,^28^ a 3D visualization program for volumetric data in crystals, and XCrysDen^29^ was used to generate figures for the electron density and other pertinent qualitative parameters derived from electron or charge densities in the systems considered.

The results of the DFT calculations were then analyzed to determine the properties derived from the spatial electron density distribution and to predict the physical and chemical properties of the materials. Binding and adsorption energies were used to quantify the stability of the materials modeled in DFT. These quantities were computed using formulas described below:1
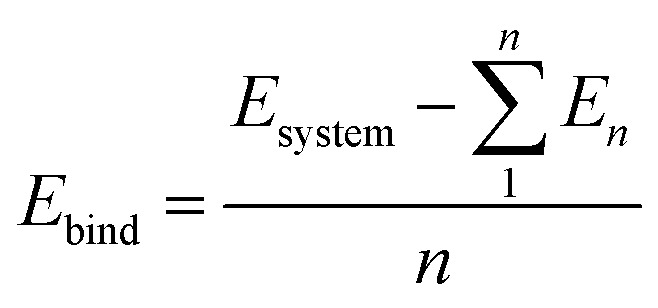

2 *E*_ads_ = *E*_system_ − (*E*_adsorbate/dopant_ + *E*_substrate_)

where *n* is the number of atoms constituting the system and where total energies are used for the computation. The adsorption energy essentially models the energy change when an alkaline-earth dopant attaches itself to the vacancy in a graphene sheet with a monovacancy, without considering the energy required to take the alkaline earth dopant from its source. Binding energy on the other hand refers to the energy required to build the system from its constituent atoms. Both formulations for energies were used in order to facilitate comparison with previous literature.

Bader charge analysis,^30^ as implemented by Tang, Sanville and Henkelman^31,32^ was used in order to determine the charges on the constituent atoms and to determine if electron transfer and charge concentrations can be observed in the system. Bader charge analysis allows for an unambiguous partition of electrons to nuclei or atoms making up a chemical system based on a separation in space of the electron density where interatomic separation surfaces are defined as where the flux of the gradient of electron density approaches zero.^33^ This also provides a quantitative picture with regards to the electron density distribution over the material.

Two qualitative measures of interaction were examined to describe the chemical interactions in AE-graphenes. The electron localization function (ELF)^34,35^ and reduced density gradient (RDG)^36^ were used to qualitatively evaluate chemical interactions on the materials. The ELF characterizes the localization of electrons by determining the likelihood that an electron can be found near a reference electron of the same spin and at a given point. Thus, ELF^37^ can be used in order to find areas where electrons tend to be located or localized. This is an important qualitative indicator for bonding behavior, with covalent and ionic bonding being differentiated by where electron localization occurs.^38^

On the other hand, non-covalent interactions tend to be observed at troughs or regions of lowered electron density.^36,39^ Hence, the RDG would be necessary in order to evaluate these interactions which are associated with these low electron density areas. This means that the RDG can be used to account for non-covalent (that is not characterized solely by electron localization) interactions, such electrostatic interactions and steric hindrances on the material.

This coupled approach using the ELF and the RDG has already been used in previous work to characterize complex organic chemical systems^40^ since it allows for a visualization of interactions at both high electron density zones (typified by shared electron interactions such as bonding as visualized *via* the ELF) and low electron density regions (such as steric hindrances as visualized through the RDG).^41,42^ This approach thus enables simultaneous and rigorous qualitative examination of the different interactions within a material.

Density of states (DOS) and band structures were also derived from the DFT calculations in order to further characterize the electronic and magnetic properties observed in the modeled materials. The projected density of states (pDOS) was also used in order to determine hybridization by determining the probability that an electron of a particular orbital and atom occupies a certain energy level^43^ and thus provide another picture into the nature of bonding *via* orbital hybridization within the substance.

## 3. Results and discussion

### 3.1. Chemical interactions in AE-graphenes

Binding and adsorption energies were used to evaluate the energetic feasibility of different alkaline earth dopants in graphene. Monosubstituted alkaline earth dopants were generally found to favor a non-planar doping configuration, with the alkaline earth dopant sticking out of the graphene substrate. This is consistent with our previous work^44^ even when using the 0.4 eV criteria set by Ishii *et al.*^20,45^ This configuration will be referred to as out-of-plane doping. Table 1 shows the binding energy and adsorption energy trends for AE-graphenes. Negative adsorption and formation energies, which imply thermodynamic feasibility were observed. Some buckling was also observed on the graphene sheet due to the doping.

**Table 1:** Energetics, bond lengths and net charges in AE-graphenes. Results calculated in this work are in **bold**. Further information on work function determination are found in Fig. S6. Results at 1200 eV cutoff at Table H1

System	*E*_bind_ (eV per atom)	*E*_ads_ (eV)	*W*_0_ (eV)	AE⋯C distance (Å)	Dopant net charge (*e*^−^)
**Be-graphene (out-of-plane)**	**−8.699**	**−6.506**	**4.806**	**1.623**	**+1.612**
Be-graphene^1^ a (out-of-plane)	−7.715	—	—	1.62	+2.0
Be-graphene^44^ a^,^^c^ (out-of-plane)	−8.699	—	—	1.622	+2.0
Be-graphene^15^ a (out-of-plane)	—	−7.02	—	1.48	−0.793
Be-graphene^2^ a (in-plane)	−8.86	—	—	1.56	+2.0
Be-graphene^44^ a^,^^c^ (in-plane)	−8.692	—	—	1.568	+2.0
**Mg-graphene**	**−8.553**	**−1.855**	**3.136**	**2.080**	**+1.366**
Mg-graphene^15^ a	—	−2.10	—	2.11	+0.751
**Ca-graphene**	**−8.559**	**−2.057**	**2.053**	**2.234**	**+1.315**
Ca-graphene^15^ a	—	−3.59	—	2.30	+1.499
Ca-graphene^17^ b	—	—	3.60	—	—
**Sr-graphene**	**−8.592**	**−3.072**	**1.815**	**2.449**	**+1.355**
Sr-graphene^15^ a	—	−4.19	—	2.60, 2.48^e^	+1.125
**Graphene**	**−9.029**	**−17.051**	**4.265**	**1.426**	**—**
Graphene^17,46–54^ b^,^^d^	—	—	4.2–4.8	1.44	—
Graphene^55–63^ a^,^^d^	—	—	4.23–4.66	1.42–1.44	—
Graphene^15^ a	—	—	—	1.42	—
**Graphene with monovacancy**	**−8.497**	**—**	**—**	**—**	**—**

a Theoretical result.

b Experimental result.

c Authors' previous work.

d Range provided from results by previous work.

e Two values reported since asymmetrical adsorption was predicted.

AE–C distances was found to increase as the heavier atom is doped, with Be-graphene having the shortest distance and increasing as the atomic number of the dopant atom increases. These bond lengths were found to be longer than the C–C bond length found in pristine graphene. This may be a factor which induces an out-of-plane doping for Be-graphenes. Further, comparing results for Be-graphene with those determined from previous DFT calculations show similar results in terms of AE–C distances predicted. Slight variation in predicted binding energies is observed between the results in this study and previous work^1,2,15^ which may be attributed to differences in computational details and particularly supercell size.

The doping of Be atom on graphene was found to be most stable and lowest in energy. While this stability may be attributed to the low atomic weight (and therefore low atomic radius) of Be atoms as demonstrated by the lowest AE–C distance it shows, it does not explain why Mg was found to be the highest energy (and therefore least stable) dopant atom. This is despite Mg-graphene having the second shortest AE–C bond length. This may imply that factors other than the atomic weight may play a role in determining energetic stability on AE-graphene doping.

Comparing the adsorption energy of the alkaline earth dopants with that of adsorbing a carbon atom into the monovacancy shows that the adsorption of carbon into the monovacancy is still more energetically favorable than substituting alkaline earth dopants, or that carbon atoms are preferentially adsorbed in point vacancies over alkaline earth metal atoms. This is consistent with the previously-reported self-healing of defects or self-knitting behavior in graphene.^65,66^

Be-graphene was found to have a higher work function (*W*_0_) compared to pristine graphene, while the rest of the alkaline earth dopants lower the work function relative to pristine graphene. The heavier alkaline earth atoms were also found to have stronger work function reduction as the atomic weight increases and they have a electrostatic potential barrier attributed to the dopant (Fig. S6†). It must be noted that PAW-PBE tends to underestimate work functions,^67^ as was demonstrated with the work function prediction for pristine graphene and previous studies, but the trend of work function increase or decrease is expected to hold.^17,56,68^ Further, the experimentally measured work function, as well as theoretically predicted work function of graphene also falls within a range of values, depending on the particular measurement and synthesis details,^54,59^ hence the predicted trend may be more useful to point towards the feasibility to use the other alkaline earth dopants to tune the work function of graphene. Theoretically^63^ and experimentally,^64^ it was determined that the observed work function of graphene may also be dependent on the thickness or number of layers observed.

Results for Ca-graphene have a consistent trend with results by Klain *et al.*^17^ where they also noted that calcium doping on graphene caused a work function lowering compared to pristine graphene. However, this work predicted a higher work function lowering (2.2 eV) due to calcium doping than the 1 eV work function lowering measured in their work. Ca clusters of around 30 nm in size on the graphene surface was observed *via* atomic force microscopy in their work and a relatively high concentration of Ca atoms was measured for their synthesized doped graphene. On the other hand, this work focused on atomic doping of calcium in graphene. This is an important difference between this work and their work and this may explain the discrepancy in the predicted *versus* observed work function in the experiment, especially considering there are more complex structures realized in experiment.

Further, the atomic-level structures were not observed directly for the synthesized samples. However, strong chemical bonding between C and Ca, which was inferred in the experimentally realized synthesized doped graphene through X-ray photoemission spectra. Despite this, this work may help elucidate the contribution on the work function of strong chemical bonding between C and Ca, especially considering that the experimental result does not preclude this possibility. Therefore, it may also be important to investigate further the effect of clusters of alkaline earth elements on workfunction, as well as to further investigate the atomic-level structures in experimentally realized AE-graphene.

These work function shifts predicted may be caused by the electron transfer from the dopant to the material. Electron transfer from the graphene to the dopant is expected to increase the work function,^69^ while electron transfer from the dopant to graphene is expected to decrease the work function.^17^ The beryllium atom has, overall, fewer electrons associated with it than carbon. Thus, it is expected to gain electrons from the carbon atoms in graphene, following chemical potential equalization or electronegativity equalization.^70,71^ This is despite the tendency for the beryllium atom to transfer electrons to empty its 2s orbital. This effect of the electronegativity equalization is an important consideration to take note of for the interactions on Be-graphene. Conversely, the heavier alkaline earth dopants have absolutely more electrons than carbon even upon the transfer of their outermost s-orbital atoms and thus will tend to donate electrons to graphene.

**Fig. 1:**
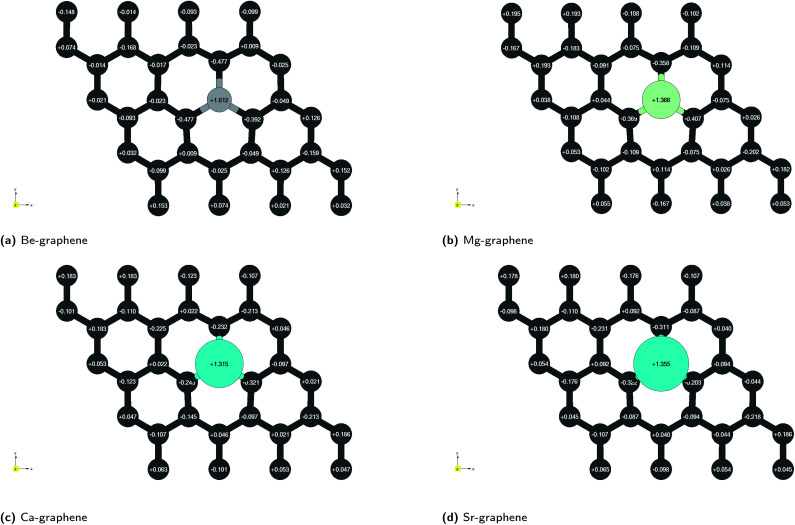
Bader charge analysis for AE-graphenes: (a) Be-graphene, (b) Mg-graphene, (c) Ca-graphene and (d) Sr-graphene. Net charges for each atom from Bader charge analysis are overlaid on the figure. Grey indicates carbon atoms while the differently colored atom indicates the alkaline earth dopant. Structures for AE-graphenes are detailed in Fig. S2–S5.† Results at higher cutoff (1200 eV) available at Fig. H1.†

This electron transfer is further demonstrated by localized regions of positive and negative charge that emerge from the doping of alkaline earth dopants on graphene, as quantified with Bader charge analysis (Fig. 1). This may indicate possible charge transfer of an electron pair from one atom to another, since positive and negative charges greater than 1*e*^−^ have been observed.

**Fig. 2:**
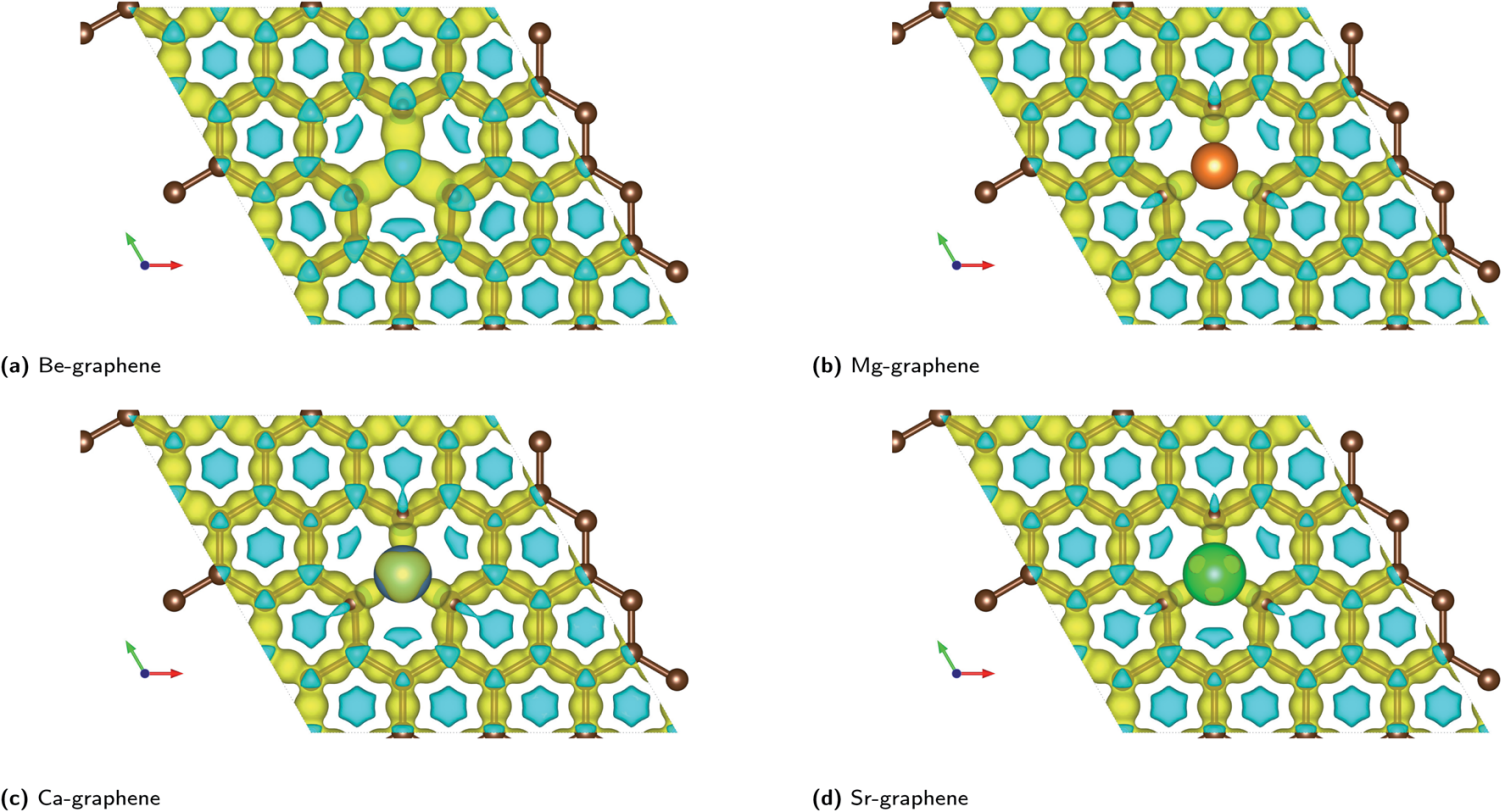
Charge difference plots for alkaline-earth doped graphenes: (a) Be-graphene, (b) Mg-graphene, (c) Ca-graphene and (d) Sr-graphene. Yellow indicates regions which have an increase in electrons (negatively charged areas) while cyan indicates regions which have a decrease in electrons (positively charged areas). Isosurface level = 0.013*e*^−^ per Bohr^3^. Brown atoms are carbon atoms while the differently colored atom denotes the alkaline earth dopant. ESI at Fig. S7.† Results at higher cutoff (1200 eV) available at Fig. H7.†

In this case, the most salient localized modification in AE-graphenes is the electron deficient, and hence positive charge-dominated region on the AE-dopant atom. These are paired to the negative-charge dominated region on the carbon atoms adjacent to the substrate, as demonstrated in charge difference plots in Fig. 2. This positively charged site in turn is neighbored by negatively charged carbon atoms.

It must be noted that for Be-graphene, Luo and colleagues^15^ predicted a negative dopant net charge using Mulliken charge analysis. The trends they predicted for the dopant net charge are also different with the predictions for this work. In contrast, this study, as well as other previous studies^1,2^ which used Bader charge analysis, predict for a positive charge for the beryllium atom. Thus, this discrepancy may be attributed to this difference in methodology. Further, it had been previously noted that results from Mulliken charge analysis depend strongly on the basis set used,^3,72^ with smaller basis sets tending to have unrealistic results.

**Fig. 3:**
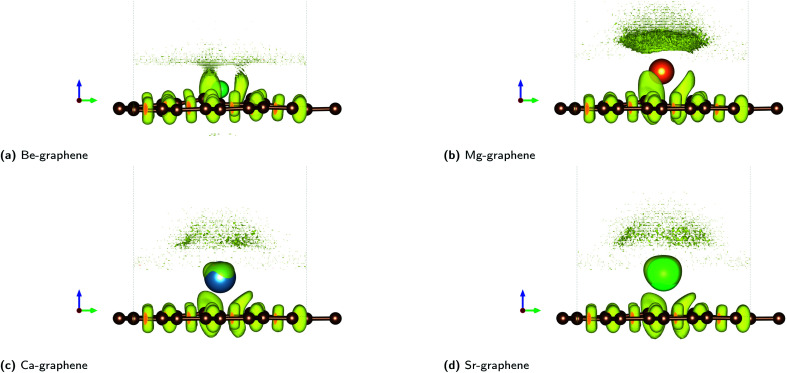
Electron localization function (ELF) plots for AE-graphenes: (a) Be-graphene, (b) Mg-graphene, (c) Ca-graphene and (d) Sr-graphene. Brown atoms are carbon atoms while the differently colored atom denotes the alkaline earth dopant. Isosurface level = 0.8 based on recommendation by Savin *et al.*^35^ ESI at Fig. S8.†

The electron localization functions for AE-graphenes (Fig. 3) indicate that there are regions of electron localization towards the carbon atoms adjacent to the alkaline earth dopant. These are in the form of lobes which point toward but do not touch the alkaline-earth dopant. This is similar to the observations in Fig. 2. This implies ionic bonding since there is no localization in between the two atoms, which would have been characteristic of a coordinate covalent bond. For the C–C bonds, the ELF tends toward the middle of the two atoms, implying electron sharing or predominantly covalent bonding.

It is also interesting to note that for Ca- and Sr-atoms, the shape of the ELF on the dopant atoms are distorted away from a spherical form. This may imply that the electrons on the Ca- and Sr-dopant, respectively, are polarized. This is in consonance with the observation for the respective charge difference plots (Fig. 2c and d). It must be carefully noted that regions of low electron density tend to cause numerical artifacts or noise on ELF due to rapidly varying ELF *versus* electron density.^73^ This is most apparent near the alkaline-earth dopants, which exhibit high positive charge and therefore, low electron density.

**Fig. 4:**
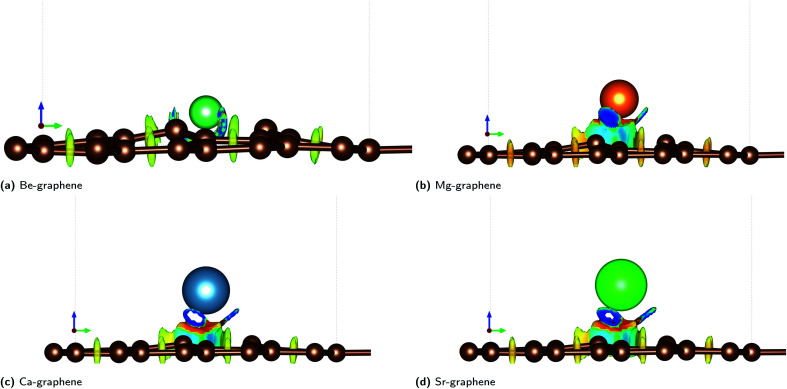
Reduced density gradient (RDG) plots for AE-graphenes: (a) Be-graphene, (b) Mg-graphene, (c) Ca-graphene and (d) Sr-graphene. Spectrum from red to blue indicates repulsive to attractive interactions, respectively. Brown atoms are carbon atoms while the differently colored atom denotes the alkaline earth dopant. Isosurface level = 0.4. ESI available at Fig. S9.† Results at higher cutoff (1200 eV) available at Fig. H11.†

Non-covalent interactions (NCI) were also found to play a significant role in AE-graphenes as demonstrated by the reduced density gradient.^36^ Both repulsive and attractive interactions were demonstrated to be present in AE-graphenes (Fig. 4). In particular, blue regions were generally found between the alkaline earth dopant and the graphene substrate with the notable exception of Be-graphene. This is further indicative of ionic interactions between the alkaline earth dopant and graphene substrate since strongly attractive interactions indicated by the RDG may imply electrostatic attraction. For Be-graphenes (Fig. 3a), a blue region between the dopant atom and the adjacent carbon atom is absent. This may imply that those regions have a low RDG and thus may imply that there is a differing or variant bonding characteristic.

Red regions, on the other hand, were observed at the bottom of the alkaline-earth dopant. This may indicate steric hindrances or repulsive interactions. This also serves to explain the out-of-plane doping observed for AE-graphenes. This is more pronounced for Ca- and Sr-graphenes due to its larger atomic number. It can also be observed that there are voids or holes in the middle of blue regions for Ca- and Sr-graphenes. It may imply that there is a high enough electron density in that region for the NCI to not be visible given the isosurface level, since the NCI tends to zero in regions of high electron density.

The complementary pictures given by the ELF and the RDG qualify the nature of interaction within AE-graphenes. Ionic bonding or electron transfer from the alkaline earth dopant to the graphene substrate was found to be the main mode of interaction for AE-graphenes. The three nearest neighbor carbon atoms primarily receive and share the electrons transferred to the graphene substrate between them. In turn, steric hindrance is caused by the graphene substrate which competes with the ionic bonding and causes out-of-plane doping on AE-graphenes.

The variation for Be-graphene relative to the other alkaline earth doped graphenes may be attributed to its lower atomic number relative to carbon. Once the beryllium atom transfers its valence electrons to the graphene substrate, it would still have fewer electrons than carbon. This can attract the nearby electron cloud towards it to equalize the chemical potential of all electrons over the material. This may make the interaction appear more covalent since the electron cloud due to the electron transfer from the beryllium dopant is shifted away from the electron accepting carbon atom and pulled back towards the beryllium donor, thereby making the electron cloud less localized on the electron acceptor sites. Thus, the action of electronegativity equalization competes with electron transfer in the ionic bonding observed for Be-graphene. This competition may also explain the work function increase predicted for beryllium doping on graphene, which tends to be a characteristic of electron transfer from a graphene substrate to a dopant atom.^69^ The possibility for covalent bonding to occur on beryllium doping on graphene has also been previously documented.^19^ This is also consistent with beryllium being expected to have the most similar electronegativity to carbon among all alkaline earth elements.

In contrast, for magnesium and the heavier alkaline earth atoms, the core electrons would screen the effect of the positive nucleus and repel the electrons from the alkaline earth atom once it has been transferred to the graphene substrate. For instance, the magnesium ion would have a similar electron configuration to neon upon transferring two electrons. This still has more electrons than the carbon atom due to its higher atomic number. The core electrons remaining on magnesium would repel the nearby electron cloud and thus make the bond more localized on the electron acceptor sites. For the heavier alkaline earth atoms, this effect would be more pronounced since they have more core electrons.

Therefore, the action caused by electronegativity equalization would reinforce the effect of electron transfer in ionic bonding. This is also evidenced by the existence of regions of low electron density, and hence high RDG between the dopant atom and the adjacent carbon atom; a feature which is absent in Be-graphene. Another effect caused by electronegativity equalization is the potential barrier predicted near the heavier alkaline earth dopant atoms (Fig. S6b–S6d†), implying that electron repulsion can be induced on that site.

Ionic bonding, *i.e.* the key interaction governing the formation of the AE-graphene, can also be conceived of as comprising two distinct steps: (1) the ionization of the alkaline metal dopant to a cation by loss of valence electrons and (2) the reception of the valence electrons by the graphene substrate, binding the alkaline metal dopant to the graphene substrate. From the Bader charge analyses (Fig. 2), it can be observed that Be-graphene exhibits a 1.6*e*^−^ positive charge, while Mg-, Ca-, and Sr-graphene exhibit around 1.3*e*^−^ positive charge. This may indicate that the degree of electron transfer as measured by Bader charge analysis is greater for Be than for the rest of the alkaline earth atoms. It must be noted that this fractional value predicted does not physically imply that electrons are divisible, but rather is an artifact of the direct partitioning of the electron cloud as done using Bader charge analysis, which divides based on electron density gradients.

The electrons lost by the dopant atoms are primarily received by the carbon atoms adjacent to the alkaline earth dopant as demonstrated in charge difference plots in Fig. 2, which shows that the adjacent carbon atoms have similar negative charges, which means that the electrons transferred to these atoms evenly. The localization of this transfer is more prominent for the Be-graphenes and Mg-graphene. Some delocalization could be observed for Ca- and Sr-graphenes since the amount of electrons received by the adjacent carbon atoms is less than the total amount which was given up by the alkaline earth atom, implying that some of the electrons were received elsewhere on the graphene substrate. The amount of electrons transferred,^74^ in addition to the carbon-dopant distance (Table 1) should be indicative of the bond strength or ion-substrate coupling between the alkaline earth ion and the graphene substrate, with Be- and Mg-graphene, *i.e.*, the lighter alkaline earth elements expected to be more energetically stable over Ca- and Sr-graphene based on this criterion. However, the trend for binding and adsorption energy for the system indicates that while Be-graphene is indeed the most stable due to having the lowest binding and adsorption energies, Mg-graphene is actually least stable and is less stable than Ca- and Sr-graphene. This observation for the interaction of Mg and Ca atoms with graphene is also consistent with recent theoretical work comparing divalent metal migration on defective and pristine graphene surfaces,^6^ where calcium atoms were found to be trapped to a larger extent than Mg.

A similar trend and pattern for interaction was noted by Liu, Merinov and Goddard,^75^ with Mg exhibiting the weakest binding to various tested substrates among alkaline earth metals. This could be explained by taking note of ionization energy values for alkaline earth metal atoms, which show that the lighter elements have a higher ionization energy *versus* the heaver alkaline earth elements. Thus, it is expected that taking away electrons from the lighter alkaline earth elements requires more energy. On the other hand, ion-substrate coupling effects mostly account for electrostatic interactions between the alkaline earth ion and the substrate which has accepted the electron. This effect is primarily of an electrostatic nature. Hence, it is expected that smaller atoms or ions have lower (or more favorable) ion-substrate coupling because of weaker electrostatic repulsion or steric effect. Thus, from the perspective of ion-substrate coupling, coupling for Be ions is more energetically favorable than for Sr ions.

The competition between the strength of ion-substrate coupling, evinced by the degree of electron transfer as computed *via* Bader charge analysis and the differing ionization energies for the AE dopants, could probably explain the energetic trend observed.^75^ The strength of the ion-substrate coupling due to electron transfer is the dominant effect stabilizing Be-graphenes, while Ca- and Sr-graphenes are stabilized by the low ionization energy required to transfer their electrons to the graphene substrate. For Mg-graphene, the two effects compete with none being predominant, that is, the Mg atom transfers electrons to a lower degree but at the same time, this electron transfer requires a higher amount of energy. This causes the relatively more positive binding and adsorption energies for Mg-graphene. This trend may provide a guide for the application of graphene materials for battery technologies based on alkaline earth group atoms as divalent metal ions^6^ since a balance of adsorption strength is needed for reversible operation in ion batteries.

### 3.2. Spin-polarized electronic properties in AE-graphenes

An important consequence of the electron transfer and ionic bonding from the alkaline-earth dopant to the graphene substrate is spin-polarization in the electronic structure or the emergence of possible magnetization on AE-graphenes. Magnetization due to electron transfer has been previously observed and predicted for H-graphene^76^ and atomic cobalt doped graphene under tension.^77^ These works attribute the emergence of spin-asymmetry in doped graphene to the electron transfer to the graphene substrate. This may imply that inducing electron transfer or ionic interactions with graphene may render it magnetic, which could explain the emergent ferromagnetism in AE-graphenes. A similar case can be observed with AE-graphenes, particularly also for Rafique and team's work where they explain the interaction between the Mg and N co-dopants in graphene as a magnetic coupling.^13^

**Fig. 5:**
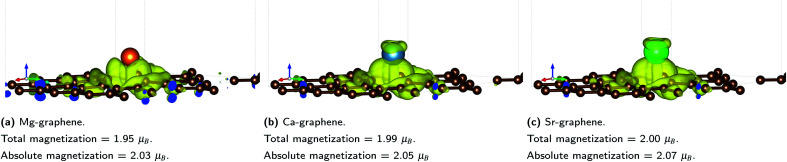
Spin-difference plots for AE-graphenes: (a) Mg-graphene, (b) Ca-graphene and (c) Sr-graphene. Yellow signifies majority-spin dominant regions (spin-up) while cyan signifies minority-spin dominant regions (spin-down). Isosurface level = 0.002*e*^−^ per Bohr^3^. Total and absolute magnetization values (*μ*_B_) for each AE-graphenes are also indicated. Spin-difference plots for Be-graphene not included since total and absolute magnetization for Be-graphenes is zero. Brown atoms are carbon atoms while the differently colored atom denotes the alkaline earth dopant. Results at higher cutoff (1200 eV) available at Fig. H13.†

Total and absolute magnetization values for AE-graphene demonstrate that AE doping on graphene tend to cause ferromagnetic properties on the material (Fig. 5). For all cases, the spin difference plots show that spin-polarization is mostly concentrated on carbon atoms in the vicinity of the dopant atom, implying localization of the spin-asymmetry. This is also where the ELF shows distinct localization on the carbon atoms nearest to the dopant (Fig. 3). This means that the magnetization may be attributed to the electrons transferred from the alkaline earth dopant to the graphene substrate in ionic bonding.

This spin-polarization on carbon-atoms may imply that Mg-, Ca- and Sr-graphenes provide examples for predicted p-orbital spin polarization.^78^ Similar p-orbital spin polarization (or magnetization) has also been predicted for alkaline-earth carbon compounds exhibiting a rock-salt structure,^79^ where these compounds were found to have half-metallic properties. Magnetization for these instances were primarily attributed to localization of p orbitals on the carbon component.

In contrast, previous studies have predicted that Be-graphene^1,2^ has no net magnetization. The emergence of magnetization (or the lack thereof), provides a further clue to differences in the exact manner by which the electron transfer occurs on AE-graphenes. For Be-graphenes, the competition between electronegativity equalization and electron transfer may have precluded spin-polarization, while for the other AE-graphenes, the heavily ionic interaction between the AE-dopant and the carbon atoms on the graphene substrate induce magnetization. The presence of this triplet-like ferromagnetic state, as indicated by the total and absolute magnetization (Fig. 5) in the other AE-graphenes may also serve to destabilize Mg-, Ca- and Sr-graphene relative to Be-graphene due to exchange effects. It must be noted that for Ca- and Sr-graphenes, some spin polarization is also observed on the alkaline earth atom dopant.

In general, band gaps were opened on the graphenes owing to the localization caused by s–p hybridization on the AE-graphenes. For Mg-graphene, a half-metallic Dirac cone was observed for the majority spin, along with n-type semiconductor behavior for the minority spin, while for Ca- and Sr-graphenes, ferromagnetic semiconductor behavior was observed. These parameters describing the predicted electronic structure of the materials are summarized in Table 2 and will be discussed in detail in the succeeding sections of this paper (Sec. 3.3, 3.4 and 3.5).

**Table 2:** Band structure information for AE-graphenes. VBM and CBM levels are relative to the material's Fermi level. VBM–CBM locations are indicated on the respective band structure plots (Fig. 7a, 8a and 9a). Results for higher (1200 eV) cutoff can be found at Table H2

Dopant	VBM (eV)	CBM (eV)	VBM–CBM gap (eV)	Gap type	VBM–CBM location
**Majority spin**					
Be	−0.159	0.122	0.280	Indirect, p-type	*Γ*–*M*
Mg	0.177	0.179	0.002	Direct, p-type	*K*–*K*
Ca	0.020	0.373	0.354	Direct, p-type	*K*–*K*
Sr	−0.055	0.306	0.360	Direct, p-type	*K*–*K*

**Minority spin**					
Be	−0.159	0.122	0.280	Indirect, p-type	*Γ*–*M*
Mg	−0.311	0.234	0.545	Indirect, n-type	*K*–*M*
Ca	−0.379	0.206	0.585	Indirect, n-type	*K*–*Λ*
Sr	−0.370	0.255	0.625	Indirect, n-type	*K*–*M*

It must also be explicitly indicated that these electronic and magnetic properties are different from those predicted by various previous theoretical works.^15,19,20^ This may be attributed to differences in supercell sizes used for the computation, with this study using a larger supercell size than the previous theoretical work. The supercell size may also be correlated to possible dependence on concentration or concentration effects, since there will be a differing ratio of carbon atoms on graphene to the dopant atom with an adjustment towards different supercell sizes.

Further, experimental studies^17,18^ vary when compared to the theoretical prediction in this work. For instance, Klain *et al.*^17^ posits n-type doping due the introduction of Ca atoms, while this work predicts simultaneous p- and n-type doping, albeit for different spins. These differences may be attributed to differences in the particular structures of experimentally synthesized doping in graphene. This may include larger features, such as clusters of alkaline earth atoms on graphene,^17^ disorder on the graphene substrate,^18^ size, edge and defect effects, as well as possible temperature effects since the experimental observations could be done at room temperature. Thus, further theoretical work may be done on systems which are more similar to actually observed doped graphene systems. Conversely, further experimental work may be done in order to better characterize and verify the structures and properties induced by doping alkaline earth metals in graphene as obtained *via* various synthesis and experimental methods or to provide experimental verification to the predicted properties by targeted synthesis to replicate these theoretically-predicted-for systems.

### 3.3. Beryllium-doped graphene: in consonance with previous studies

**Fig. 6:**
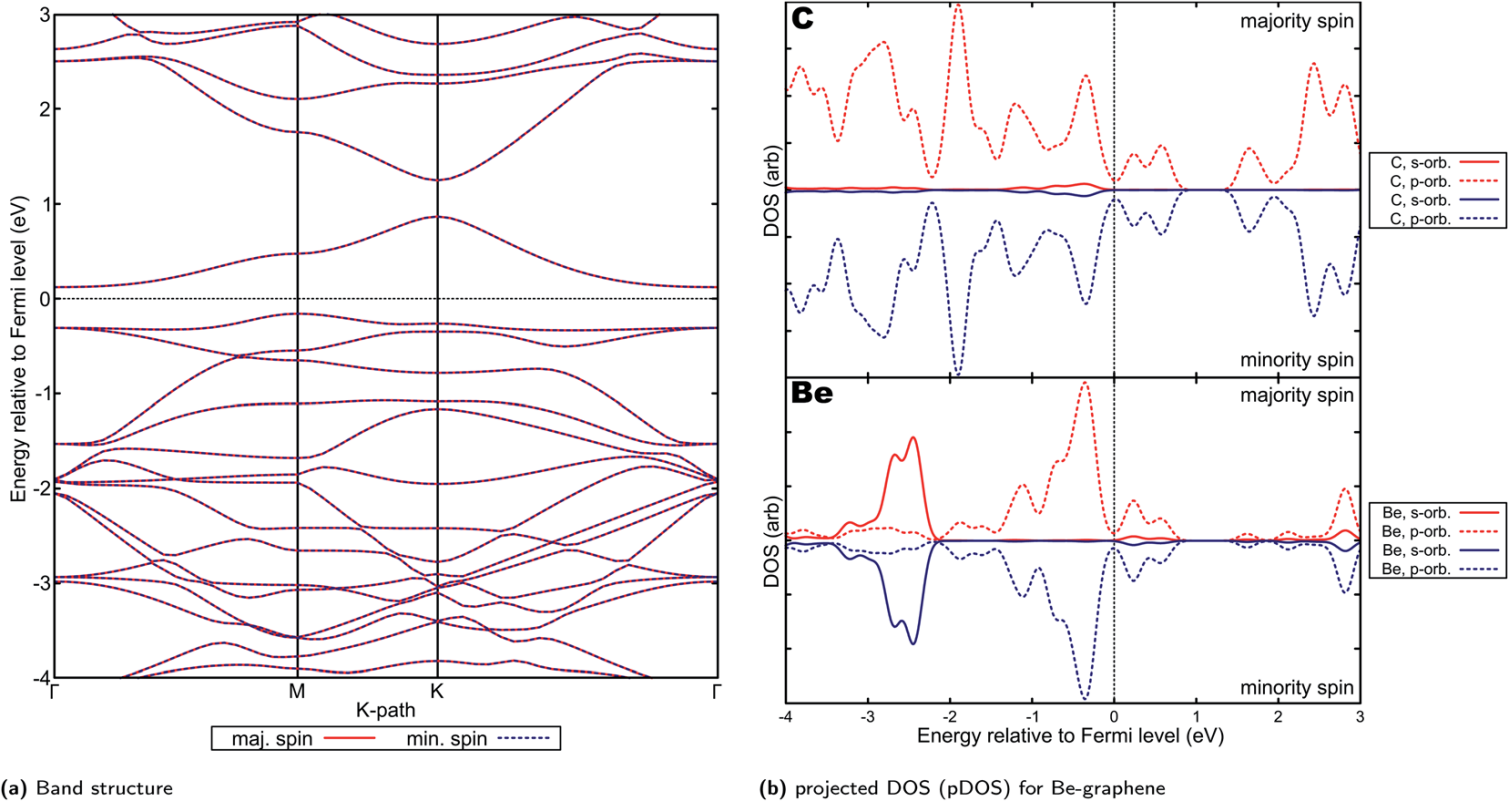
Electronic properties and orbital hybridization for Be-graphene: (a) band structure and (b) projected density of states (pDOS). Red indicates majority spin (spin up) states and blue indicates minority spin (spin down) states. ESI for electronic properties at Fig. S10.† Results at higher cutoff available at Fig. H14.†

Be-graphenes were found to have no spin asymmetry or magnetic properties (Fig. 6), also consistent with previous studies.^1,2^ This could be attributed to the particular nature of the electron transfer observed in Be-graphenes, with strong Be ion-graphene substrate interaction evidenced by the strong localization of the transferred electrons. The electron transfer to the system also disrupts the delocalized p orbital characteristic in graphene by introducing these localized states,^1,80,81^ thus opening a band-gap as shown by previous studies. Detailed results for Be-graphenes have been previously presented by our group.^44^ Since the lower energy configuration is the out-of-plane doping configuration for Be, it will be used for further discussion. This more directly corresponds to predictions in work done by Lopez-Urias *et al.*^1^

It must be noted out that in contrast to the previous study by the author's group^44^ and in agreement with the study by Lopez-Urias' team^1^ and by Ullah's team,^2^ a +1.6*e*^−^ charge was observed on Be-graphene in this work instead of +2.0, which would have been indicative of a full electron transfer. This may be attributed to differences in the pseudopotentials used, particularly with the treatment of semicore states. Lopez-Urias and colleagues used Troullier–Martins norm-conserving pseudopotentials,^82,83^ Ullah's group used a PAW-PBE pseudopotential from the Vienna *Ab initio* Software Package^84^ while our group previously used an older version of PSLibrary(ver. 0.2.3).^25^ The older version of PSLibrary did not treat semicore states for Be, thus, this work also updates the accuracy of our previous work.

Further, it must be noted that the work by Lopez-Urias *et al.*^1^ was done on a larger supercell size of 6 × 6 unit cells. Despite these differences, the band structures predicted by their study and this study are remarkably similar (Fig. 6a). The p-type indirect band gap predicted by this study was also observed by Lopez-Urias and colleagues.^1^ There is also very little difference in the predicted band gap, with their study predicting 0.28 eV while this study also predicts 0.28 eV. This may imply that the dopant–dopant interactions for this smaller supercell size have been similarly minimized. Ullah and colleagues^2^, on the other hand, predicted a larger 0.46 eV band gap, which may be attributed to the in-plane configuration they found for beryllium doping in graphene.

In contrast, previous work by Rafique *et al.* using smaller supercell size (3 × 4)^19^ indicated that a half-metallic, and thus spin-polarized electronic properties is predicted for Be-graphene. This may imply that either the supercell size is significant in the predicted band structure, or that the properties of Be-graphene vary at higher doping concentrations (since a smaller supercell size implies a larger dopant-to-substrate ratio). A similar observation on differing supercell sizes accounting for differences in the electronic properties predicted can be made when comparing results between this work and that by Rafique *et al.*, for the other AE-graphenes. In particular, supercell size greatly affects the results of computational studies^85–87^ since this may affect the significance of dopant–dopant interactions on the simulation and hence the predicted properties.^88^ Choosing a larger supercell size may emphasize the dopant–substrate interactions by minimizing dopant–dopant interactions.

The presence of the localized states which opened the band-gap as evinced by Fig. 6b may also be indicative of reduced applicability for electrochemical applications^1^ despite possible increased chemical reactivity due to the highly positive and highly negative sites induced on the material by doping. This is especially considering that electric conductivity is an important aspect to consider when selecting for possible electrocatalysts,^89^ or sensors.^15^ Consistent with possible increased chemical reactivity, Ullah and his colleagues has demonstrated the potential of Be-doping on graphene for lithium-ion battery anodes.^3^ In this work, it was posited that the inclusion of beryllium on graphene makes the graphene material electron deficient, which renders lithium adsorption on the material feasible. This may also imply possible application for beryllium-doped graphenes in this area, not only for lithium-ion batteries but for other batteries where the adsorption of alkali metal ion species is required. Further, application of beryllium as co-dopant^5^ in order to improve these adsorption-related properties may also be pursued.

### 3.4. Magnesium-doped graphene: a Dirac half-metal (DHM)

Mg-graphene also exhibits electron transfer (−1.3*e*^−^), however there is significant spin-polarization observed (Fig. 5a) which can be attributed to the electrons transferred from the Mg dopant to the carbon atoms adjacent to it on the graphene substrate. The total and absolute magnetization for Mg-graphene also indicates nearly two Bohr magnetons (*μ*_B_) of total and absolute magnetization for Mg-graphene, which can be attributed to a triplet pair. Hence, the spin-asymmetry for Mg-graphene can be primarily attributed to the electron transfer induced.

**Fig. 7:**
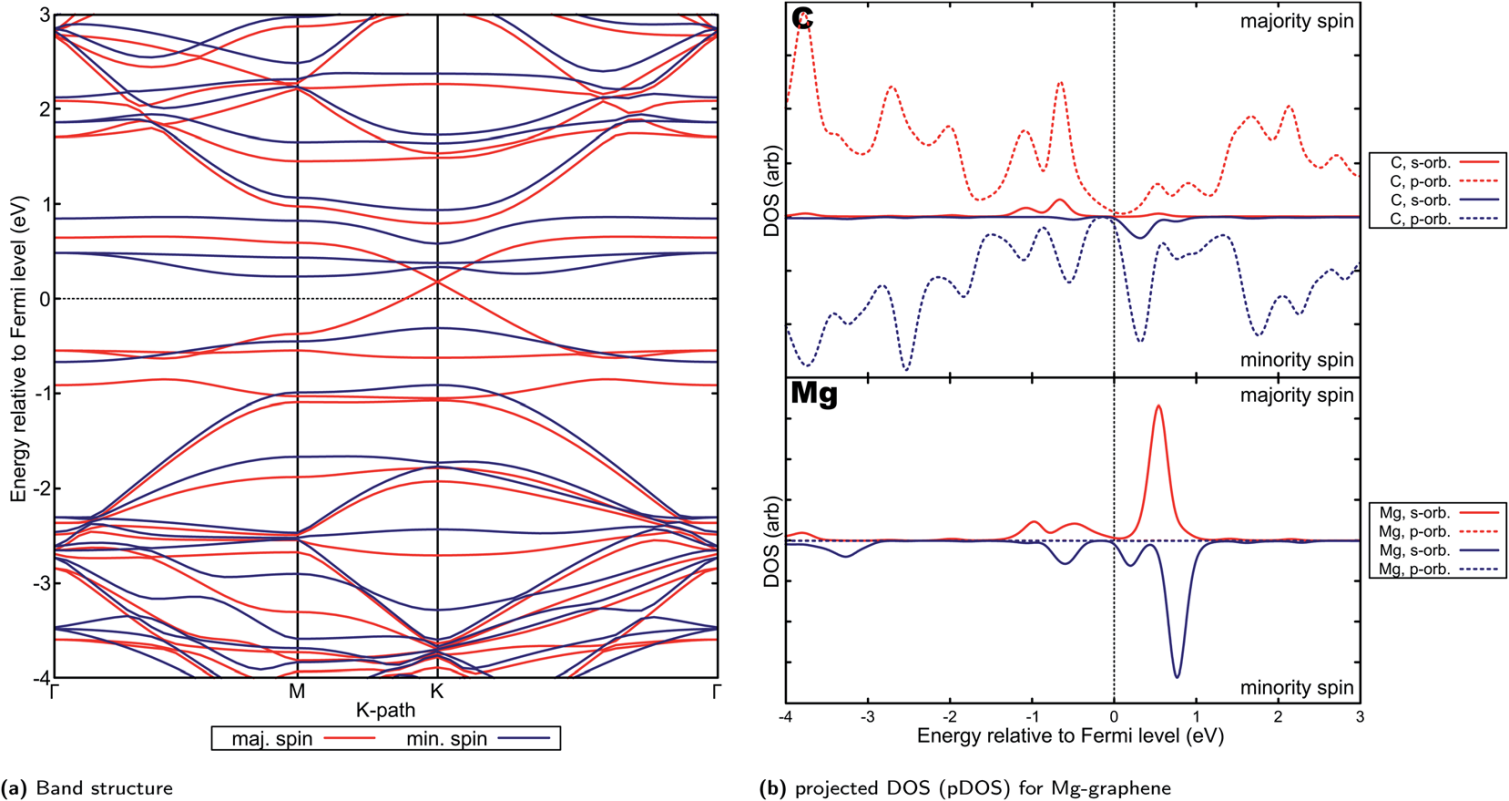
Electronic properties and orbital hybridization for Mg-graphene: (a) band structure and (b) projected density of states (pDOS). Red indicates majority spin (spin-up) states and blue indicates minority spin (spin down) states. ESI for electronic properties at Fig. S11.† Results at higher cutoff available at Fig. H16.†

This electron transfer has significant influence on the observed electronic properties of Mg-graphene. The VBM (valence band maximum) and CBM (conduction band minimum) partial charge density for the majority spin (Fig. S11b†) indicates that the main contribution to both the VBM and the CBM is delocalized, *i.e.*, states all over the AE-graphene contributes to both the VBM and CBM. Further, this VBM charge density has little overlap with the corresponding CBM charge density, possibly indicating a lower degree of carrier recombination.^90^ This observation for the VBM and CBM partial charge density is similar to the observation by Shen and colleagues for pristine 2-dimensional stanene^91^ and may explain the p-type Dirac cone-like structure observed for the majority spin band structure (Fig. 7a) in Mg-graphene. In this case, the p-type behavior is due to fewer electrons on the Mg dopant *versus* the carbon atom, which acts essentially an induced hole in Mg-graphene. The Dirac point observed for Mg-graphene lies approximately 0.192 eV above the Fermi level.

This p-type Dirac cone-like feature is similar to that observed by Gierz and her team for epitaxial graphene on SiC modified by adsorbed gold atoms.^92^ Further, other two-dimensional materials, such as nickel–carbon–hydrogen and cobalt–carbon–hydrogen metal–organic frameworks also show this “half-metallic Dirac cone” behavior.^93^ These materials which exhibit a Dirac cone-like feature in only one spin are collectively known as Dirac half-metals (DHMs).^94^ DHMs are promising for spintronic applications since they combine spin polarization and massless Dirac charge carriers. Further, they “are also capable of generating 100% spin-polarization and massless Dirac fermions, which are expected to perform better than other spintronic materials…”.^95^ At least one patent has already been applied for with regards to the utilization of DHMs for spintronic applications,^96^ presumably using manganese trihalides.^97^ Experimentally, a DHM has already been observed in the form of Mn_2_CoAl, a Heusler alloy.^98^ In the case of transition metal-containing DHMs, the magnetism was primarily attributed to the metal atom and their d-orbitals.

In contrast, since there is no atom in Mg-graphene introducing d-orbital states, the magnetism or spin asymmetry is borne by electrons attributed to carbon atoms in the substrate and may be attributed to polarization caused by the electron deficient and hence positive Mg ion. Similar phenomena was also observed in work on C_3_Ca_2_ films, where p-orbital magnetization was shown to be primarily responsible for spin asymmetry.^99^ It is also posited that a wider band gap implies greater stability for the Dirac half-metal behavior, *i.e.*, the Dirac half-metal behavior is kept at a wider range of applied voltages. This may imply that Mg-graphenes, should they be applicable for this application, would be limited to a narrower range of applied voltages, or thus limited to low-power applications. Na_2_C was also estimated to be a low band gap DHM^100^ at around 0.7 eV. Other compounds using beryllium and carbon (Be_3_C_2_)^101^ and magnesium and carbon (Mg_3_C_2_)^102^ were also shown to be Dirac half metals. It must be noted that most of these materials have a high concentration of alkali or alkaline earth metals compared to the Mg-graphene system considered in this work. Thus, this electronic structure may be said to emerge from interaction between the alkaline earth atom and carbon and may provide a pathway for developing DHMs if further investigated.

The projected DOS for Mg-graphene (Fig. 7b) further shows that the unoccupied s-orbital of Mg induces the Dirac cone for the p orbital of carbon at that energy level close to the s-orbital energy level of Mg, consistent with the observation of partial charge density for the CBM on the Mg atom. This is also supported by the partial electron density for the VBM and CBM and projected DOS of Mg-graphene (Fig. 7b, S11b† and S11c†), which shows p-orbital-shaped states being the main portions of the electron density accounting for the VBM and the CBM. This also implies that it is expected for holes to be the predominant carrier in the majority spin for Mg-graphene should no voltage be applied. Further, the Dirac cone-like feature for the majority spin band structure may also indicate that these majority-spin carriers can possibly travel in a ballistic manner.

On the other hand, the VBM and CBM partial charge density for the minority spin are more strongly localized in the region near the dopant atom and has more overlap (Fig. S11c†) This supports the observation of an indirect, n-type band gap in the minority spin for Mg-graphene. The localization shown by the VBM and CBM charge densities are also consistent with the projected DOS in that most of the contributions are those of hybridized states for the CBM, while p orbital contributions dominate for the VBM.

Spin-asymmetry may further explain the n-type behavior of Mg-graphene for the minority spin. This is because it would be more feasible, following Pauli's exclusion principle, for any electron to be introduced to the system to form singlets or pair up with the unpaired majority spin electrons in the system. In other words, it may be more energetically feasible for the system to accept minority spin electrons over majority spin electrons when electrons are introduced to the system, such as *via* an applied potential. This is reflected in the higher density of states (Fig. S11a†) for the minority spin at energy levels just above the Fermi level compared to the majority spin, whose lowest unoccupied states sit at a higher energy level. Thus, using the converse logic, the p-type behavior of the majority spin may be explained.

### 3.5. Calcium-doped graphene and strontium-doped graphene: bipolar magnetic semiconductors

Ca- and Sr-graphenes were also found to have similar (approx. 1.3*e*^−^) electron transfer characteristics with the rest of AE-graphenes. Similar to Mg-graphenes (and in contrast to Be-graphenes), this charge transfer has also induced a triplet state to arise (see Fig. 5b and c) attributed to the electrons transferred to the dopant which caused ferromagnetic properties.

**Fig. 8:**
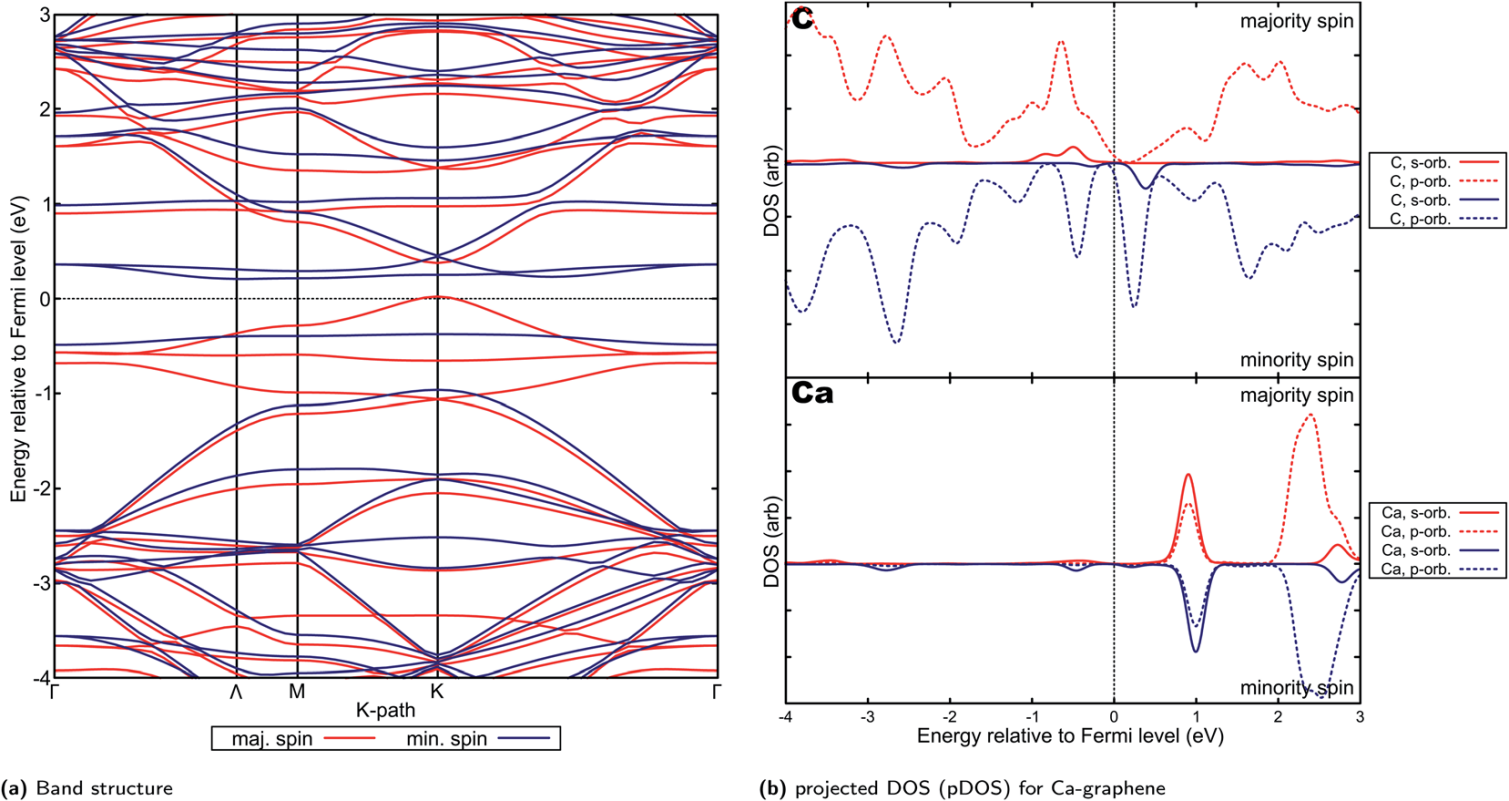
Electronic properties and orbital hybridization for Ca-graphene: (a) band structure and (b) projected density of states (pDOS). Red indicates majority spin (spin up) states and blue indicates minority spin (spin down) states. ESI for electronic properties at Fig. S12.† Results at higher cutoff available at Fig. H18.†

**Fig. 9:**
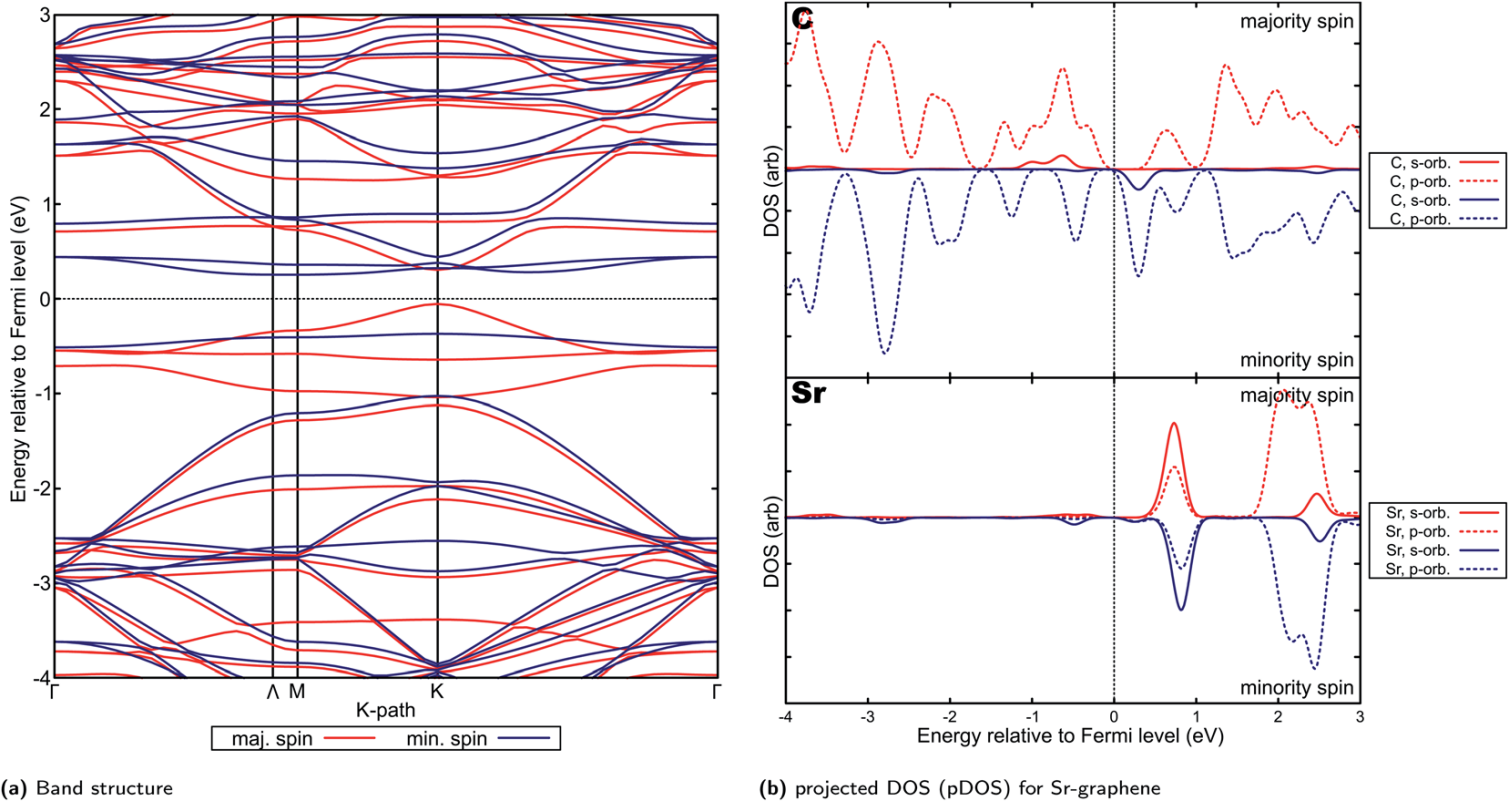
Electronic properties and orbital hybridization for Sr-graphene: (a) band structure and (b) projected density of states (pDOS). Red indicates majority spin (spin up) states and blue indicates minority spin (spin down) states. ESI for electronic properties at Fig. S13.† Results at higher cutoff available at Fig. H20.†

For Ca- and Sr-graphene, similar spin-asymmetric band structures have been observed (Fig. 8a for Ca and Fig. 9a for Sr). It can be noted that for both Ca- and Sr-graphenes, a p-type direct band gap was observed in the majority spin. In contrast, n-type indirect and larger band gaps wider than the majority spin band gaps were observed in the minority spin. It must also be noted that the point at which the VBM occurs differ slightly between Ca, but it is generally found between *Γ* and *M*. At the higher cutoff (Fig. H20†), the point in *K*-space where the VBM occurs for Sr can also be found in between *Γ* and *M*. This may be attributed to the flatness of the introduced minority spin state as observed in the band structure.

This spin-asymmetric band structure is consistent with observed ferromagnetic behavior for Ca- and Sr-graphenes. Hence, these materials may be classified as bipolar magnetic semiconductors,^103^ since there is an asymmetric band gap from the majority and minority spins, as well as p-type and n-type asymmetry between spins.

Bipolar magnetic semiconductors have promising applications for spintronic devices. The asymmetry of the band gap may allow for spin-polarization control *via* an applied gate voltage.^104^ This is in contrast to half-metals and spin-gapless semiconductors where spin-polarization is inherently fixed in one spin. Similar two-dimensional materials have been investigated, such as CrSiTe_3_,^105^ semi-hydrogenated single walled carbon nanotubes^103^ and hydrogenated bilayer wurtzite nanofilms.^106^ MnPSe_3_ nanosheets have been predicted and experimentally verified to exhibit bipolar magnetic semiconductor property.^107,108^ The relatively narrow band-gaps for these materials may also imply possible applicability in low-power applications.

Projected DOS for both Ca- and Sr-graphenes (Fig. 8b and 9b) indicate that for the majority spin, p-orbital contributions dominate, while for the minority spin, localization caused by s–p hybridization opens the band gap. This is consistent with observations for VBM and CBM charge densities (Fig. S12b, S12c, S13b and S13c†). This also is similar to the observations for Mg-graphene; further, the spin asymmetry also induced the n-type behavior for the minority spin and p-type behavior for the majority spin. Contrastingly, the Dirac cone-like feature was broken for the majority spin in both Ca and Sr-graphenes inducing the p-type direct semiconductor band structure. This may be attributed to the much higher atomic number of Ca and Sr *versus* Mg since it may cause the steric hindrance to be more prominent, as qualitatively demonstrated by the RDG plots (Fig. 4). The similarity between the VBM and CBM charge densities for the majority spin (similar p-orbital contributions) may explain the direct band gap observed for these materials in that spin and further imply that the magnetization can be primarily attributed to p-orbital magnetism.

## 4. Conclusions

This work has investigated and characterized alkaline earth doped graphenes as a new class of carbon-based nanomaterials *via* density functional theory calculations. Substitutional doping of alkaline-earth doped graphenes was found to be energetically plausible, indicating its viability as a doping motif or configuration on graphene. Substitutionally doping an alkaline earth metal atom on graphene was found to cause electron transfer from the alkaline earth dopant to the graphene substrate.

This was found to be the main mode of interaction between the alkaline earth atom dopant and graphene. Ionic bonding due to this electron transfer was generally predicted for AE-graphenes, with Be-graphene having a variant bonding characteristic due to its low atomic number. This variation was explained as being due to electronegativity equalization. This model for bonding was able to help explain the energetic trends observed for the materials as being consistent with predictions by Liu, Merinov and Goddard,^75^ with the weakest substrate binding also observed for Mg-graphene. This may help guide the utilization of alkaline earth atoms in batteries and other applications relying on the use of these atoms as ions.

The electron transfer induces sites with high positive and negative charge on AE-graphenes and also causes shifts in the work function for AE-graphenes. For Be-graphene, the work function increased *versus* pristine graphene, while for the rest of AE-graphenes, work function lowering was predicted. This work function lowering was found to be consistent with experimental work on calcium-doped graphene^17^ and may imply that the strong C–Ca bonding observed in their work may contribute to the observed work function lowering.

Electron transfer on AE-graphenes induce spin-asymmetric or possible magnetic properties with the exception of Be-graphene which is predicted to have no magnetization in consonance with previous work and due to the variant bonding characteristic. The carbon atoms adjacent to the alkaline earth dopant for the magnetic AE-graphenes were found to have a high spin density and are thus where the spin-polarization mostly occurs. Mg-graphene was predicted to be a Dirac half-metal (DHM), while Ca- and Sr-graphenes were predicted to be bipolar magnetic semiconductors. These novel electronic properties may prove to be interesting for applications involving magnetic devices and spintronic devices.

Hence, it may be worthwhile to further explore magnetism in AE-graphenes, particularly by estimating thermal effects such as the Curie temperature of AE-graphenes. More rigorous computations which may better account for magnetism in materials can be done to verify predictions. More importantly, since Dirac half-metals and bipolar magnetic semiconductors are still developing families of materials,^109^ experimental verification and further discovery for these materials will be significant. These in turn will provide for more realistic models derived from experimentally observed structures for further theoretical work and predictions.

## Conflicts of interest

There are no conflicts to declare.

## Supplementary Material

RA-011-D0RA08115A-s001

RA-011-D0RA08115A-s002

## References

[cit1] Lopez-Urias F., Terrones M., Terrones H. (2015). Carbon.

[cit2] Ullah S., Hussain A., Syed W., Saqlain M. A., Ahmad I., Leenaerts O., Karim A. (2015). RSC Adv..

[cit3] Ullah S., Denis P. A., Sato F. (2017). Appl. Mater. Today.

[cit4] Ullah S., Denis P. A., Sato F. (2018). Current Graphene Science.

[cit5] Ullah S., Denis P. A., Sato F. (2019). Int. J. Quantum Chem..

[cit6] Olsson E., Hussain T., Karton A., Cai Q. (2020). Carbon.

[cit7] Chen C., Zhang J., Zhang B., Ming Duan H. (2013). J. Phys. Chem. C.

[cit8] Wen Y., Xie F., Liu X., Liu X., Chen R., Cho K., Shan B. (2017). Int. J. Hydrogen Energy.

[cit9] Luo H., Li H., Fu Q. (2017). Chem. Phys. Lett..

[cit10] Olaniyan O., Mapasha R. E., Momodu D. Y., Madito M. J., Kahleed A. A., Ugbo F. U., Bello A., Barzegar F., Oyedotun K., Manyala N. (2016). RSC Adv..

[cit11] Olaniyan O., Maphasha R., Madito M., Khaleed A., Igumbor E., Manyala N. (2018). Carbon.

[cit12] Olaniyan O., Igumbor E., Khaleed A. A., Mirghni A. A., Manyala N. (2019). AIP Adv..

[cit13] Rafique M., Mirjat N. H., Soomro A. M., Khokhar S., Shuai Y. (2018). Phys. Lett. A.

[cit14] Muhammad R., Shuai Y., Tan H.-P. (2017). J. Mater. Chem. C.

[cit15] Luo H., Zhang L., Xu S., Shi M., Wu W., Zhang K. (2020). Appl. Surf. Sci..

[cit16] Delley B. (2000). J. Chem. Phys..

[cit17] Klain C., Linde S., Shikler R., Sarusi G. (2020). Carbon.

[cit18] Han Y., Chen Y., Wang N., He Z. (2019). Mater. Technol..

[cit19] Rafique M., Shuai Y., Tan H.-P., Hassan M. (2017). RSC Adv..

[cit20] Arsent'ev M. Y., Prikhodko A. V., Shmigel A. V., Egorova T. L., Kalinina M. V. (2015). J. Phys.: Conf. Ser..

[cit21] Giannozzi P., Baroni S., Bonini N., Calandra M., Car R., Cavazzoni C., Ceresoli D., Chiarotti G. L., Cococcioni M., Dabo I., Dal Corso A., de Gironcoli S., Fabris S., Fratesi G., Gebauer R., Gerstmann U., Gougoussis C., Kokalj A., Lazzeri M., Martin-Samos L., Marzari N., Mauri F., Mazzarello R., Paolini S., Pasquarello A., Paulatto L., Sbraccia C., Scandolo S., Sclauzero G., Seitsonen A. P., Smogunov A., Umari P., Wentzcovitch R. M. (2009). J. Phys.: Condens. Matter.

[cit22] Giannozzi P., Andreussi O., Brumme T., Bunau O., Buongiorno Nardelli M., Calandra M., Car R., Cavazzoni C., Ceresoli D., Cococcioni M., Colonna N., Carnimeo I., Dal Corso A., de Gironcoli S., Delugas P., DiStasio R. A., Ferretti A., Floris A., Fratesi G., Fugallo G., Gebauer R., Gerstmann U., Giustino F., Gorni T., Jia J., Kawamura M., Ko H.-Y., Kokalj A., Küçükbenli E., Lazzeri M., Marsili M., Marzari N., Mauri F., Nguyen N. L., Nguyen H.-V., Otero-de-la Roza A., Paulatto L., Poncé S., Rocca D., Sabatini R., Santra B., Schlipf M., Seitsonen A. P., Smogunov A., Timrov I., Thonhauser T., Umari P., Vast N., Wu X., Baroni S. (2017). J. Phys.: Condens. Matter.

[cit23] Blöchl P. E. (1994). Phys. Rev. B: Condens. Matter Mater. Phys..

[cit24] Perdew J. P., Burke K., Ernzerhof M. (1996). Phys. Rev. Lett..

[cit25] Dal Corso A. (2014). Comput. Mater. Sci..

[cit26] Monkhorst H. J., Pack J. D. (1976). Phys. Rev. B: Solid State.

[cit27] Bengtsson L. (1999). Phys. Rev. B: Condens. Matter Mater. Phys..

[cit28] Momma K., Izumi F. (2011). J. Appl. Crystallogr..

[cit29] Kokalj A. (1999). J. Mol. Graphics Modell..

[cit30] Bader R. F. W. , Atoms in molecules: a quantum theory, Clarendon Press, Oxford, 2003

[cit31] Tang W., Sanville E., Henkelman G. (2009). J. Phys.: Condens. Matter.

[cit32] Henkelman G., Arnaldsson A., Jónsson H. (2006). Comput. Mater. Sci..

[cit33] Sanville E., Kenny S. D., Smith R., Henkelman G. (2007). J. Comput. Chem..

[cit34] Becke A. D., Edgecombe K. E. (1990). J. Chem. Phys..

[cit35] Savin A., Jepsen O., Flad J., Andersen O. K., Preuss H., von Schnering H. G. (1992). Angew. Chem., Int. Ed. Engl..

[cit36] Contreras-Garcia J., Johnson E. R., Keinan S., Chaudret R., Piquemal J.-P., Beratan D. N., Yang W. (2011). J. Chem. Theory Comput..

[cit37] Silvi B., Savin A. (1994). Nature.

[cit38] Koumpouras K., Larsson J. A. (2020). J. Phys.: Condens. Matter.

[cit39] Chaudret R., de Courcy B., Contreras-Garcia J., Gloaguen E., Zehnacker-Rentien A., Mons M., Piquemal J.-P. (2014). Phys. Chem. Chem. Phys..

[cit40] Fang D., Chaudret R., Piquemal J.-P., Cisneros G. A. (2013). J. Chem. Theory Comput..

[cit41] Gillet N., Chaudret R., Contreras-Garcia J., Yang W., Silvi B., Piquemal J.-P. (2012). J. Chem. Theory Comput..

[cit42] Savin A., Nesper R., Wengert S., Fässler T. F. (1997). Angew. Chem., Int. Ed. Engl..

[cit43] Miller G. J. , Zhang Y. and Wagner F. R., Handbook of Solid State Chemistry, Wiley-VCH Verlag GmbH & Co. KGaA, Weinheim, Germany, 2017, pp. 405–489

[cit44] Serraon A. C. F., Padama A. A. B., del Rosario J. A. D., Ocon J. D. (2017). ECS Trans..

[cit45] Ishii A., Yamamoto M., Asano H., Fujiwara K. (2008). J. Phys.: Conf. Ser..

[cit46] Kwon K. C., Choi K. S., Kim S. Y. (2012). Adv. Funct. Mater..

[cit47] Martínez-Orozco R., Rosu H., Lee S.-W., Rodríguez-González V. (2013). J. Hazard. Mater..

[cit48] Rut'kov E., Afanas'eva E., Gall N. (2020). Diamond Relat. Mater..

[cit49] Yoon T., Wu Q., Yun D.-J., Kim S. H., Song Y. J. (2020). Sci. Rep..

[cit50] Yan R., Zhang Q., Li W., Calizo I., Shen T., Richter C. A., Hight-Walker A. R., Liang X., Seabaugh A., Jena D., Grace Xing H., Gundlach D. J., Nguyen N. V. (2012). Appl. Phys. Lett..

[cit51] Takahashi T., Tokailin H., Sagawa T. (1985). Phys. Rev. B: Condens. Matter Mater. Phys..

[cit52] Shi Y., Kim K. K., Reina A., Hofmann M., Li L.-J., Kong J. (2010). ACS Nano.

[cit53] Oshima C., Nagashima A. (1997). J. Phys.: Condens. Matter.

[cit54] Syu J.-Y., Chen Y.-M., Xu K.-X., He S.-M., Hung W.-C., Chang C.-L., Su C.-Y. (2016). RSC Adv..

[cit55] Shan B., Cho K. (2005). Phys. Rev. Lett..

[cit56] Hu T., Gerber I. C. (2013). J. Phys. Chem. C.

[cit57] Nishidate K., Yoshimoto N., Chantngarm P., Saito H., Hasegawa M. (2016). Mol. Phys..

[cit58] Bae G., Jung H., Park N., Park J., Hong S., Park W. (2012). Appl. Phys. Lett..

[cit59] Hu W., Li Z., Yang J. (2013). J. Chem. Phys..

[cit60] Yang N., Yang D., Chen L., Liu D., Cai M., Fan X. (2017). Nanoscale Res. Lett..

[cit61] Giovannetti G., Khomyakov P. A., Brocks G., Karpan V. M., van den Brink J., Kelly P. J. (2008). Phys. Rev. Lett..

[cit62] Choi S.-M., Jhi S.-H., Son Y.-W. (2010). Phys. Rev. B: Condens. Matter Mater. Phys..

[cit63] Leenaerts O., Partoens B., Peeters F. M., Volodin A., Van Haesendonck C. (2017). J. Phys.: Condens. Matter.

[cit64] Hibino H., Kageshima H., Kotsugi M., Maeda F., Guo F.-Z., Watanabe Y. (2009). Phys. Rev. B: Condens. Matter Mater. Phys..

[cit65] Zan R., Ramasse Q. M., Bangert U., Novoselov K. S. (2012). Nano Lett..

[cit66] Chen J., Shi T., Cai T., Xu T., Sun L., Wu X., Yu D. (2013). Appl. Phys. Lett..

[cit67] Patra A., Bates J. E., Sun J., Perdew J. P. (2017). Proc. Natl. Acad. Sci..

[cit68] Legesse M., Mellouhi F. E., Bentria E. T., Madjet M. E., Fisher T. S., Kais S., Alharbi F. H. (2017). Appl. Surf. Sci..

[cit69] Klein C., Cohen-Elias D., Sarusi G. (2018). Heliyon.

[cit70] York D. M., Yang W. (1996). J. Chem. Phys..

[cit71] Goudarzi M., Parhizgar S., Beheshtian J. (2019). Opto-Electron. Rev..

[cit72] Ullah S., Denis P. A., Sato F. (2017). ChemPhysChem.

[cit73] Savin A., Silvi B., Colonna F. (1996). Can. J. Chem..

[cit74] Mo Y., Gao J. (2001). J. Phys. Chem. A.

[cit75] Liu Y., Merinov B. V., Goddard W. A. (2016). Proc. Natl. Acad. Sci..

[cit76] Gonzalez-Herrero H., Gomez-Rodriguez J. M., Mallet P., Moaied M., Palacios J. J., Salgado C., Ugeda M. M., Veuillen J.-Y., Yndurain F., Brihuega I. (2016). Science.

[cit77] Li Z., Xie W., Liu X., Wu Y. (2015). J. Appl. Phys..

[cit78] Volnianska O., Boguslawski P. (2010). J. Phys.: Condens. Matter.

[cit79] Zhang W., Song Z., Peng B., Zhang W. (2012). J. Appl. Phys..

[cit80] Deringer V. L., Tchougréeff A. L., Dronskowski R. (2011). J. Phys. Chem. A.

[cit81] Dronskowski R. , Computational chemistry of solid state materials: a guide for materials scientists, chemists, physicists and others, Wiley-VCH, Weinheim, 2005

[cit82] Troullier N., Martins J. L. (1991). Phys. Rev. B: Condens. Matter Mater. Phys..

[cit83] Kleinman L., Bylander D. M. (1982). Phys. Rev. Lett..

[cit84] Kresse G., Hafner J. (1994). Phys. Rev. B: Condens. Matter Mater. Phys..

[cit85] Schwarz K. (2006). Proc. Natl. Acad. Sci..

[cit86] Hine N. D. M., Frensch K., Foulkes W. M. C., Finnis M. W. (2009). Phys. Rev. B: Condens. Matter Mater. Phys..

[cit87] Castleton C. W. M., Hoglund A., Mirbt S. (2009). Modell. Simul. Mater. Sci. Eng..

[cit88] Deretzis I. and La Magna A., GraphITA, Springer International Publishing, Cham, 2017, pp. 61–69

[cit89] Lee J.-H., Park S.-j., Choi J.-W. (2019). Nanomaterials.

[cit90] Li D., Liang C., Zhang H., Zhang C., You F., He Z. (2015). J. Appl. Phys..

[cit91] Shen L., Lan M., Zhang X., Xiang G. (2017). RSC Adv..

[cit92] Gierz I., Riedl C., Starke U., Ast C. R., Kern K. (2008). Nano Lett..

[cit93] Ma Y., Dai Y., Li X., Sun Q., Huang B. (2014). Carbon.

[cit94] Ishizuka H., Motome Y. (2012). Phys. Rev. Lett..

[cit95] Ma F., Jiao Y., Jiang Z., Du A. (2018). ACS Appl. Mater. Interfaces.

[cit96] Kioussis N. and Sun Q., US Pat. Application, 20190304652, 2019

[cit97] Sun Q., Kioussis N. (2018). Phys. Rev. B.

[cit98] Ouardi S., Fecher G. H., Felser C., Kübler J. (2013). Phys. Rev. Lett..

[cit99] Ji W.-x., Zhang B.-m., Zhang S.-f., Zhang C.-w., Ding M., Li P., Wang P.-j. (2017). J. Mater. Chem. C.

[cit100] Bafekry A., Mortazavi B., Shayesteh S. F. (2019). J. Magn. Magn. Mater..

[cit101] Wang B., Yuan S., Li Y., Shi L., Wang J. (2017). Nanoscale.

[cit102] Pan H., Han Y., Li J., Zhang H., Du Y., Tang N. (2018). Phys. Chem. Chem. Phys..

[cit103] Li X., Wu X., Li Z., Yang J., Hou J. G. (2012). Nanoscale.

[cit104] Li X., Yang J. (2013). Phys. Chem. Chem. Phys..

[cit105] Cheng H., Zhou J., Yang M., Shen L., Linghu J., Wu Q., Qian P., Feng Y. P. (2018). J. Mater. Chem. C.

[cit106] Yuan L., Li Z., Yang J. (2013). Phys. Chem. Chem. Phys..

[cit107] Du K.-z., Wang X.-z., Liu Y., Hu P., Utama M. I. B., Gan C. K., Xiong Q., Kloc C. (2016). ACS Nano.

[cit108] Li X., Wu X., Yang J. (2014). J. Am. Chem. Soc..

[cit109] Li X., Yang J. (2016). Natl. Sci. Rev..

