# Supplementary material for: Alkaline earth atom doping-induced changes in the electronic and magnetic properties of graphene: a density functional theory study
Source: RSC Adv. 2021 Feb 3;11(11):6268–83. doi: 10.1039/d0ra08115a (PMC8694801; doi:10.1039/d0ra08115a)
Supplement: RA-011-D0RA08115A-s001 [file RA-011-D0RA08115A-s001.pdf]

Electronic Supplementary Material (ESI) for RSC Advances.
This journal is © The Royal Society of Chemistry 2021

Electronic Supplementary Information

List of Figures

S1 Convergence tests for k-point sampling and cutoff energy (ecutwfc) . . . 2
S2 Bader charge analysis and structure for Be-graphene . . . 3
S3 Bader charge analysis and structure for Mg-graphene . . . 4
S4 Bader charge analysis and structure for Ca-graphene . . . 5
S5 Bader charge analysis and structure for Sr-graphene . . . 6
S6 Plane averaged electrostatic potential plots for AE-graphenes . . . 7
S7 Supplementary charge difference plots for AE-graphenes . . . 8
S8 Supplementary electron localization function (ELF) plots for AE-graphenes . . . 9
S9 Supplementary reduced density gradient (RDG) plots for AE-graphenes . . . 10
S10 DOS for Be-graphene. . . . 11
S11 DOS and VBM - CBM partial charge densities for Mg-graphene . . . 12
S12 DOS and VBM - CBM partial charge densities for Ca-graphene . . . 13
S13 DOS and VBM - CBM partial charge densities for Sr-graphene . . . 14

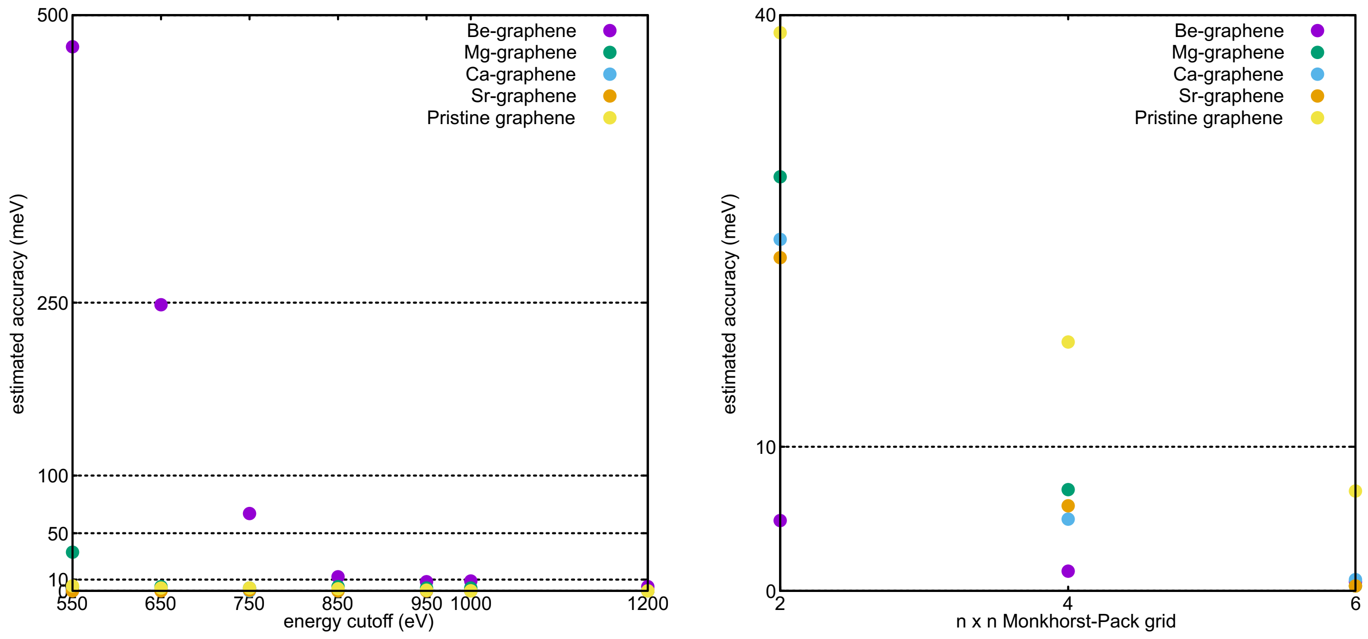

(a) a. energy cutoff

(b) b. k-space sampling

Fig. S1 Convergence tests for k-point sampling and cutoff energy (ecutwfc). Convergence tests for ecutwfc was done versus calculations at 1500 eV cutoff energy. Convergence tests for k-points were done vs 8 x 8 Γ-centered Monkhorst-Pack grid k-space sampling

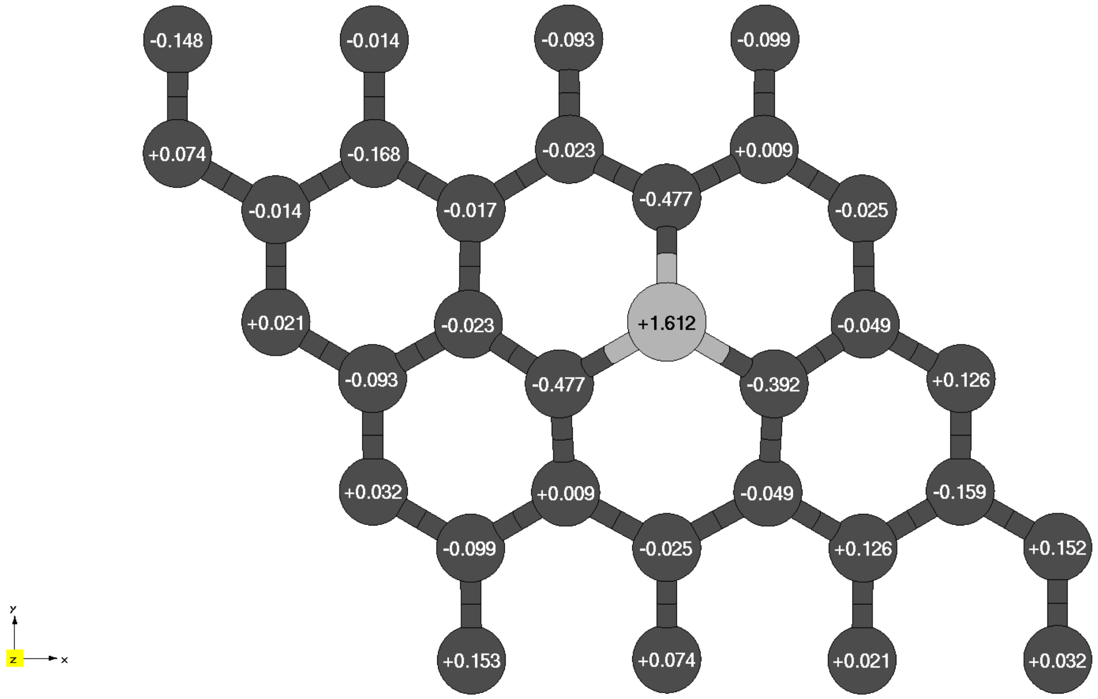

(a) top view

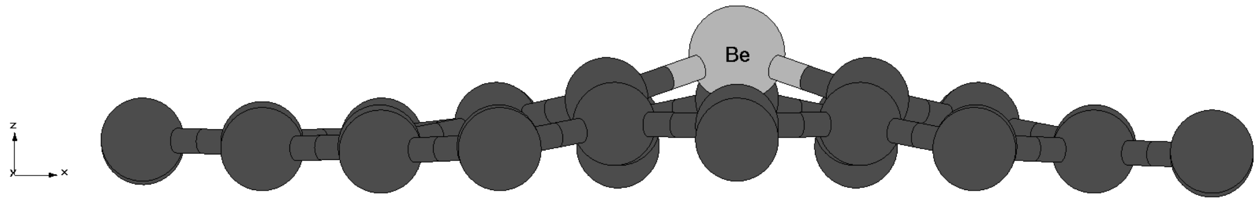

(b) side view

Fig. S2 Bader charge analysis and structure for Be-graphene

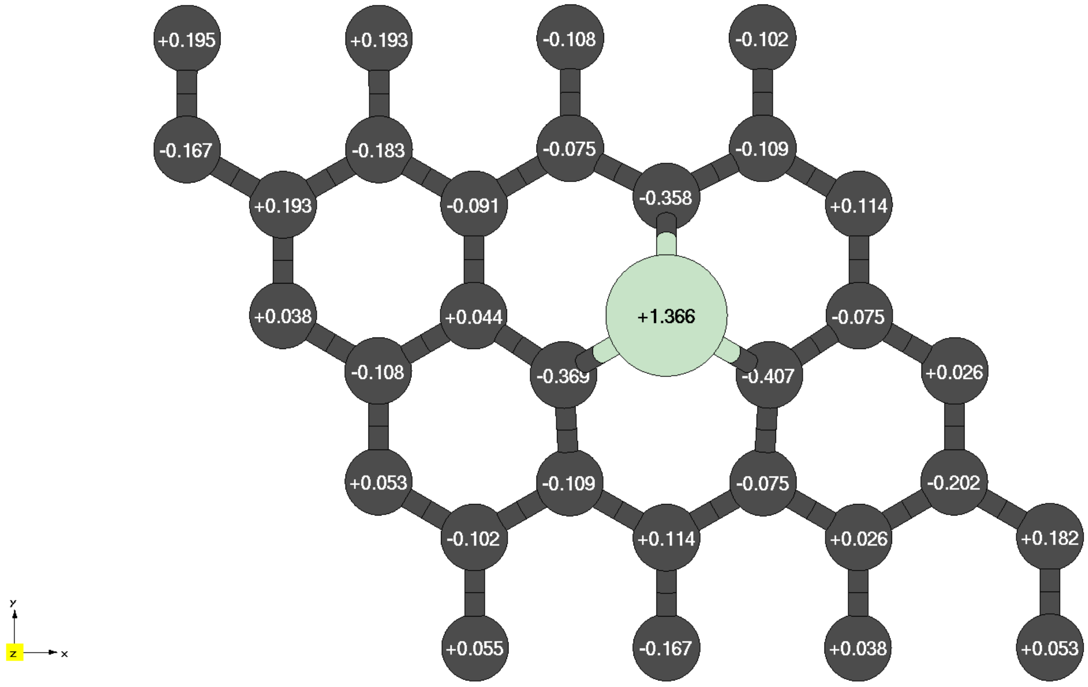

(a) top view

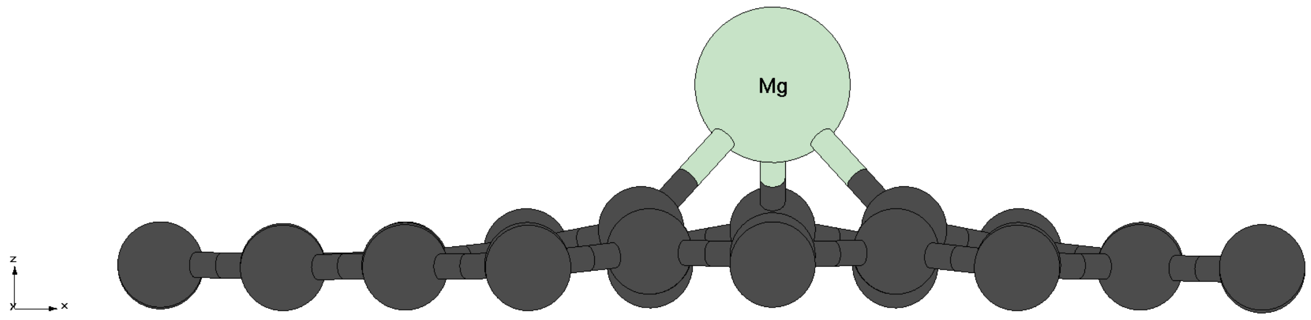

(b) side view

Fig. S3 Bader charge analysis and structure for Be-graphene

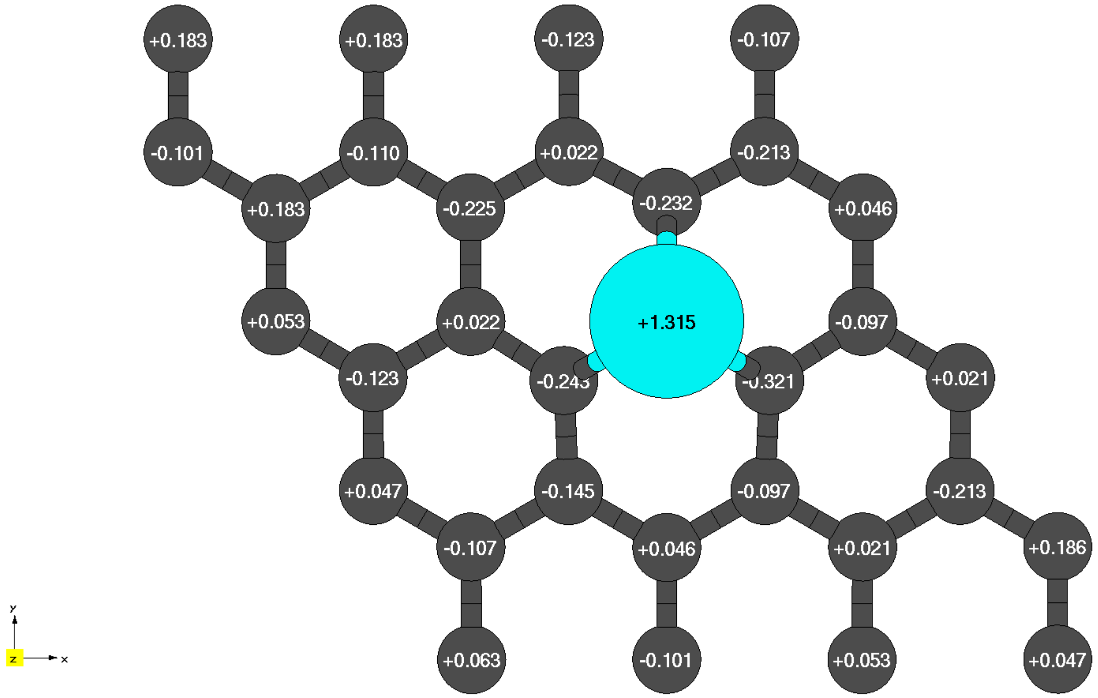

(a) top view

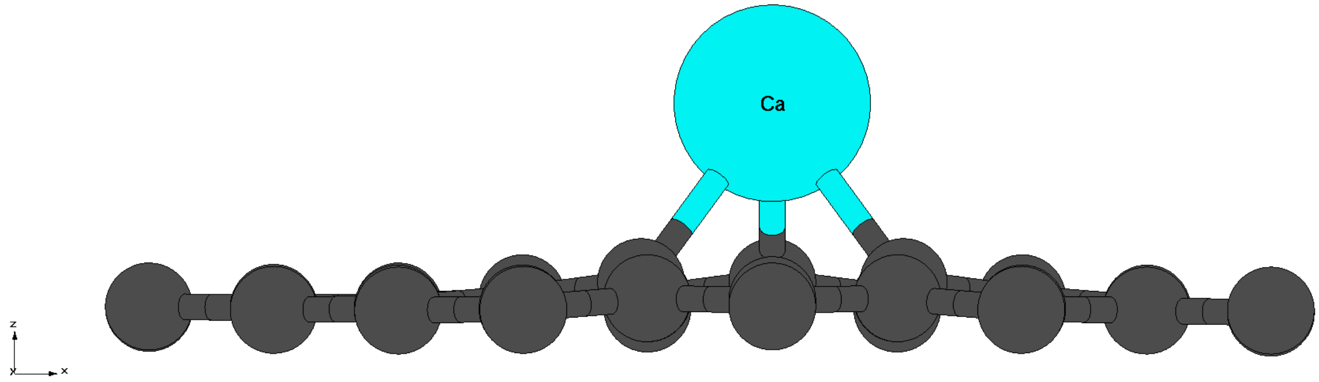

(b) side view

Fig. S4 Bader charge analysis and structure for Ca-graphene

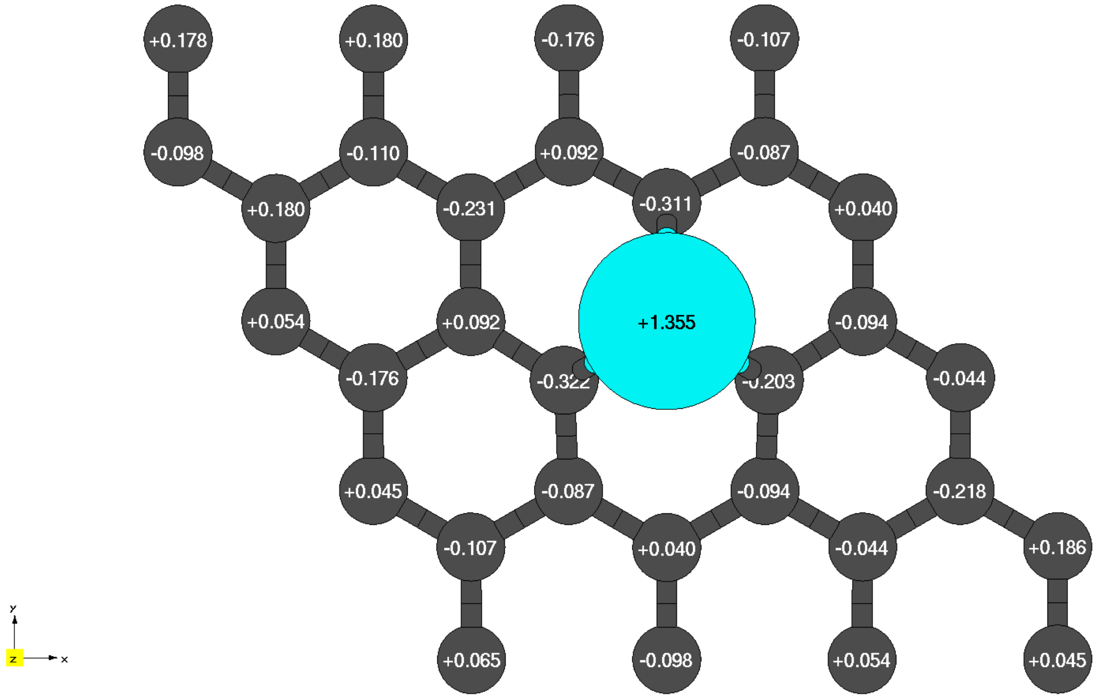

(a) top view

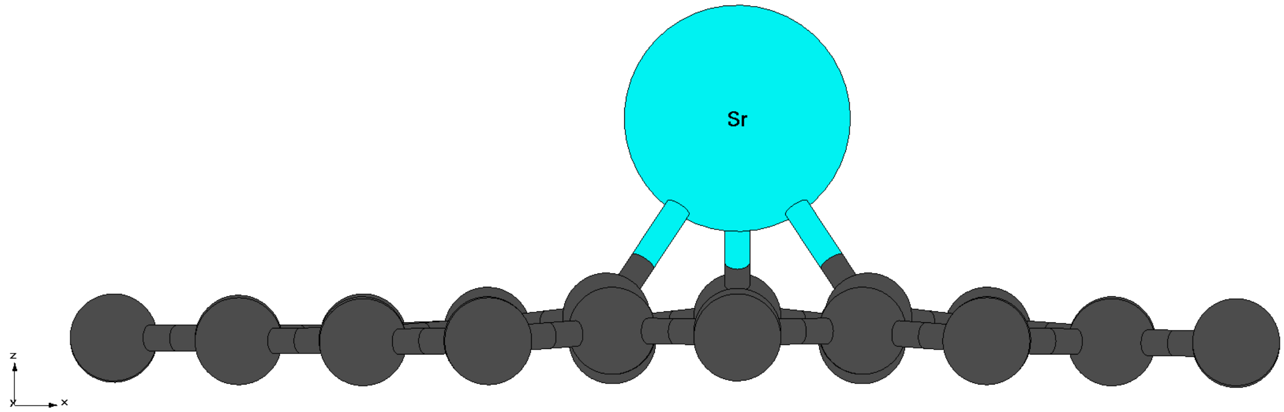

(b) side view

Fig. S5 Bader charge analysis and structure for Sr-graphene

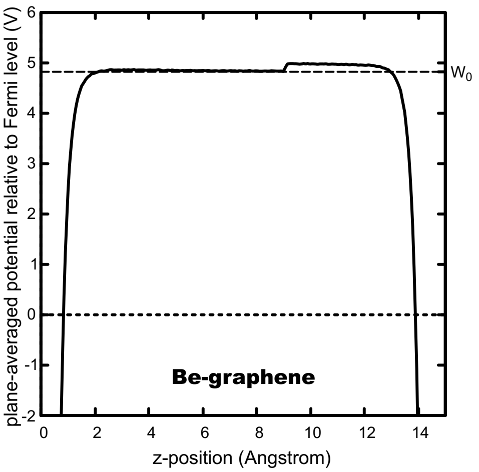

(a) Be-graphene. Workfunction (W_0) = 4.806 eV.
Workfunction with respect to pristine graphene = +0.541 eV

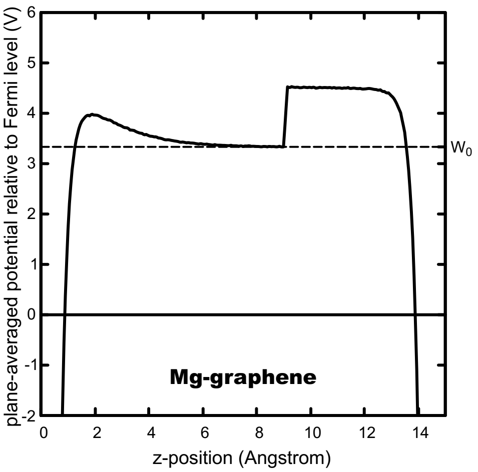

(b) Mg-graphene. Workfunction (W_0) = 3.136 eV.
Workfunction with respect to pristine graphene = -1.129 eV

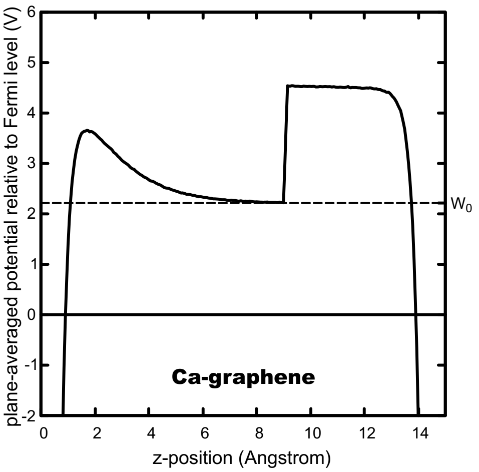

(c) Ca-graphene. Workfunction (W_0) = 2.053 eV.
Workfunction with respect to pristine graphene = -2.212 eV.

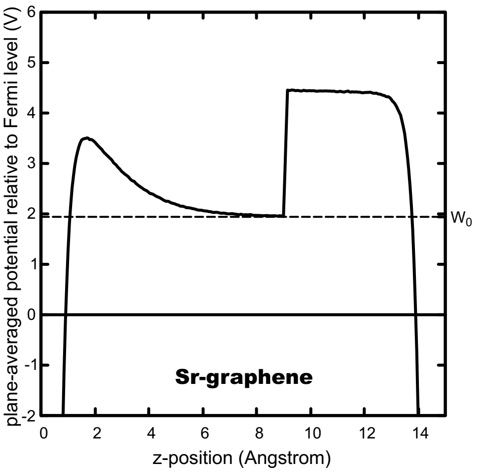

(d) Sr-graphene. Workfunction (W_0) = 1.815 eV.
Workfunction with respect to pristine graphene = -2.450 eV.

Fig. S6 Plane averaged electrostatic potential plots for AE-graphenes. Dashed line indicates the vacuum level (E_{vac}) at the dopant-containing side of graphene. V = 0V for the plots is set at Fermi level (E_{Fermi}) for each AE-graphene. Dopant is oriented towards the positive z-axis direction.

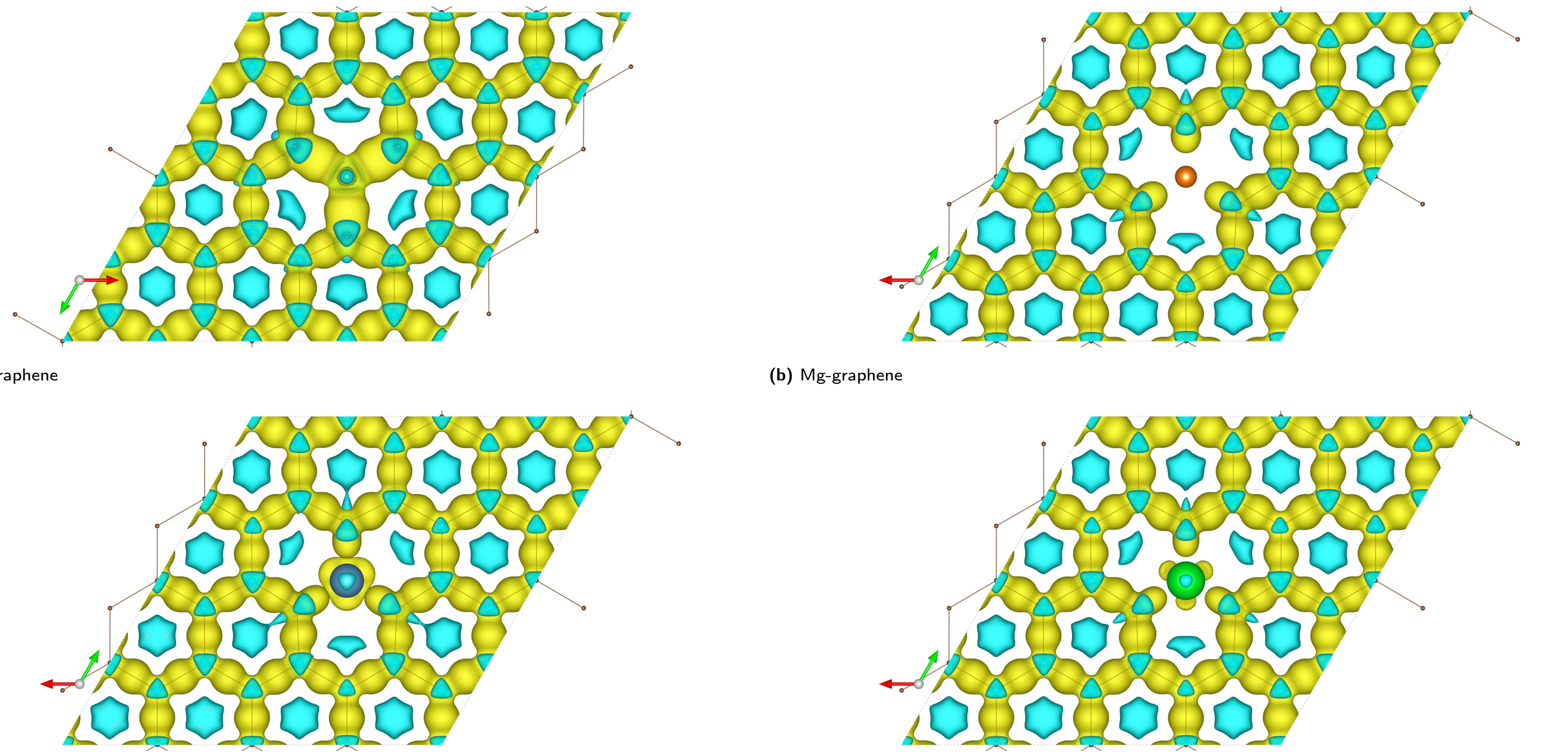

(a) Be-graphene

(b) Mg-graphene

(c) Ca-graphene

(d) Sr-graphene

Fig. S7 Supplementary charge difference plots for alkaline-earth doped graphenes. Bottom view is shown here to demonstrate charge-deficient areas on alkaline earth dopant. Yellow indicates regions which have an increase in electrons (negatively charged areas) while cyan indicates regions which have a decrease in electrons (positively charged areas). Isosurface level = $0.013e^{-}/bohr^{3}$. Brown atoms are carbon atoms while the differently colored atom denotes the alkaline earth dopant.

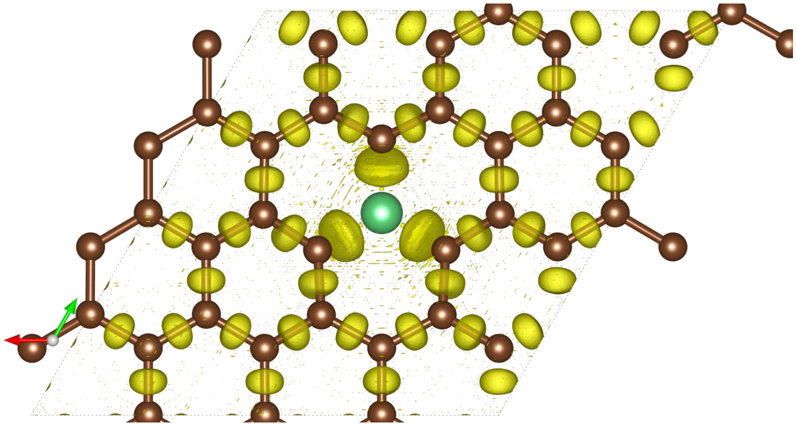

(a) Be-graphene

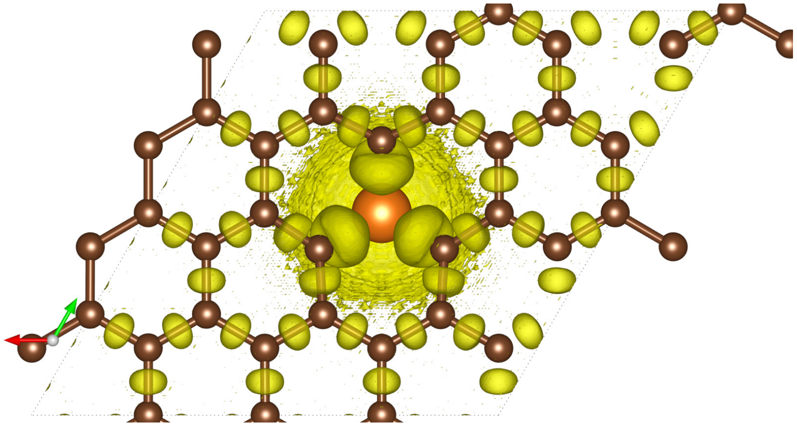

(b) Mg-graphene

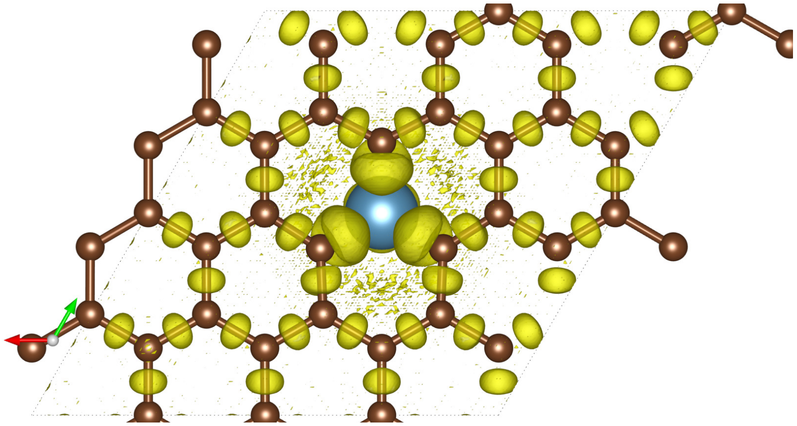

(c) Ca-graphene

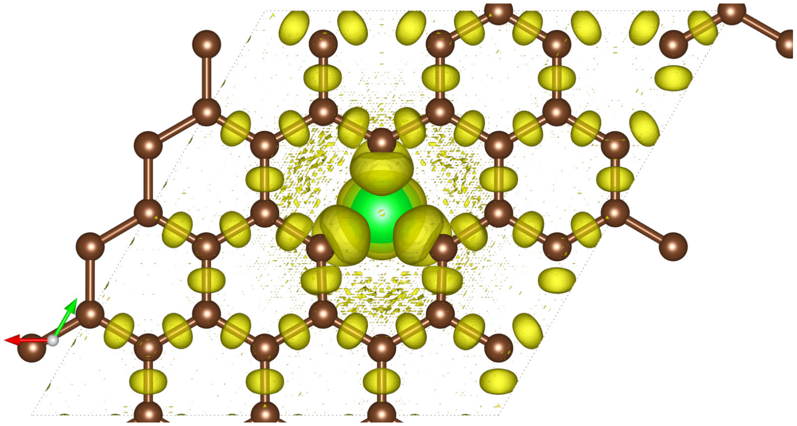

(d) Sr-graphene

Fig. S8 Supplementary electron localization function (ELF) plots for AE-graphenes. Bottom view is shown in these figures. Brown atoms are carbon atoms while the differently colored atom denotes the alkaline earth dopant. Isosurface level = 0.8 based on recommendation by Savin et al.[25]. Results at higher cutoff (1200 eV) available at Fig. H9†.

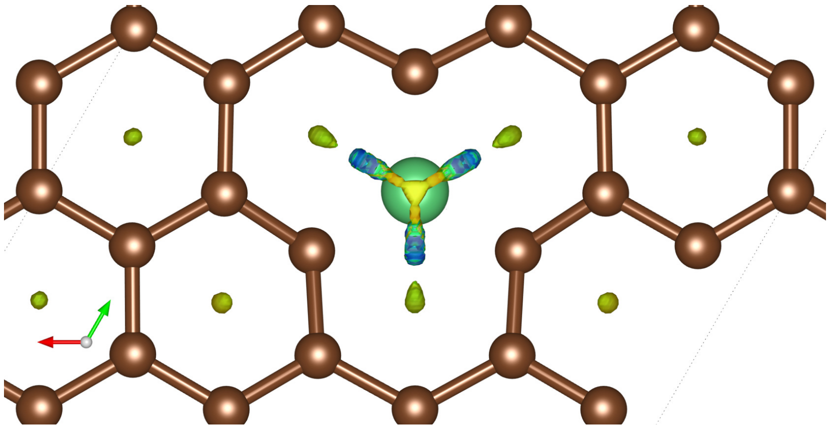

(a) Be-graphene

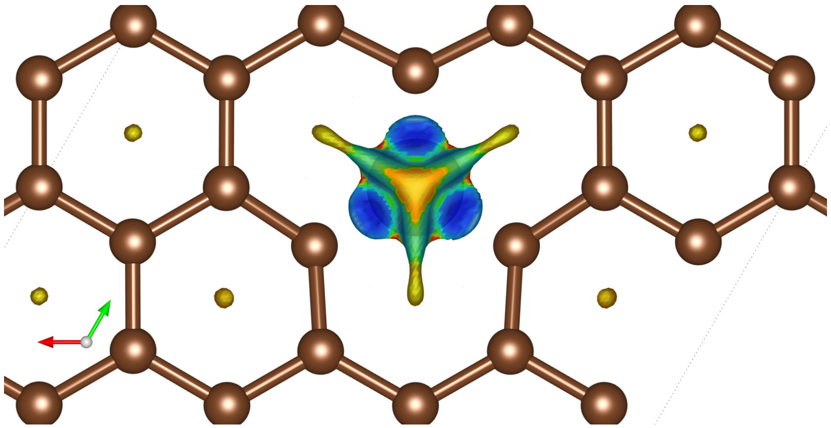

(b) Mg-graphene

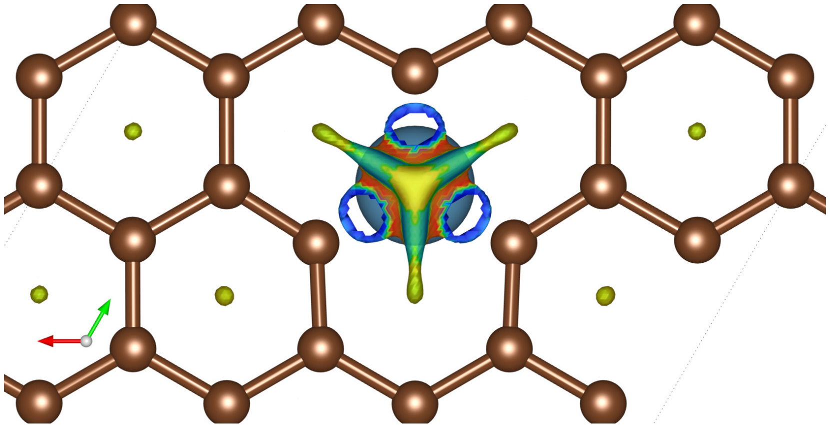

(c) Ca-graphene

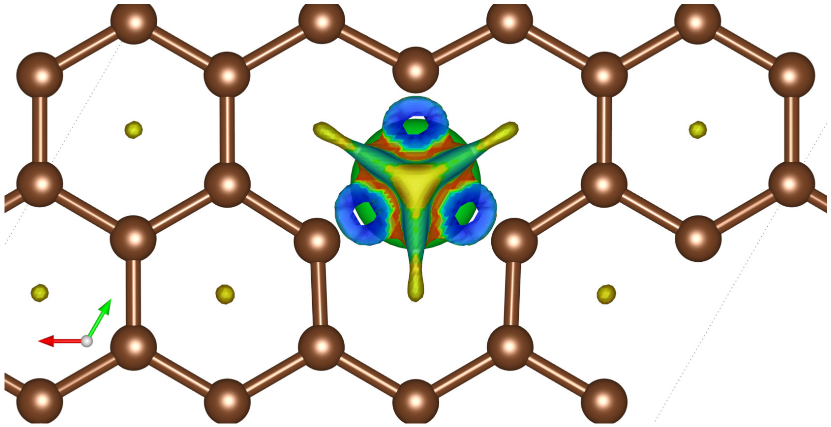

(d) Sr-graphene

Fig. S9 Supplementary reduced density gradient (RDG) plots for AE-graphenes. Spectrum from red to blue indicates repulsive to attractive interactions, respectively. Bottom view shown here to highlight zones of steric strain (red) under the alkaline earth atom dopants. Brown atoms are carbon atoms while the differently colored atom denotes the alkaline earth dopant. Isosurface level = 0.4.

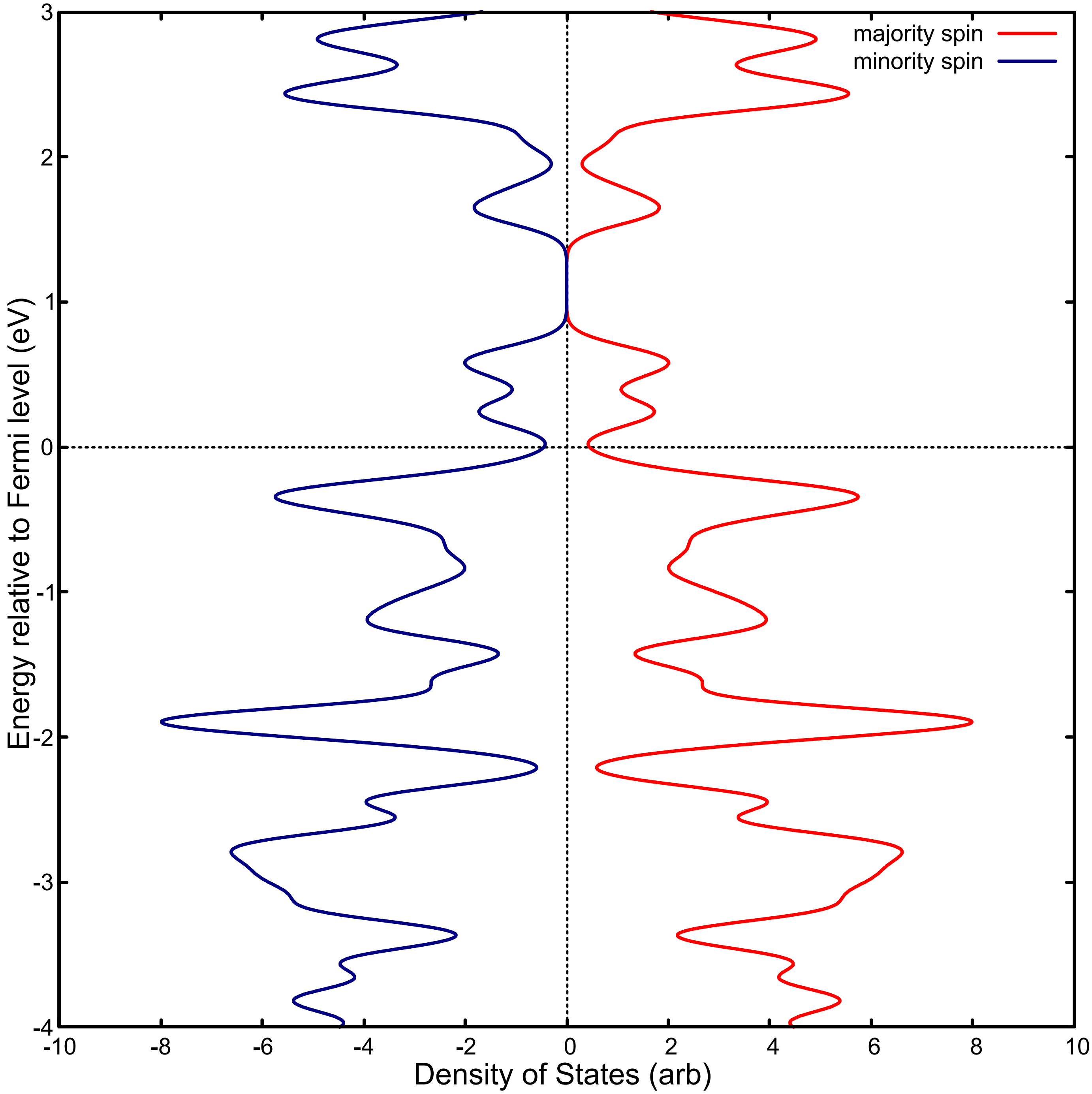

(a) Density of states (DOS)

Fig. S10 DOS for Be-graphene. For the DOS, red indicates majority spin (spin up) states and blue indicates minority spin (spin down) states.

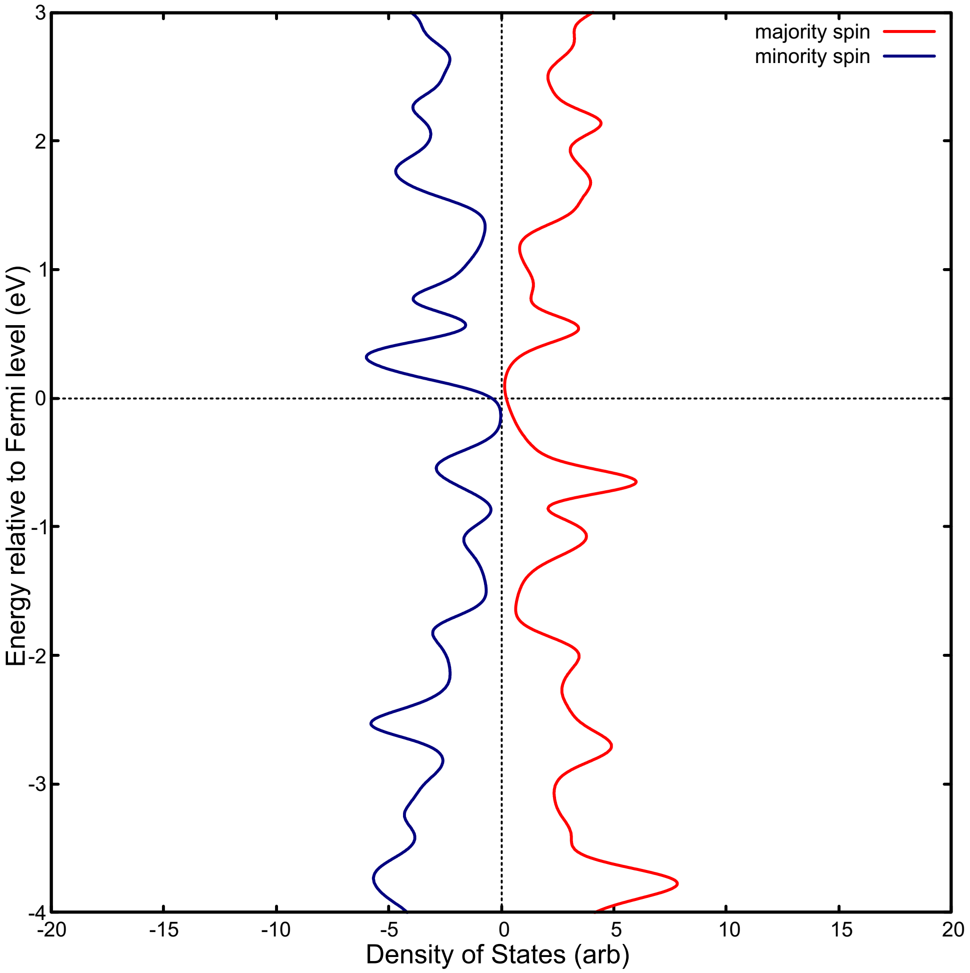

(a) Density of states (DOS)

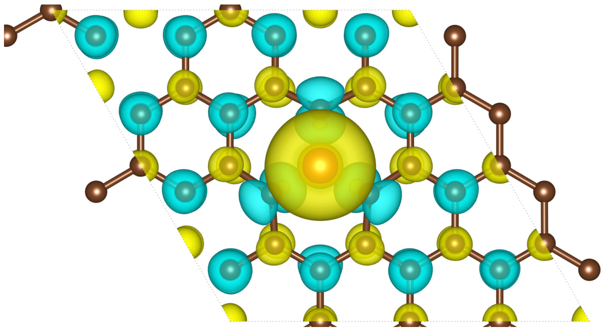

(b) majority spin VBM and CBM partial electron density

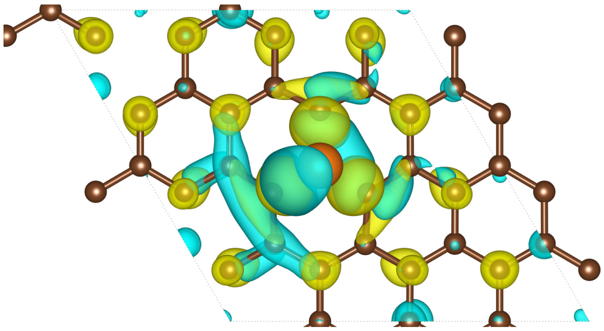

(c) minority spin VBM and CBM partial electron density

Fig. S11 DOS and VBM and CBM partial charge densities for Mg-graphene. For the DOS, red indicates majority spin (spin-up) states and blue indicates minority spin (spin down) states. For the VBM and CBM partial charge densities, yellow indicates regions contributing to the VBM, while cyan indicates regions contributing to the CBM. Brown atoms are carbon atoms while the differently colored atom denotes the alkaline earth dopant. Isosurface level for VBM and CBM partial charge densities = $0.0015 e^{-}/bohr^{3}$

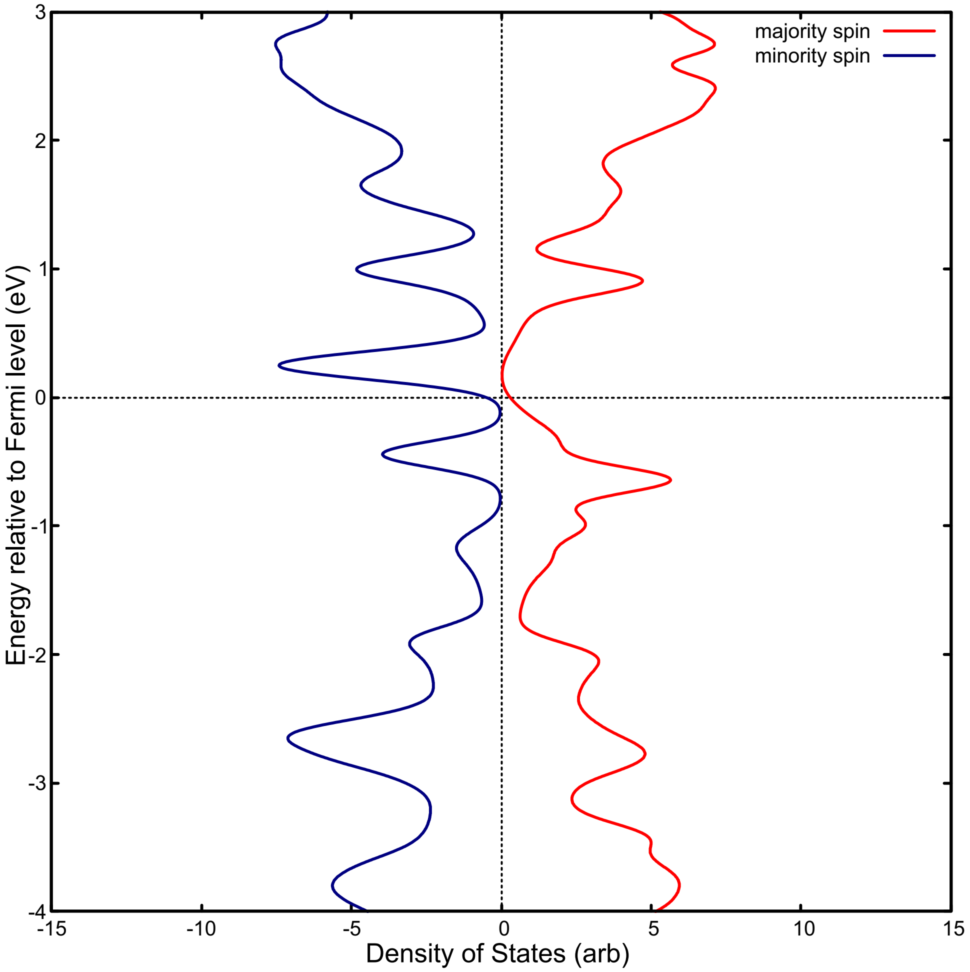

(a) Density of states (DOS)

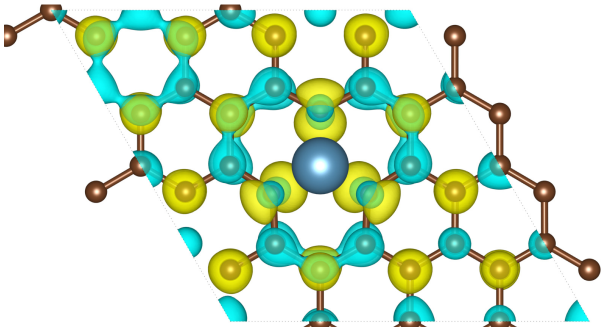

(b) majority spin VBM and CBM partial electron density

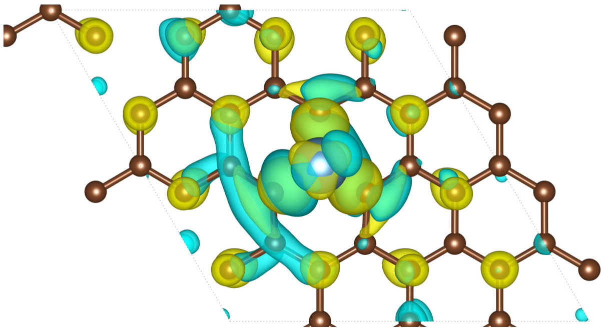

(c) minority spin VBM and CBM partial electron density

Fig. S12 DOS and VBM and CBM partial charge densities for Ca-graphene. For the DOS, red indicates majority spin (spin-up) states and blue indicates minority spin (spin down) states. For the VBM and CBM partial charge densities, yellow indicates regions contributing to the VBM, while cyan indicates regions contributing to the CBM. Brown atoms are carbon atoms while the differently colored atom denotes the alkaline earth dopant. Isosurface level for VBM and CBM partial charge densities = $0.0015e^{-}/bohr^{3}$

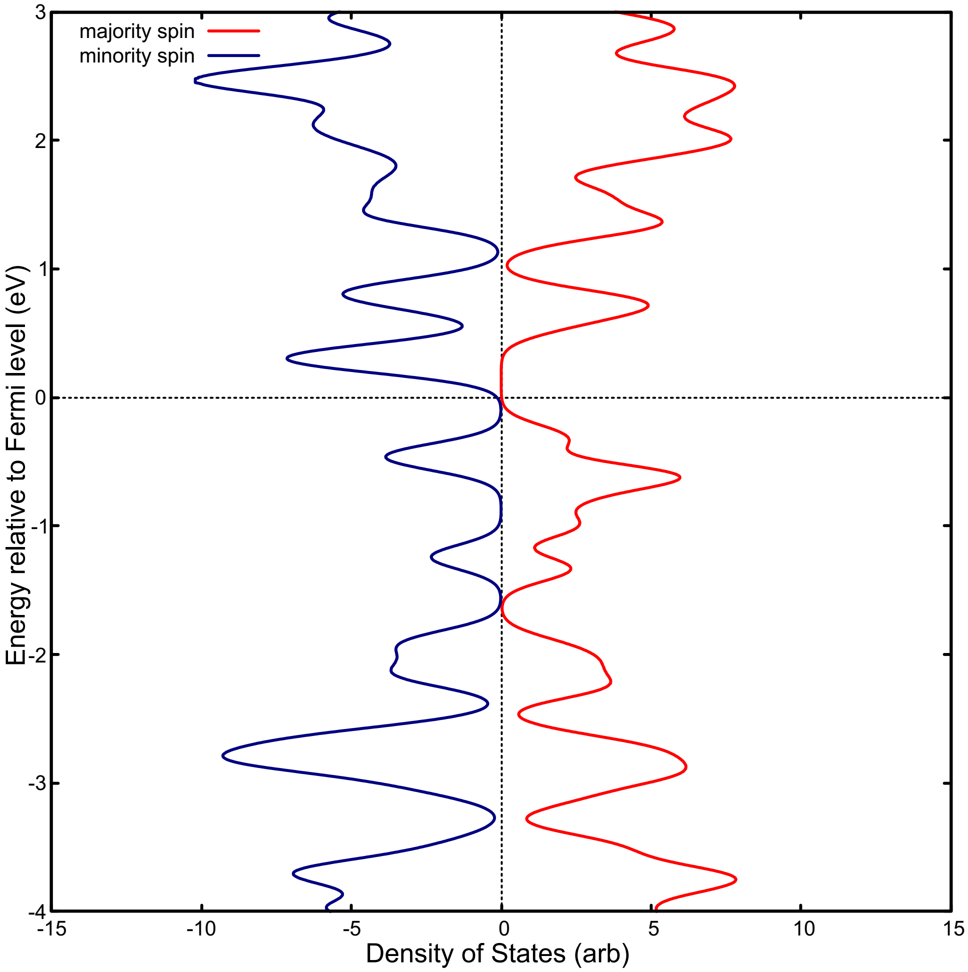

(a) Density of states (DOS)

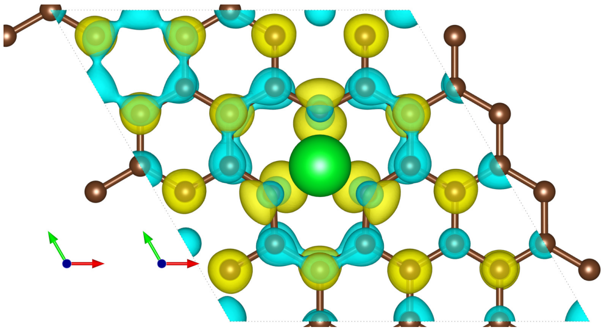

(b) majority spin VBM and CBM partial electron density

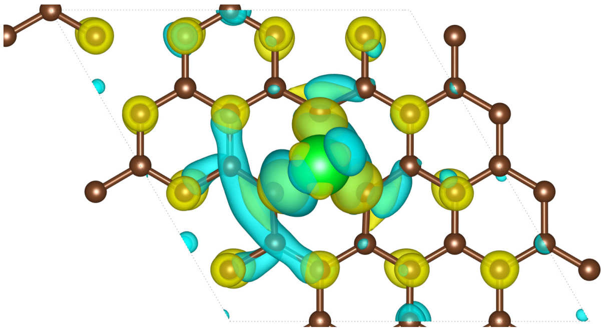

(c) minority spin VBM and CBM partial electron density

Fig. S13 DOS and VBM and CBM partial charge densities for Sr-graphene. For the DOS, red indicates majority spin (spin-up) states and blue indicates minority spin (spin down) states. For the VBM and CBM partial charge densities, yellow indicates regions contributing to the VBM, while cyan indicates regions contributing to the CBM. Brown atoms are carbon atoms while the differently colored atom denotes the alkaline earth dopant. Isosurface level for VBM and CBM partial charge densities $= 0.0015 e^{-}/bohr^{3}$

Notes and references

25 A. Savin, R. Nesper, S. Wengert and T. F. Fässler, *Angewandte Chemie International Edition in English*, 1997, **36**, 1808–1832.
