# Supplementary material for: Alkaline earth atom doping-induced changes in the electronic and magnetic properties of graphene: a density functional theory study
Source: RSC Adv. 2021 Feb 3;11(11):6268–83. doi: 10.1039/d0ra08115a (PMC8694801; doi:10.1039/d0ra08115a)
Supplement: RA-011-D0RA08115A-s002 [file RA-011-D0RA08115A-s002.pdf]

Electronic Supplementary Material (ESI) for RSC Advances.
This journal is © The Royal Society of Chemistry 2021

Electronic Supplementary Information: Verificatory results at 1200 eV cutoff energy

List of Figures

H1 Bader charge analysis for AE-graphenes at 1200 eV energy cutoff . . . 3
H2 Bader charge analysis and structure for Be-graphene at 1200 eV energy cutoff . . . 4
H3 Bader charge analysis and structure for Mg-graphene at 1200 eV energy cutoff . . . 5
H4 Bader charge analysis and structure for Ca-graphene at 1200 eV energy cutoff . . . 6
H5 Bader charge analysis and structure for Sr-graphene at 1200 eV energy cutoff . . . 7
H6 Plane averaged electrostatic potential plots for AE-graphenes at 1200 eV energy cutoff . . . 8
H7 Charge difference plots for AE-graphenes at 1200 eV energy cutoff . . . 9
H8 Supplementary charge difference plots for AE-graphenes at 1200 eV energy cutoff . . . 10
H9 Electron localization function (ELF) plots for AE-graphenes at 1200 eV energy cutoff . . . 11
H10 Supplementary electron localization function (ELF) plots for AE-graphenes at 1200 eV energy cutoff . . . 12
H11 Reduced density gradient (RDG) plots for AE-graphenes at 1200 eV energy cutoff . . . 13
H12 Supplementary reduced density gradient (RDG) plots for AE-graphenes at 1200 eV energy cutoff . . . 14
H13 Spin-difference plots for AE-graphenes at 1200 eV energy cutoff. . . . 15
H14 Electronic properties and orbital hybridization for Be-graphene at 1200 eV energy cutoff . . . 17
H15 DOS for Be-graphene at 1200 eV energy cutoff. . . . 18
H16 Electronic properties and orbital hybridization for Mg-graphene at 1200 eV energy cutoff . . . 19
H17 DOS and VBM - CBM partial charge densities for Mg-graphene at 1200 eV energy cutoff . . . 20
H18 Electronic properties and orbital hybridization for Ca-graphene at 1200 eV energy cutoff . . . 21
H19 DOS and VBM - CBM partial charge densities for Ca-graphene at 1200 eV energy cutoff . . . 22
H20 Electronic properties and orbital hybridization for Sr-graphene at 1200 eV energy cutoff . . . 23
H21 DOS and VBM - CBM partial charge densities for Sr-graphene at 1200 eV energy cutoff . . . 24

List of Tables

H1 Energetics, bond lengths and net charges in AE-graphenes at 1200 eV energy cutoff. . . . 2
H2 Band structure information for AE-Graphenes . . . 16

Table H1 Energetics, bond lengths and net charges in AE-graphenes at 1200 eV energy cutoff.

System	E_{bind} (eV/atom)	E_{ads} (eV)	W_0 (eV)	AE···C distance (Å)	Dopant net charge (e^-)
Be-graphene (out-of-plane)	-7.549	-6.294	4.841	1.627	+1.616
Be-graphene[1 t] (out-of-plane)	-7.715	-	-	1.62	+2.0
Be-graphene[2 to] (out-of-plane)	-8.699	-	-	1.622	+2.0
Be-graphene[3 t] (out-of-plane)	-	-7.02	-	1.48	-0.793
Be-graphene[4 t] (in-plane)	-8.86	-	-	1.56	+2.0
Be-graphene[2 to] (in-plane)	-8.692	-	-	1.568	+2.0
Mg-graphene	-7.452	-1.728	3.261	2.113	+1.332
Mg-graphene[3 t]	-	-2.10	-	2.11	+0.751
Ca-graphene	-7.496	-3.135	2.073	2.316	+1.276
Ca-graphene[3 t]	-	-3.59	-	2.30	+1.499
Ca-graphene[5 x]	-	-	3.60	-	-
Sr-graphene	-7.485	-2.806	1.834	2.459	+1.295
Sr-graphene[3 t]	-	-4.19	-	2.60, 2.48[w]	+1.125
Graphene	-7.890	-15.743	4.257	1.426	-
Graphene[5–14 xr]	-	-	4.2-4.8	1.44	-
Graphene[15–24 tr]	-	-	4.23-4.66	1.42-1.44	-
Graphene[3t]	-	-	-	1.42	-
Graphene with monovacancy	-8.497	-	-	-	-

[t] theoretical result
[x] experimental result
[o] authors' previous work
[r] range provided from results by previous work
[w] two values reported since asymmetrical adsorption was predicted[3]

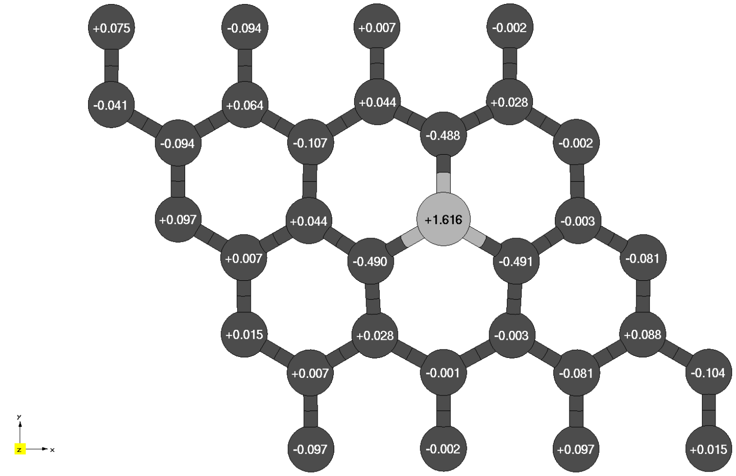

(a) Be-graphene

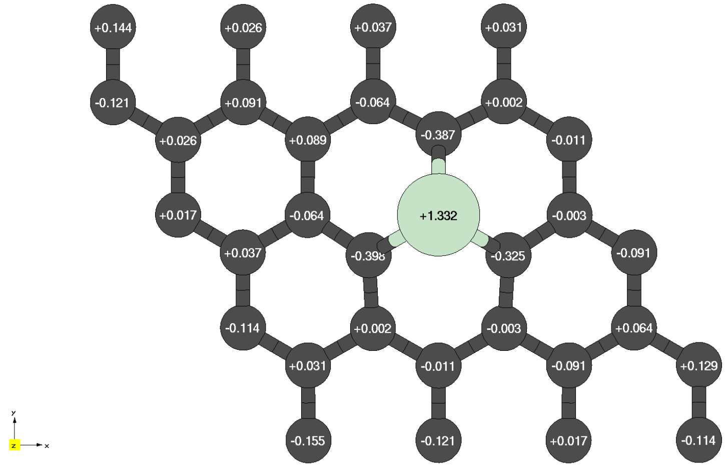

(b) Mg-graphene

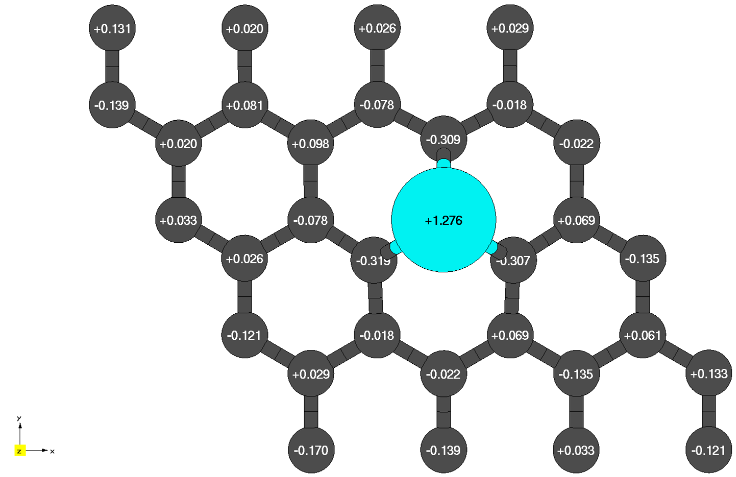

(c) Ca-graphene

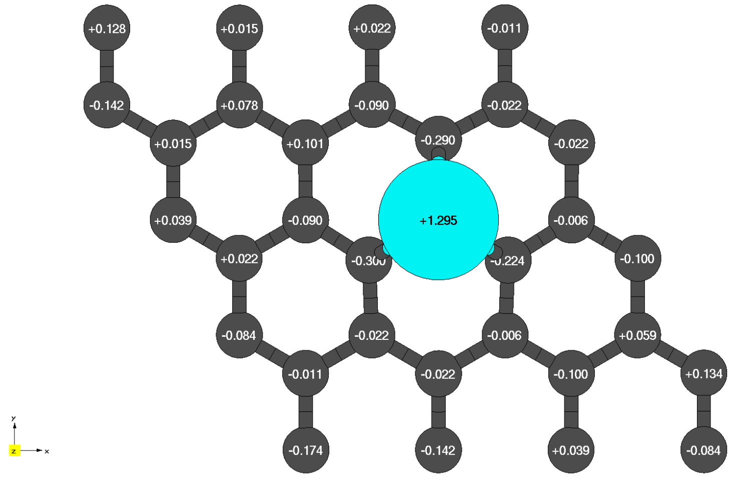

(d) Sr-graphene

Fig. H1 Bader charge analysis for AE-graphenes at 1200 eV energy cutoff. Net charges for each atom from Bader charge analysis are overlaid on the figure. Grey indicates carbon atoms while the differently colored atom indicates the alkaline earth dopant. Structures for AE-graphenes are detailed in Figs. H2, H3, H4, and H5†.

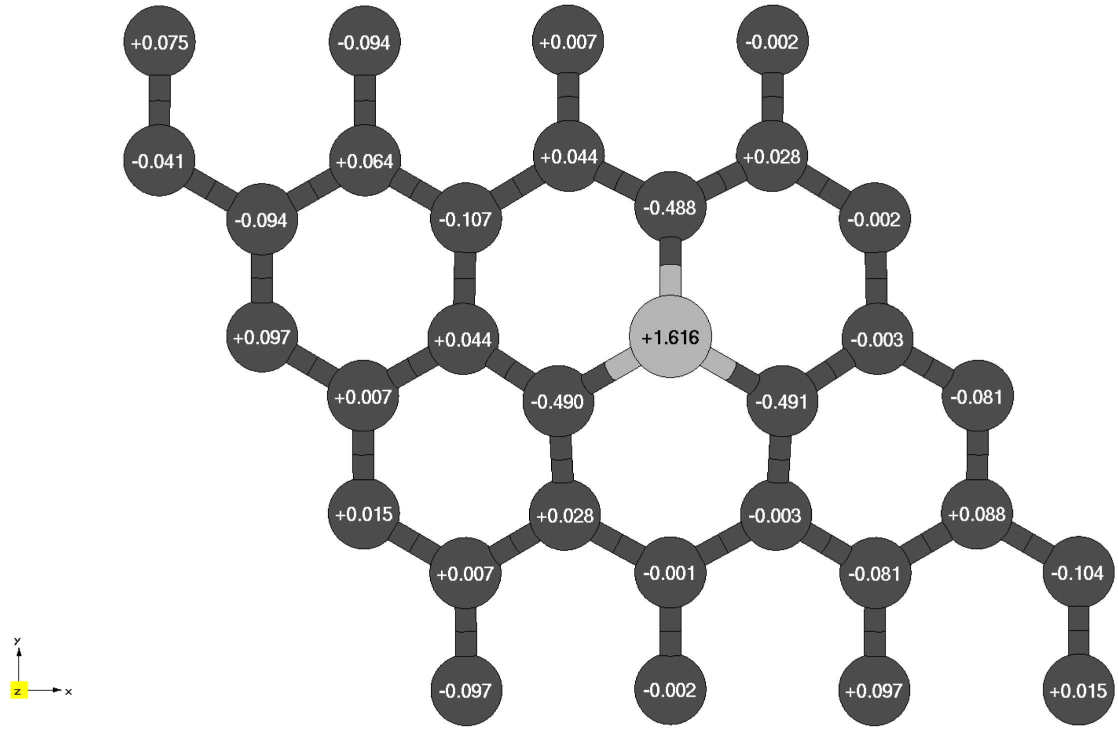

(a) top view

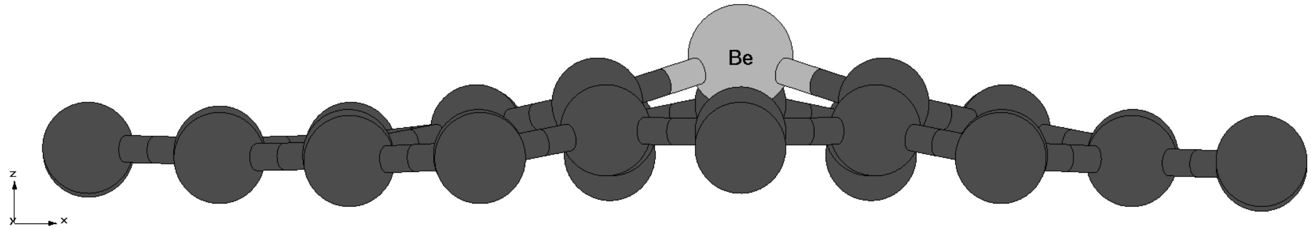

(b) side view

Fig. H2 Bader charge analysis and structure for Be-graphene at 1200 eV energy cutoff

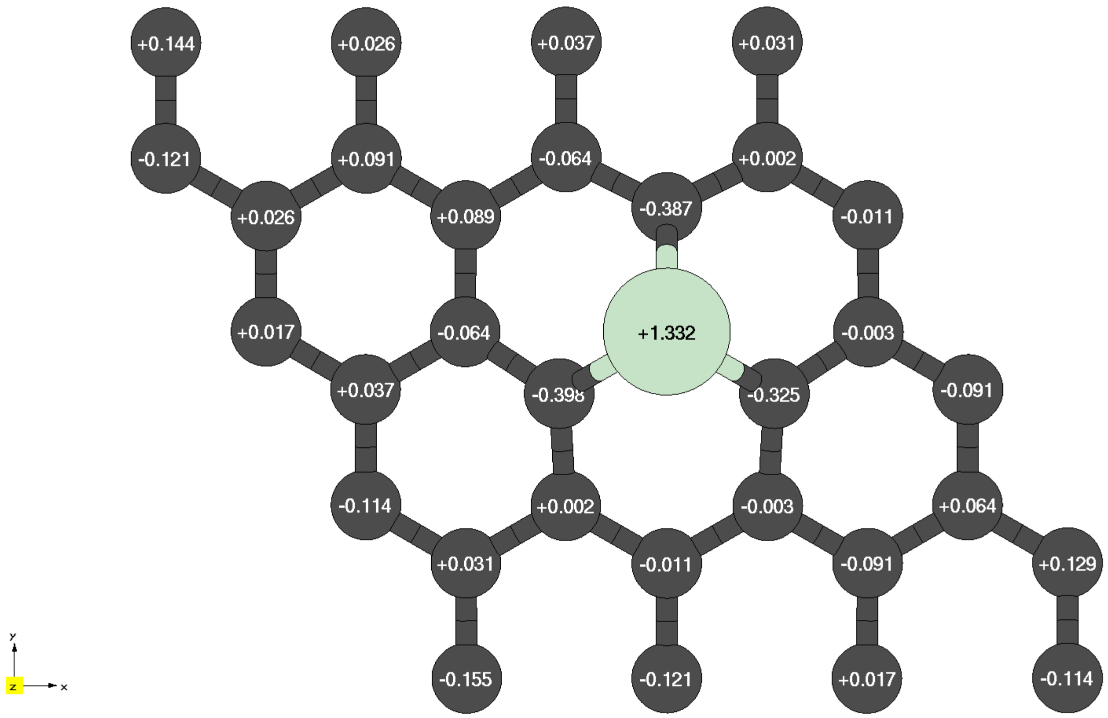

(a) top view

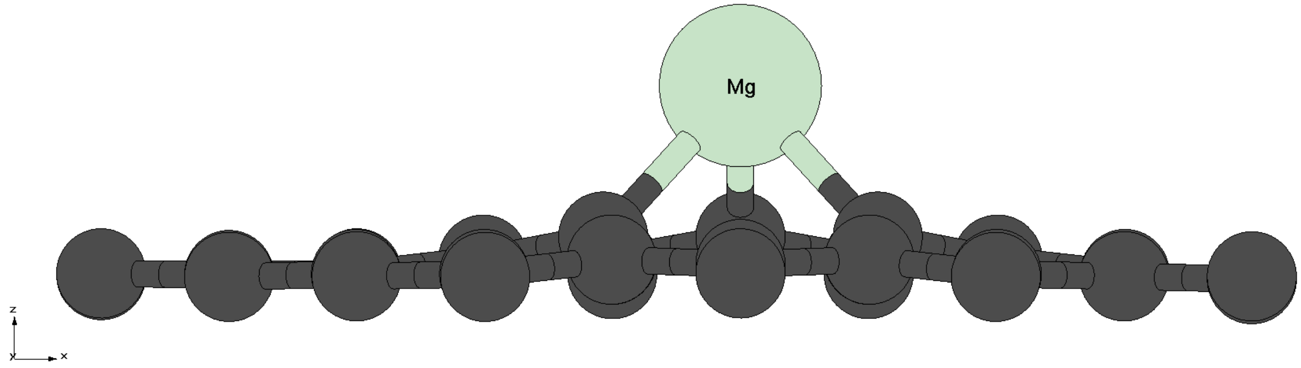

(b) side view

Fig. H3 Bader charge analysis and structure for Mg-graphene at 1200 eV energy cutoff

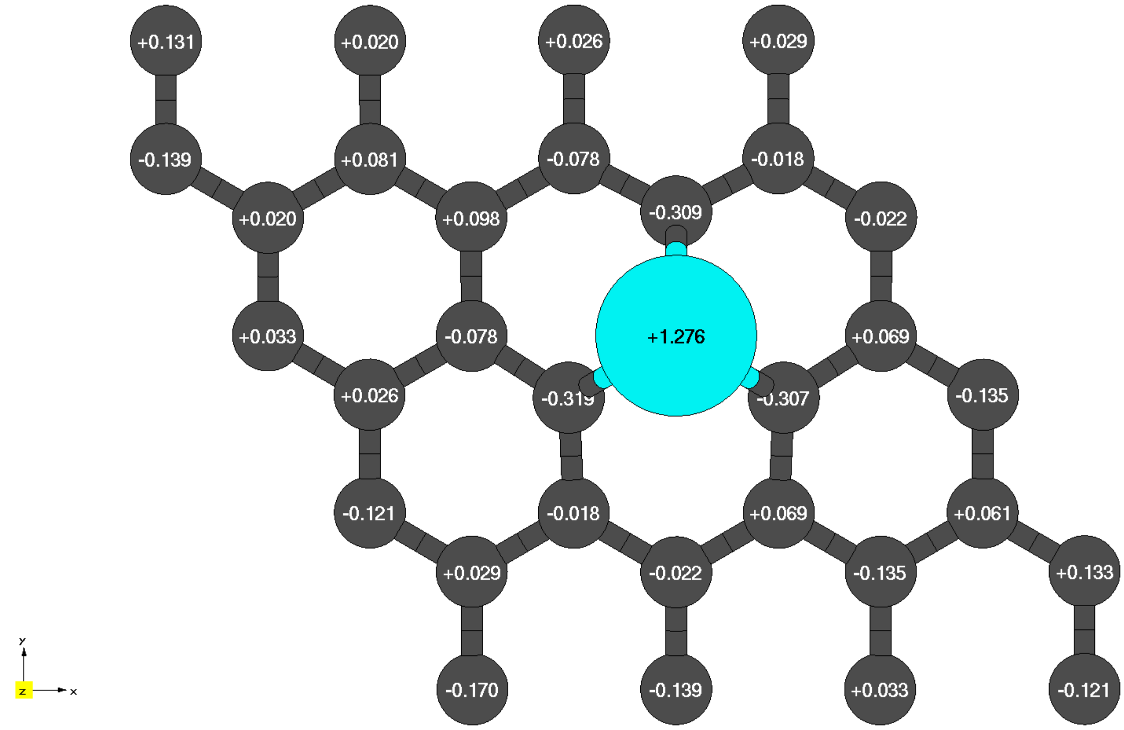

(a) top view

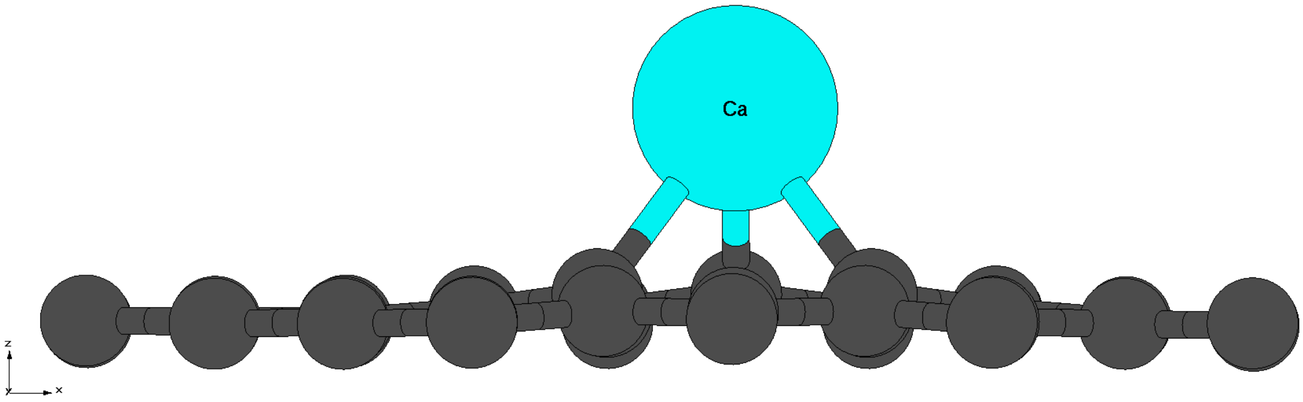

(b) side view

Fig. H4 Bader charge analysis and structure for Ca-graphene at 1200 eV energy cutoff

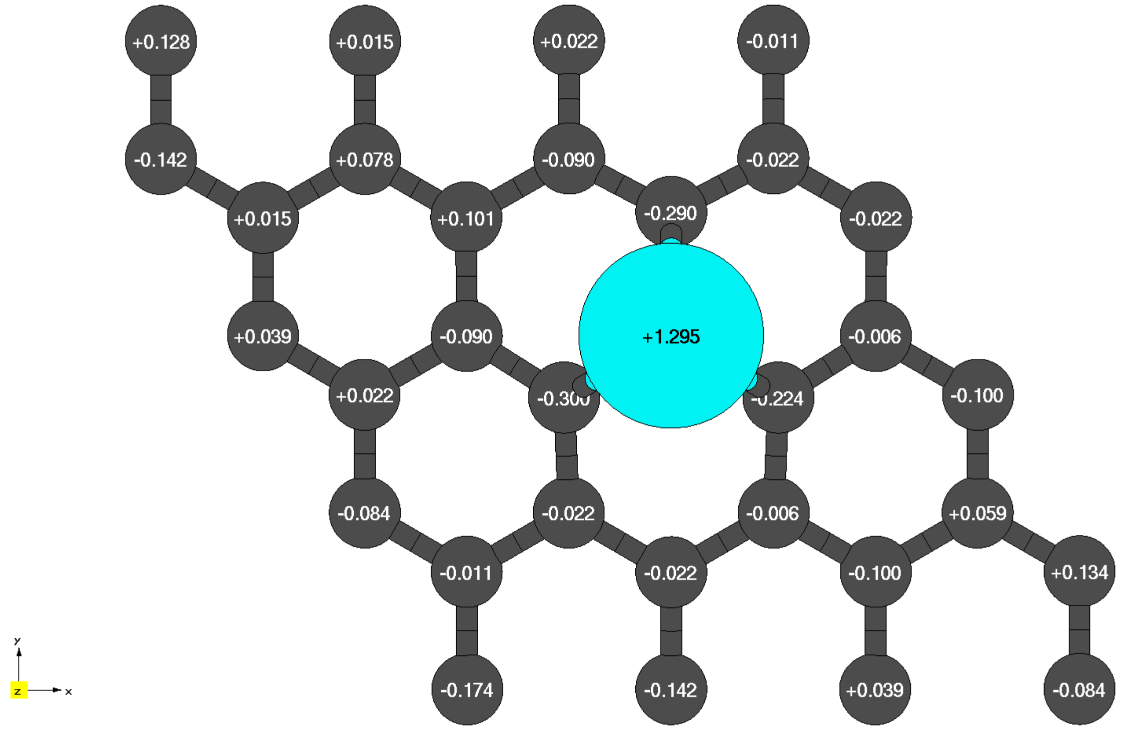

(a) top view

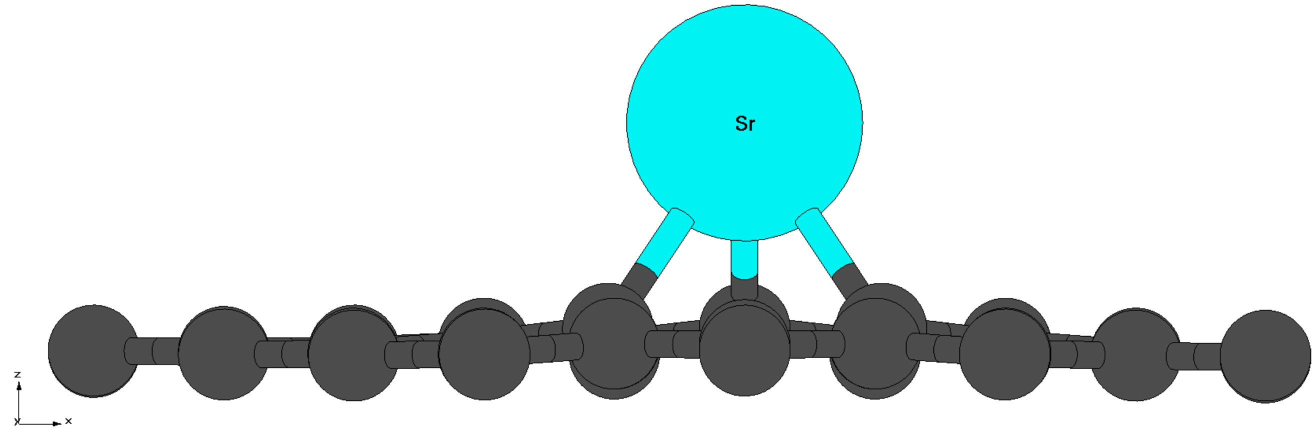

(b) side view

Fig. H5 Bader charge analysis and structure for Sr-graphene at 1200 eV energy cutoff

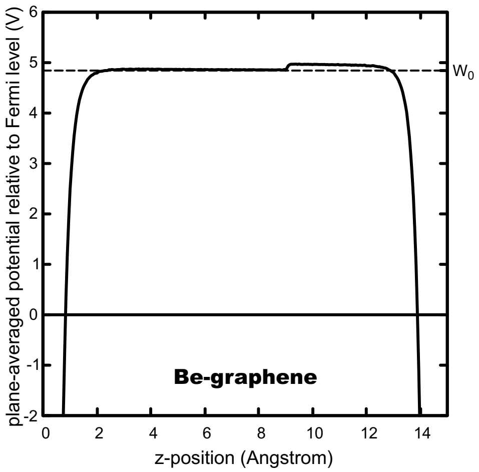

(a) Be-graphene. Workfunction (W_0) = 4.806 eV.
Workfunction with respect to pristine graphene = +0.541 eV

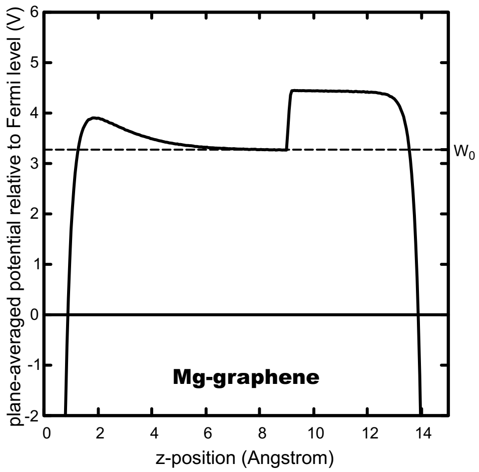

(b) Mg-graphene. Workfunction (W_0) = 3.136 eV.
Workfunction with respect to pristine graphene = -1.129 eV

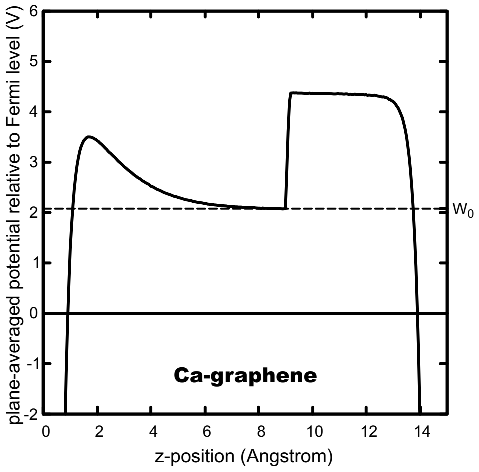

(c) Ca-graphene. Workfunction (W_0) = 2.053 eV.
Workfunction with respect to pristine graphene = -2.212 eV.

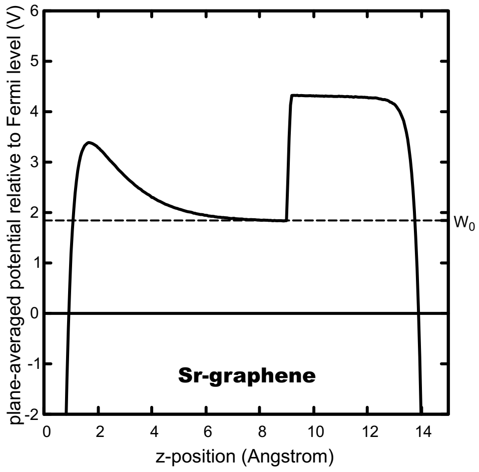

(d) Sr-graphene. Workfunction (W_0) = 1.815 eV.
Workfunction with respect to pristine graphene = -2.450 eV.

Fig. H6 Plane averaged electrostatic potential plots for AE-graphenes at 1200 eV energy cutoff. Dashed line indicates the vacuum level (E_{vac}) at the dopant-containing side of graphene. V = 0V for the plots is set at Fermi level (E_{Fermi}) for each AE-graphene. Dopant is oriented towards the positive z-axis direction.

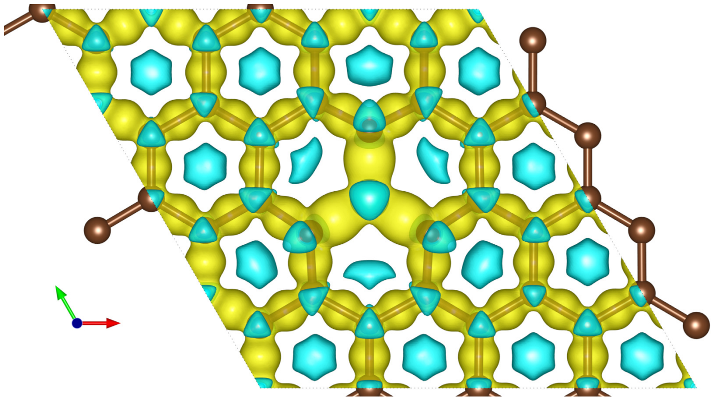

(a) Be-graphene

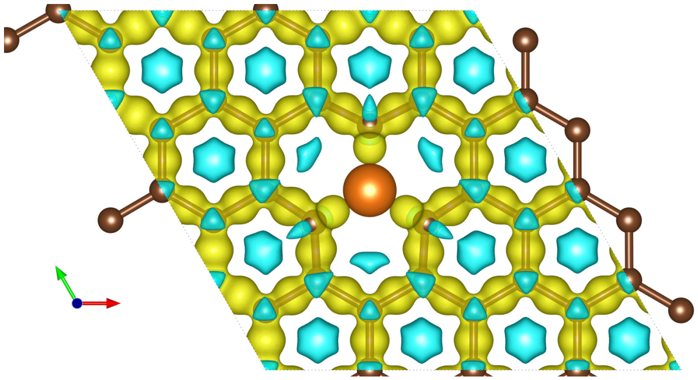

(b) Mg-graphene

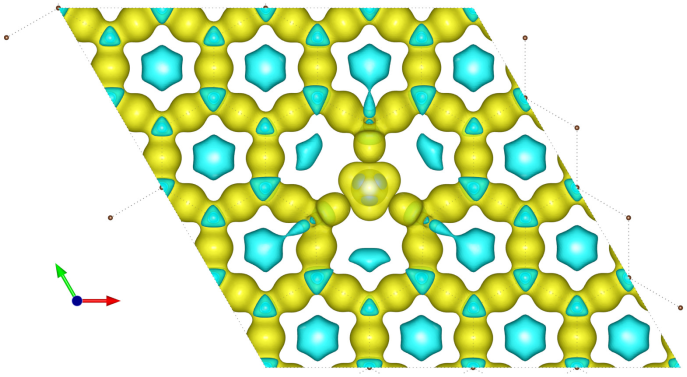

(c) Ca-graphene

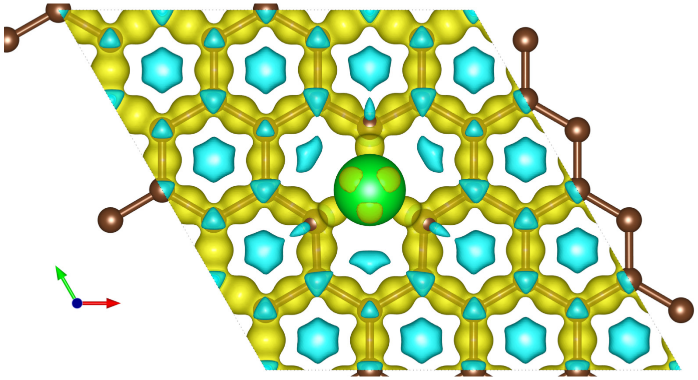

(d) Sr-graphene

Fig. H7 Charge difference plots for alkaline-earth doped graphenes at 1200 eV energy cutoff. Yellow indicates regions which have an increase in electrons (negatively charged areas) while cyan indicates regions which have a decrease in electrons (positively charged areas). Isosurface level $= 0.013e^{-}/bohr^{3}$. Brown atoms are carbon atoms while the differently colored atom denotes the alkaline earth dopant. Supplementary information at Fig. H8†

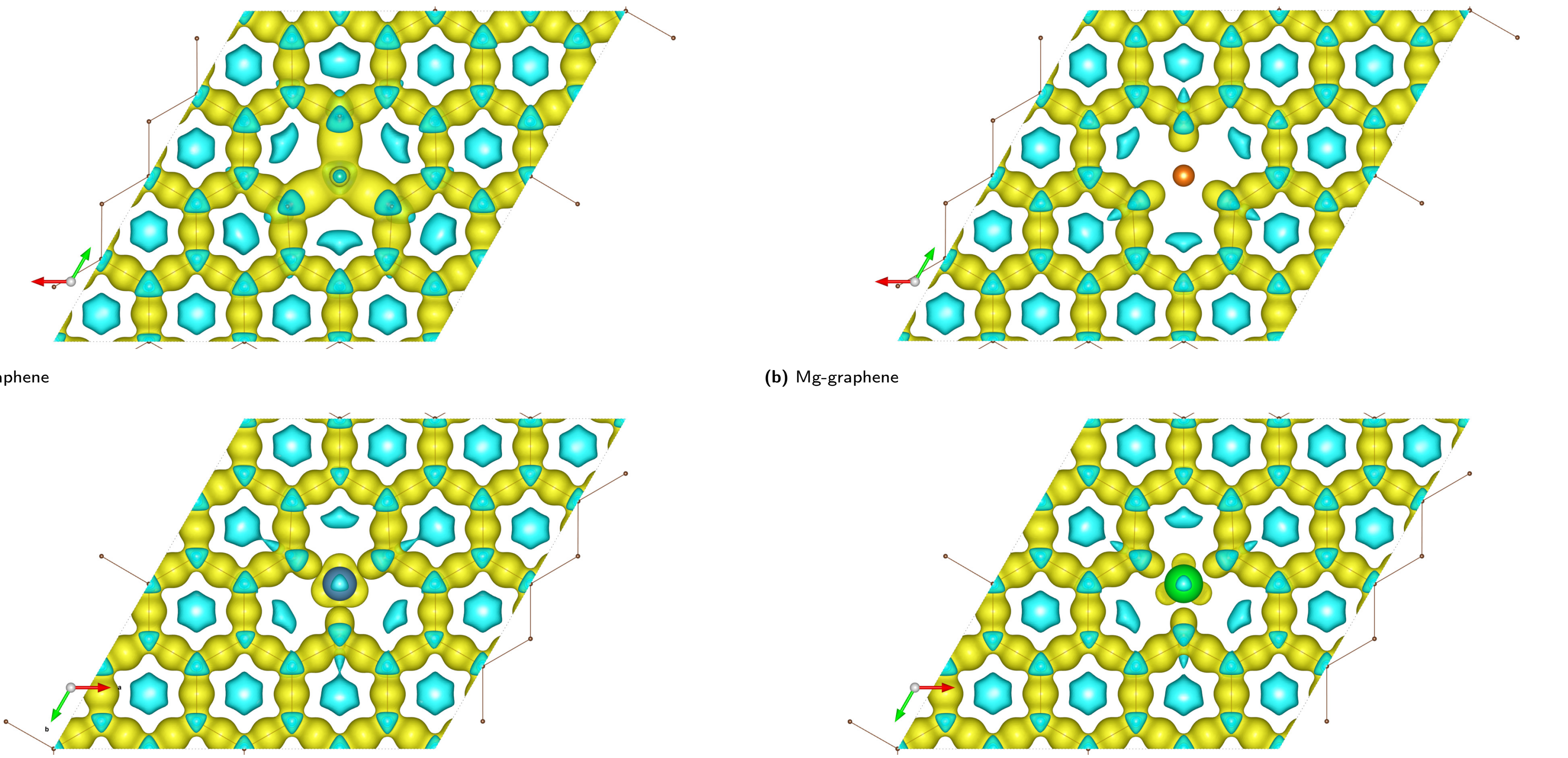

(a) Be-graphene

(b) Mg-graphene

(c) Ca-graphene

(d) Sr-graphene

Fig. H8 Supplementary charge difference plots for alkaline-earth doped graphenes at 1200 eV energy cutoff. Bottom view is shown here to demonstrate charge-deficient areas on alkaline earth dopant. Yellow indicates regions which have an increase in electrons (negatively charged areas) while cyan indicates regions which have a decrease in electrons (positively charged areas). Isosurface level = $0.013e^{-}/bohr^{3}$. Brown atoms are carbon atoms while the differently colored atom denotes the alkaline earth dopant.

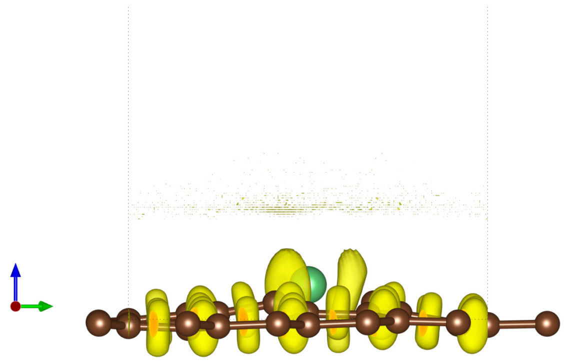

(a) Be-graphene

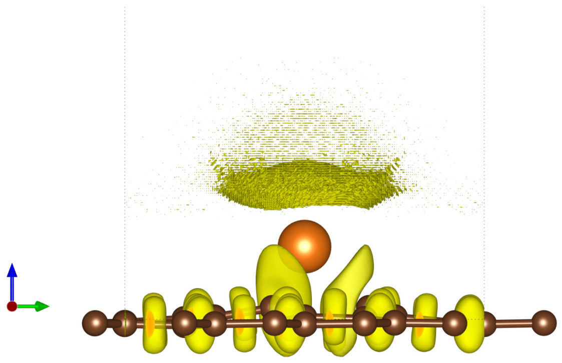

(b) Mg-graphene

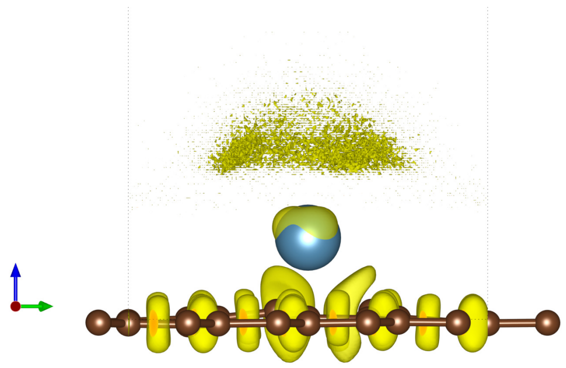

(c) Ca-graphene

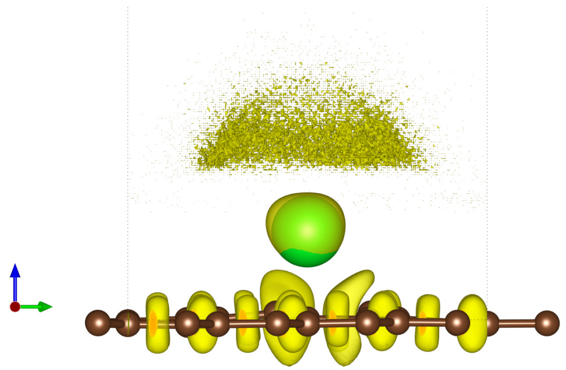

(d) Sr-graphene

Fig. H9 Electron localization function (ELF) plots for AE-graphenes at 1200 eV energy cutoff. Brown atoms are carbon atoms while the differently colored atom denotes the alkaline earth dopant. Isosurface level = 0.8 based on recommendation by Savin et al.[25]. Supplementary information at Fig. H10†.

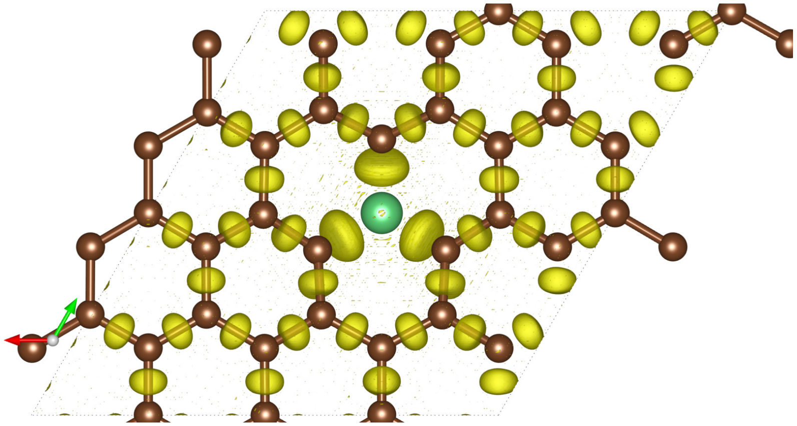

(a) Be-graphene

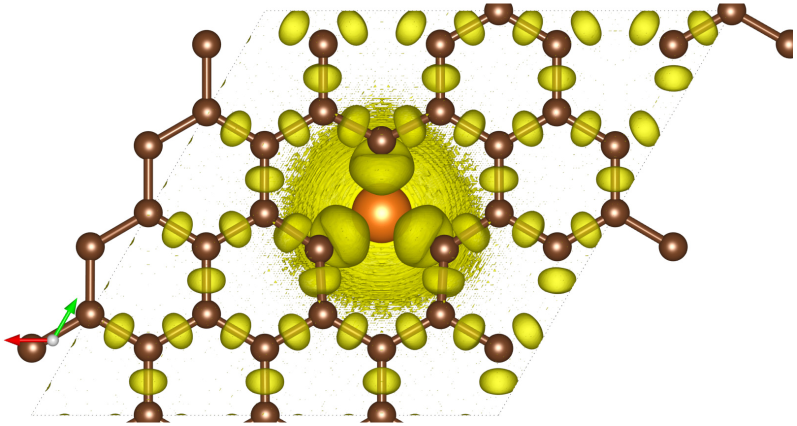

(b) Mg-graphene

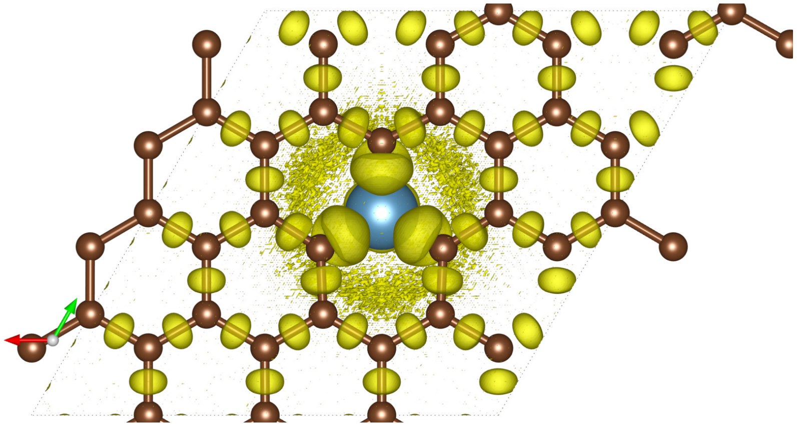

(c) Ca-graphene

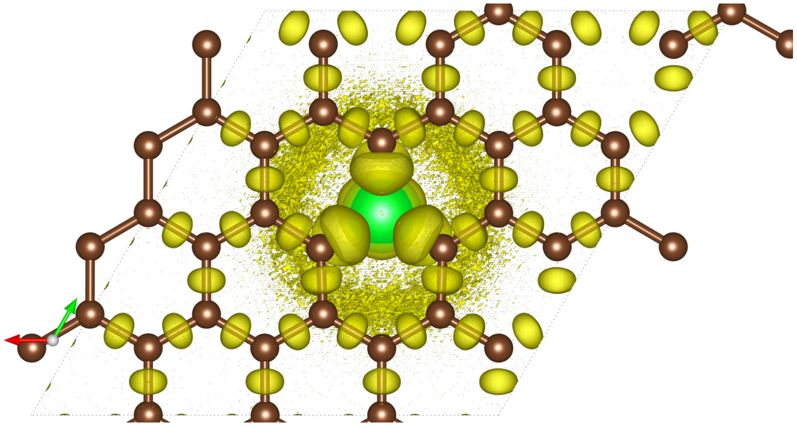

(d) Sr-graphene

Fig. H10 Supplementary electron localization function (ELF) plots for AE-graphenes at 1200 eV energy cutoff. Bottom view is shown in these figures. Brown atoms are carbon atoms while the differently colored atom denotes the alkaline earth dopant. Isosurface level = 0.8 based on recommendation by Savin et al.[25].

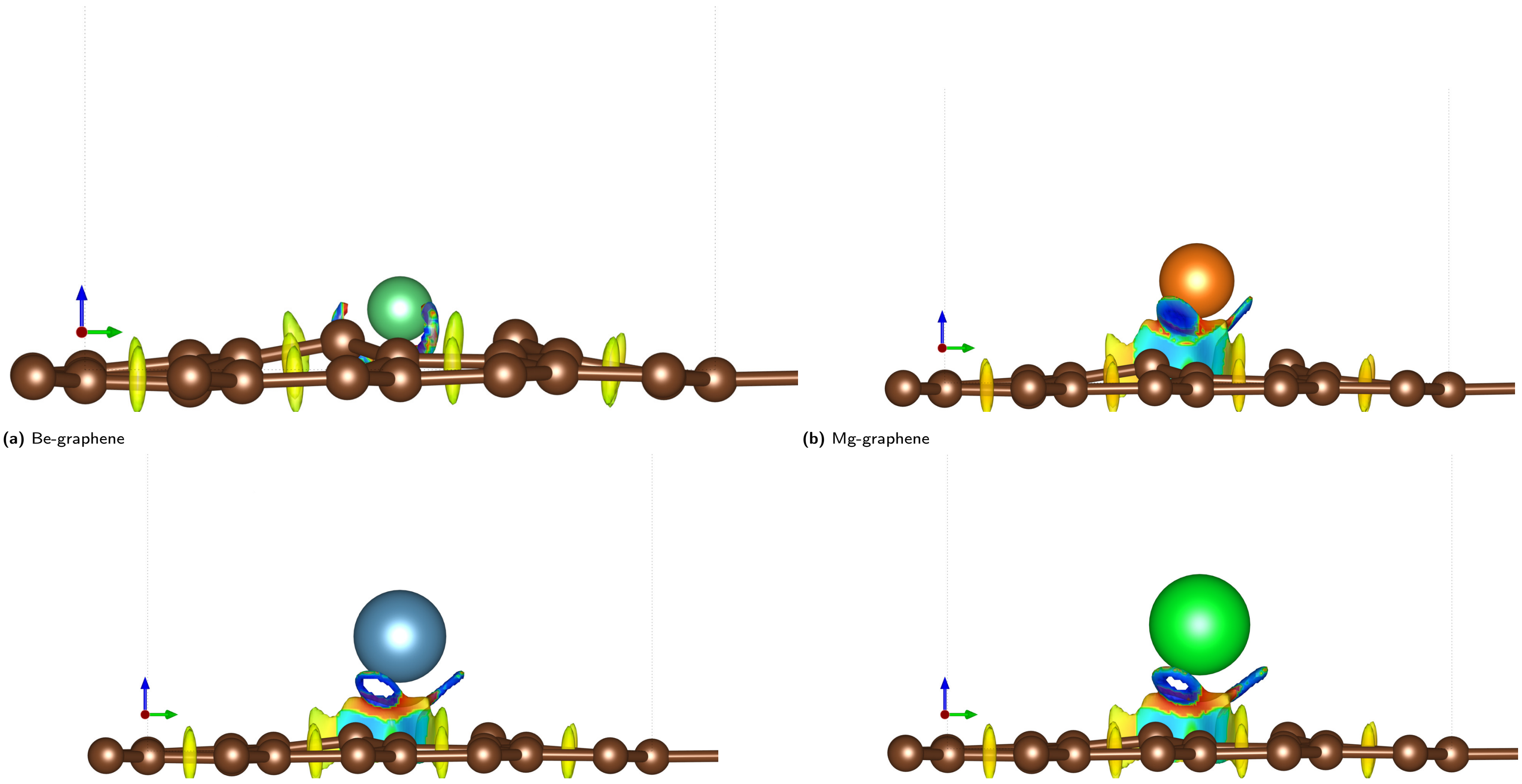

(a) Be-graphene

(b) Mg-graphene

(c) Ca-graphene

(d) Sr-graphene

Fig. H11 Reduced density gradient (RDG) plots for AE-graphenes at 1200 eV energy cutoff. Spectrum from red to blue indicates repulsive to attractive interactions, respectively. Brown atoms are carbon atoms while the differently colored atom denotes the alkaline earth dopant. Isosurface level = 0.4. Supplementary information available at Fig. H12†.

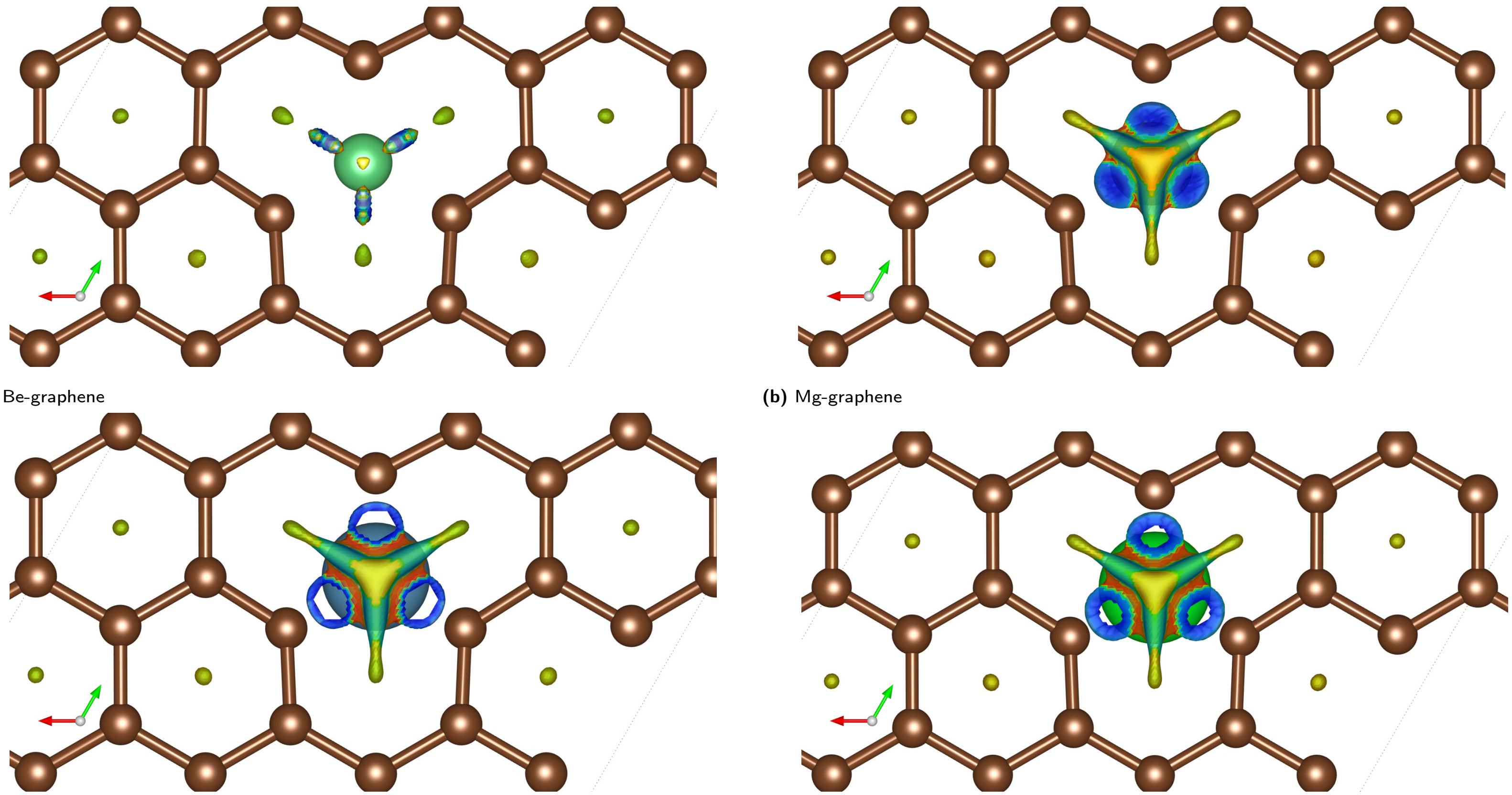

(a) Be-graphene

(b) Mg-graphene

(c) Ca-graphene

(d) Sr-graphene

Fig. H12 Supplementary reduced density gradient (RDG) plots for AE-graphenes at 1200 eV energy cutoff. Spectrum from red to blue indicates repulsive to attractive interactions, respectively. Bottom view shown here to highlight zones of steric strain (red) under the alkaline earth atom dopants. Brown atoms are carbon atoms while the differently colored atom denotes the alkaline earth dopant. Isosurface level = 0.4.

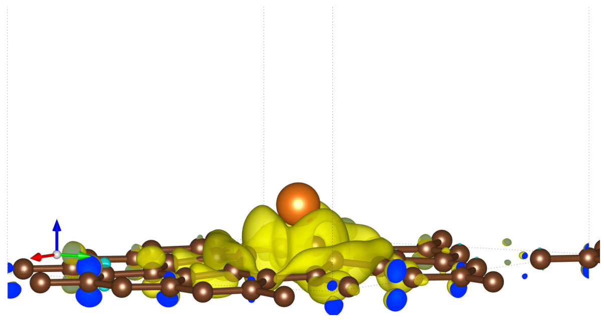

(a) Mg-graphene.
Total magnetization = 1.95 μ_B.
Absolute magnetization = 2.03 μ_B.

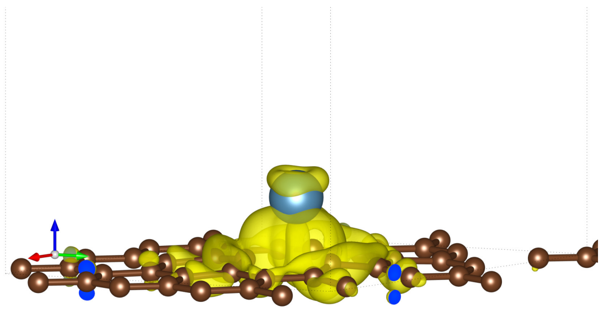

(b) Ca-graphene.
Total magnetization = 1.99 μ_B.
Absolute magnetization = 2.05 μ_B

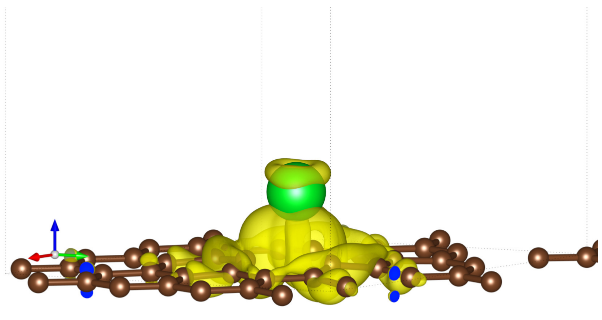

(c) Sr-graphene.
Total magnetization = 2.00 μ_B.
Absolute magnetization = 2.07 μ_B.

Fig. H13 Spin-difference plots for AE-graphenes at 1200 eV energy cutoff. Yellow signifies majority-spin dominant regions (spin-up) while cyan signifies minority-spin dominant regions (spin-down). Isosurface level = $0.002e^-/bohr^3$. Total and absolute magnetization values (μ_B) for each AE-graphenes are also indicated. Spin-difference plots for Be-graphene not included since total and absolute magnetization for Be-graphenes is zero. Brown atoms are carbon atoms while the differently colored atom denotes the alkaline earth dopant.

Table H2 Band structure information for AE-Graphenes. VBM and CBM levels are relative to the material's Fermi level at 1200 eV energy cutoff. VBM-CBM locations are indicated on the respective band structure plots (Fig. H16a,H18a and H20a).

Majority spin					
Dopant	VBM (eV)	CBM (eV)	VBM-CBM gap (eV)	gap type	VBM-CBM location
Be	-0.165	0.129	0.294	indirect, p-type	Γ-M
Mg	0.144	0.192	0.048	direct, p-type	K-K
Ca	0.038	0.358	0.320	direct, p-type	K-K
Sr	-0.027	0.280	0.253	direct, p-type	K-K
Minority spin					
Dopant	VBM (eV)	CBM (eV)	VBM-CBM gap (eV)	gap type	VBM-CBM location
Be	-0.165	0.129	0.294	indirect, p-type	Γ-M
Mg	-0.303	0.238	0.541	indirect, n-type	K-M
Ca	-0.375	0.196	0.571	indirect, n-type	K-Λ
Sr	-0.240	0.359	0.599	indirect, n-type	K-Λ

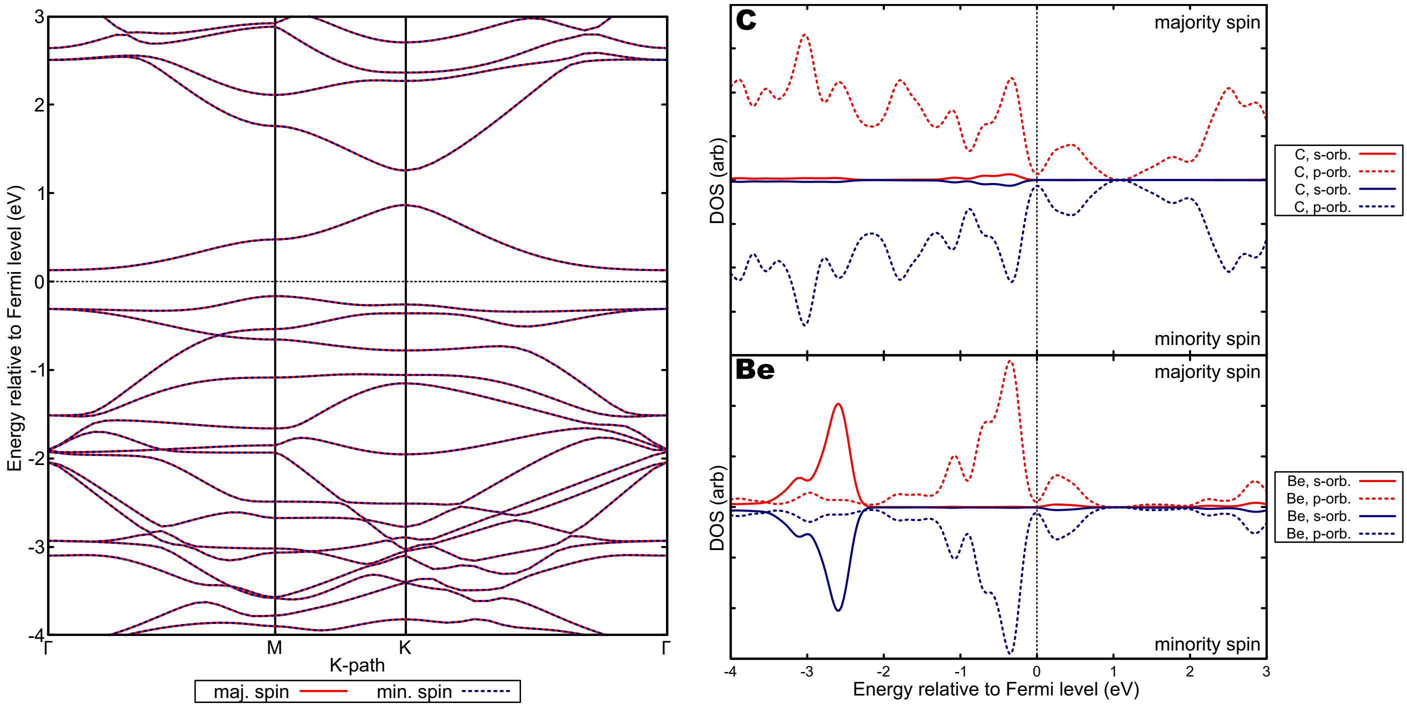

(a) Band structure

(b) projected DOS (pDOS) for Be-graphene

Fig. H14 Electronic properties and orbital hybridization for Be-graphene at 1200 eV energy cutoff. Red indicates majority spin (spin up) states and blue indicates minority spin (spin down) states. Supplementary information for electronic properties at Fig. H15 †.

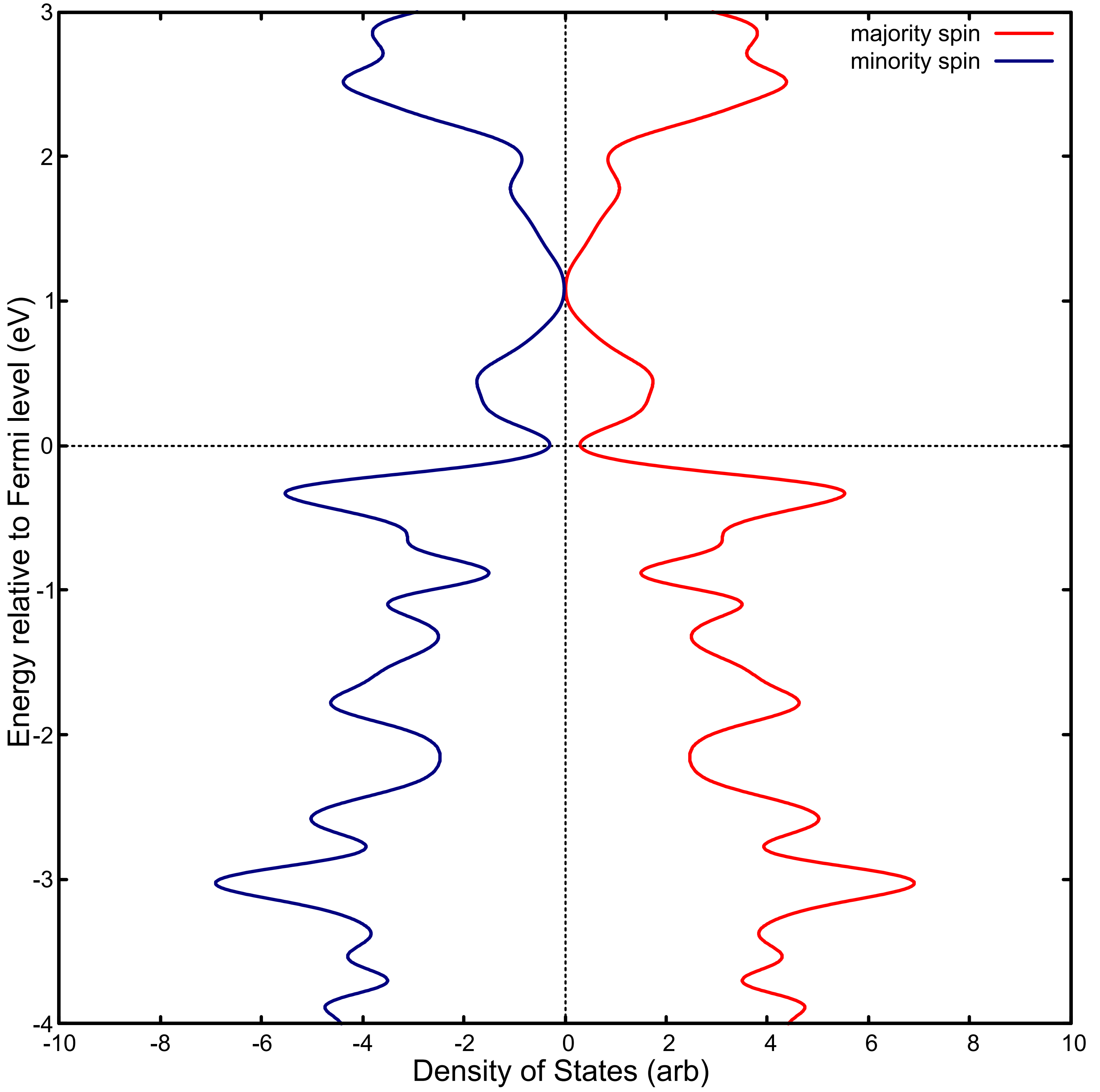

(a) Density of states (DOS)

Fig. H15 DOS for Be-graphene at 1200 eV energy cutoff. For the DOS, red indicates majority spin (spin up) states and blue indicates minority spin (spin down) states.

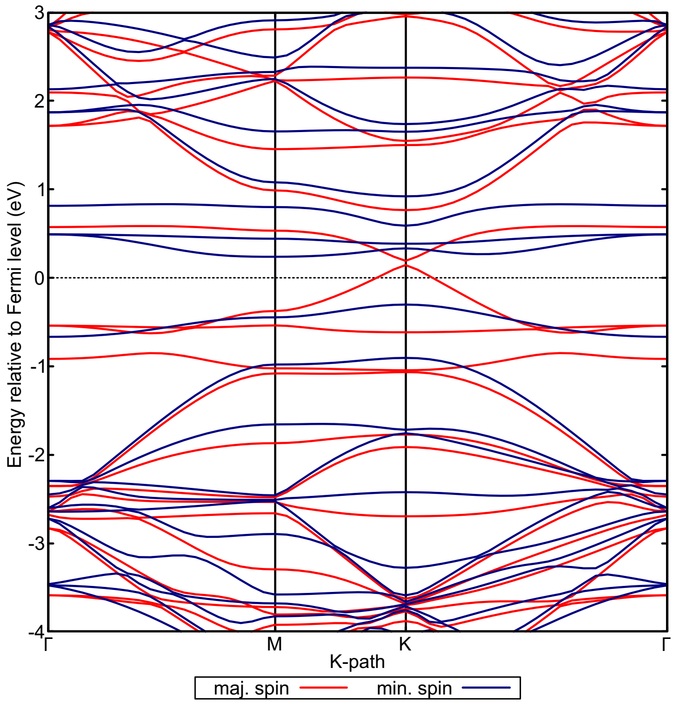

(a) Band structure

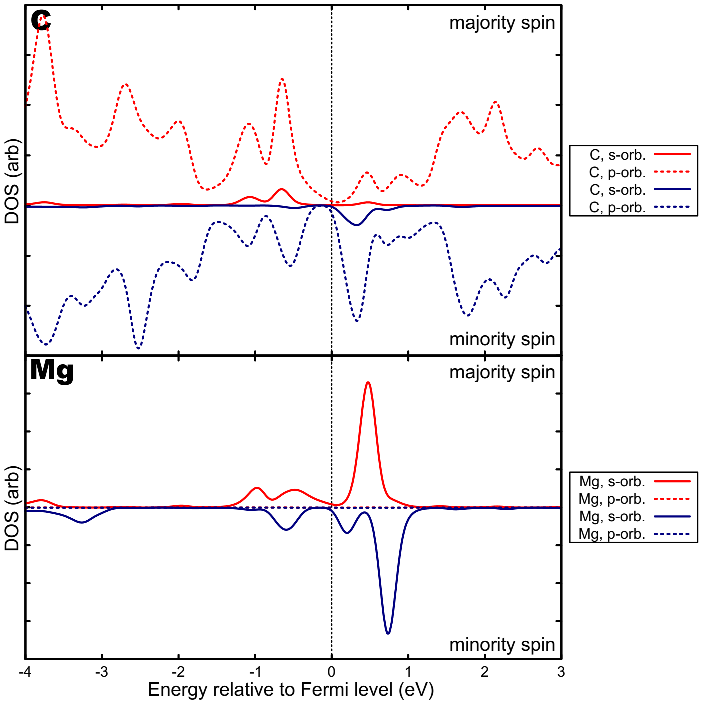

(b) projected DOS (pDOS) for Mg-graphene

Fig. H16 Electronic properties and orbital hybridization for Mg-graphene at 1200 eV energy cutoff. Red indicates majority spin (spin-up) states and blue indicates minority spin (spin down) states. Supplementary information for electronic properties at Fig. H17†.

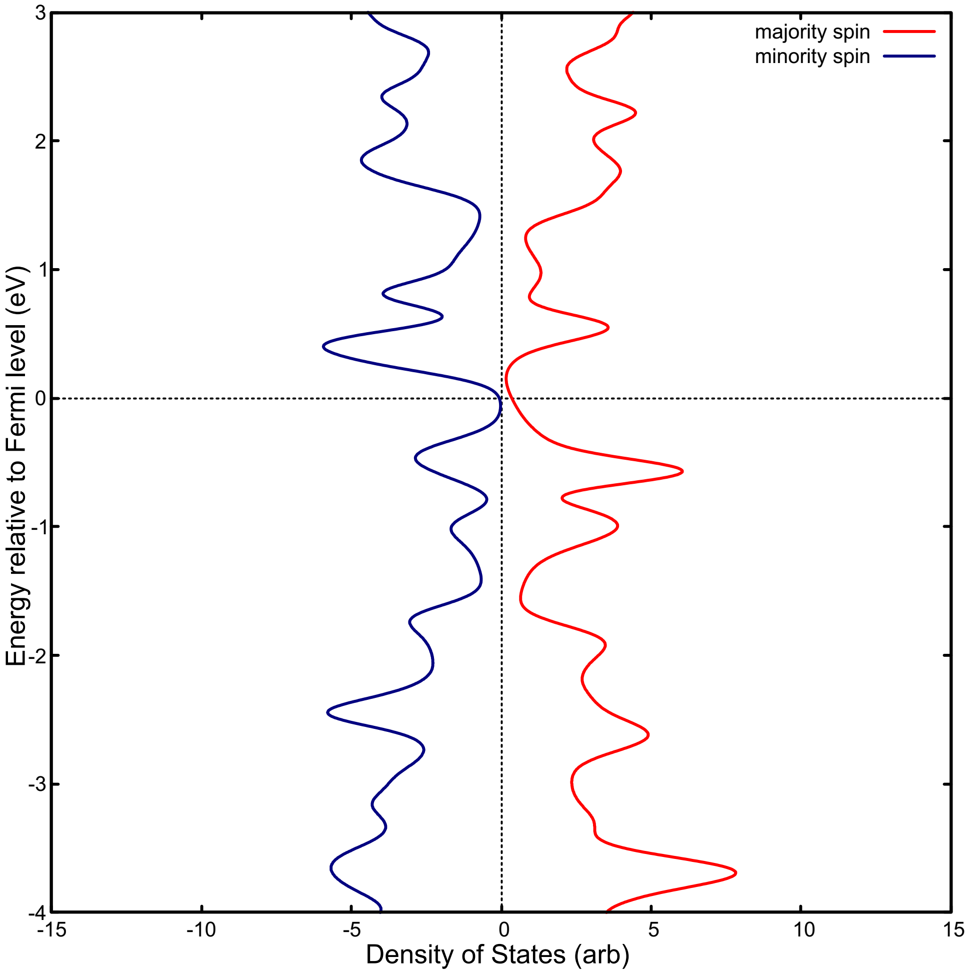

(a) Density of states (DOS)

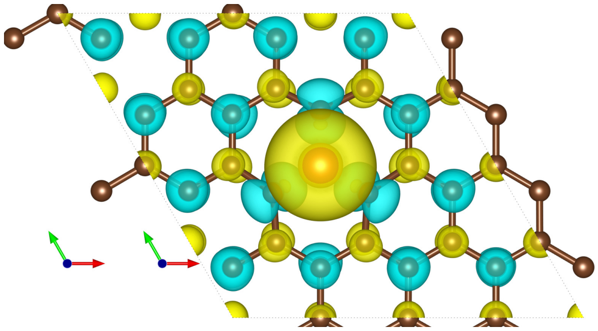

(b) majority spin VBM and CBM partial electron density

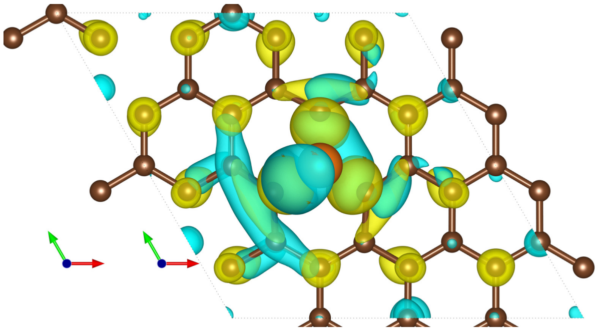

(c) minority spin VBM and CBM partial electron density

Fig. H17 DOS and VBM and CBM partial charge densities for Mg-graphene at 1200 eV energy cutoff. For the DOS, red indicates majority spin (spin-up) states and blue indicates minority spin (spin down) states. For the VBM and CBM partial charge densities, yellow indicates regions contributing to the VBM, while cyan indicates regions contributing to the CBM. Brown atoms are carbon atoms while the differently colored atom denotes the alkaline earth dopant. Isosurface level for VBM and CBM partial charge densities $= 0.0015 e^{-}/bohr^3$

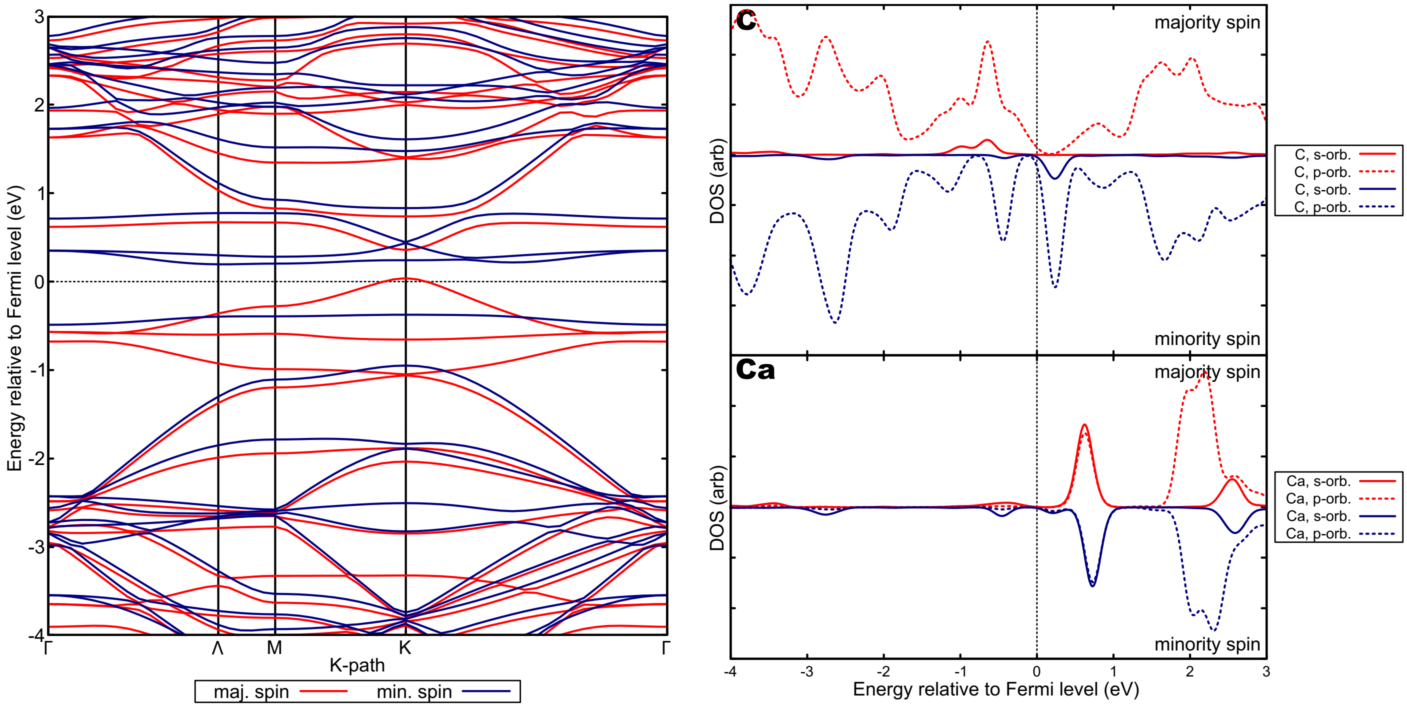

(a) Band structure

(b) projected DOS (pDOS) for Ca-graphene

Fig. H18 Electronic properties and orbital hybridization for Ca-graphene at 1200 eV energy cutoff. Red indicates majority spin (spin up) states and blue indicates minority spin (spin down) states. Supplementary information for electronic properties at Fig. H19†.

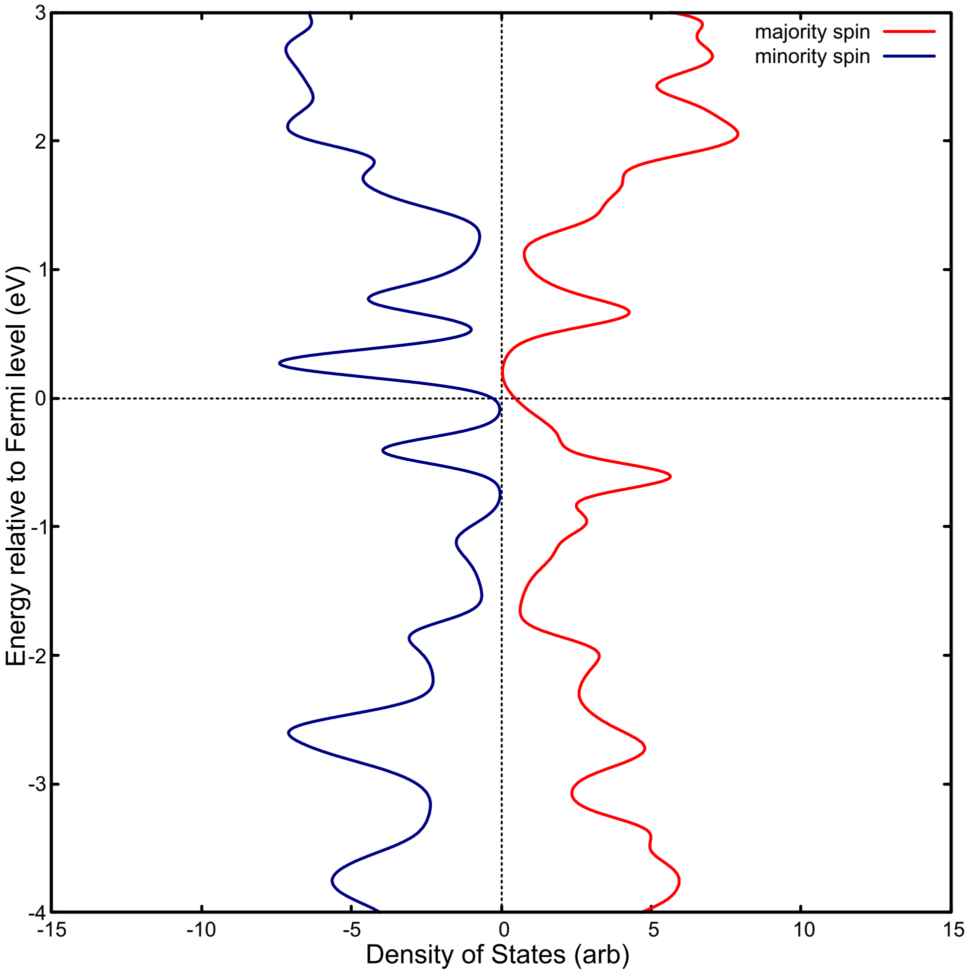

(a) Density of states (DOS)

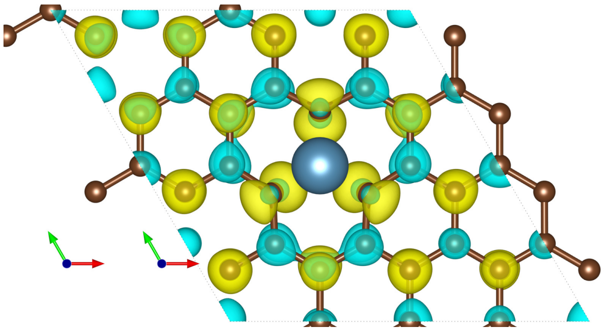

(b) majority spin VBM and CBM partial electron density

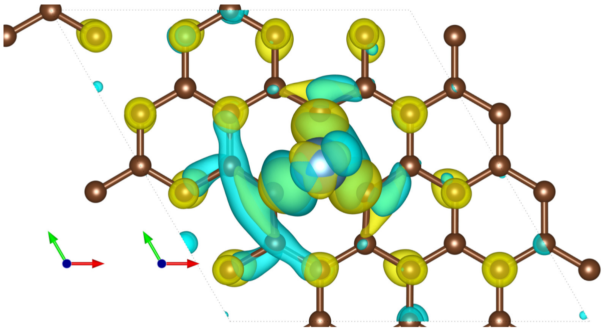

(c) minority spin VBM and CBM partial electron density

Fig. H19 DOS and VBM and CBM partial charge densities for Ca-graphene at 1200 eV energy cutoff. For the DOS, red indicates majority spin (spin-up) states and blue indicates minority spin (spin down) states. For the VBM and CBM partial charge densities, yellow indicates regions contributing to the VBM, while cyan indicates regions contributing to the CBM. Brown atoms are carbon atoms while the differently colored atom denotes the alkaline earth dopant. Isosurface level for VBM and CBM partial charge densities = $0.0015e^{-}/bohr^3$

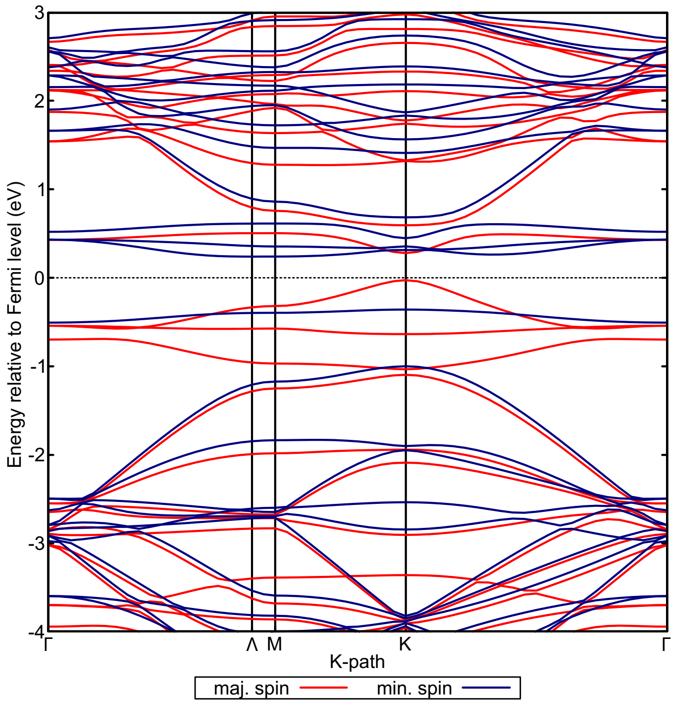

(a) Band structure

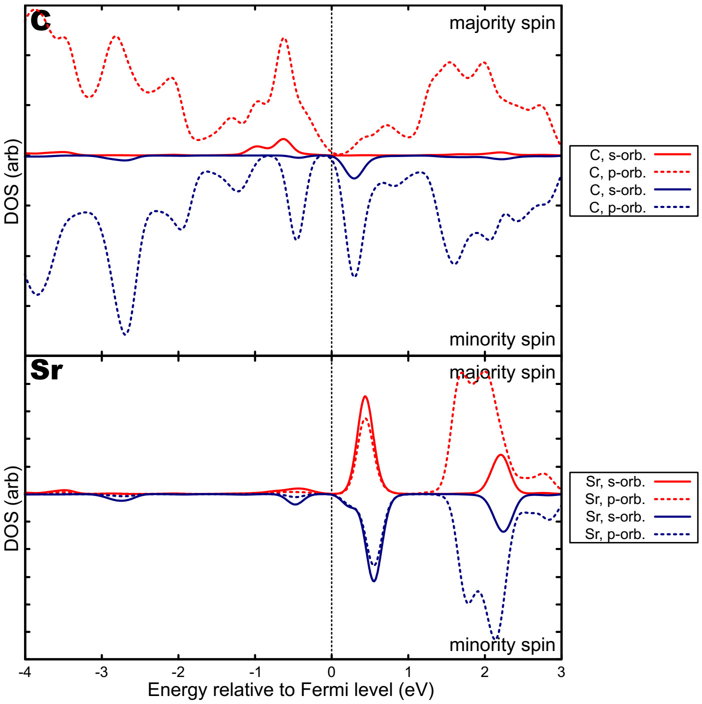

(b) projected DOS (pDOS) for Sr-graphene

Fig. H20 Electronic properties and orbital hybridization for Sr-graphene at 1200 eV energy cutoff. Red indicates majority spin (spin up) states and blue indicates minority spin (spin down) states. Supplementary information for electronic properties at Fig. H21†.

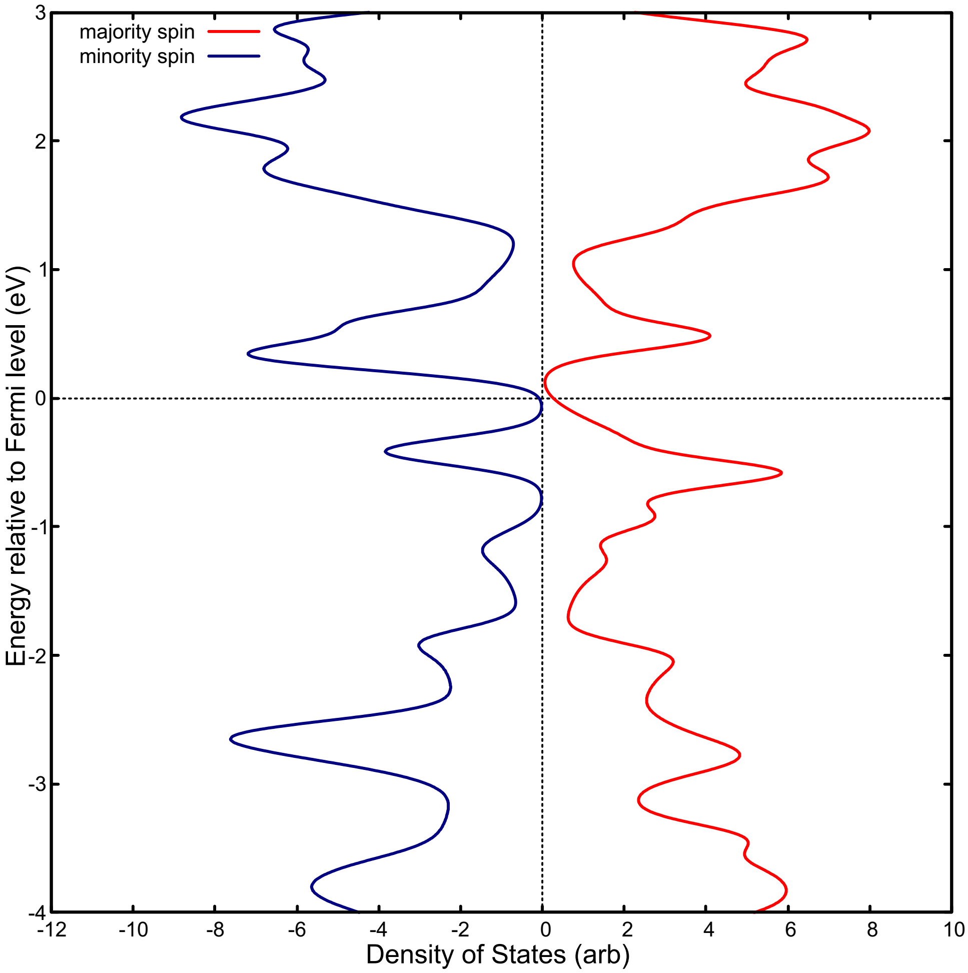

(a) Density of states (DOS)

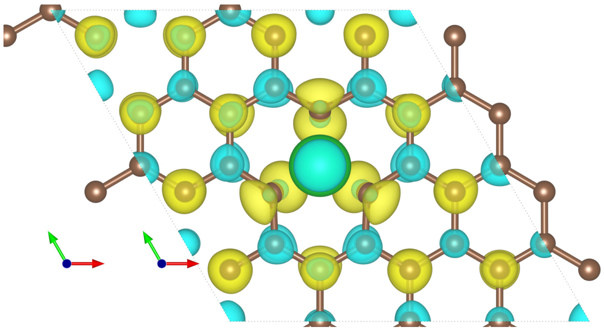

(b) majority spin VBM and CBM partial electron density

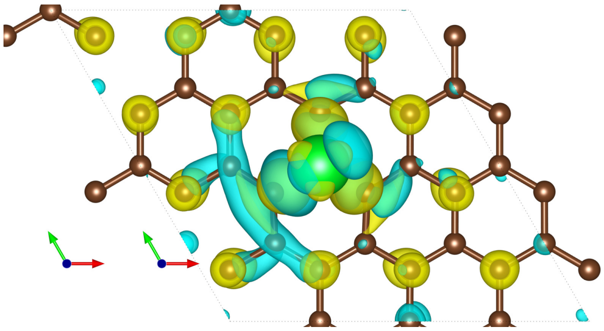

(c) minority spin VBM and CBM partial electron density

Fig. H21 DOS and VBM and CBM partial charge densities for Sr-graphene at 1200 eV energy cutoff. For the DOS, red indicates majority spin (spin-up) states and blue indicates minority spin (spin down) states. For the VBM and CBM partial charge densities, yellow indicates regions contributing to the VBM, while cyan indicates regions contributing to the CBM. Brown atoms are carbon atoms while the differently colored atom denotes the alkaline earth dopant. Isosurface level for VBM and CBM partial charge densities = $0.0015e^-/bohr^3$

Notes and references

1 F. Lopez-Urias, M. Terrones and H. Terrones, *Carbon*, 2015, **84**, 317–326.

2 A. C. F. Serraon, A. A. B. Padama, J. A. D. del Rosario and J. D. Ocon, *ECS Transactions*, 2017, **77**, 629–636.

3 H. Luo, L. Zhang, S. Xu, M. Shi, W. Wu and K. Zhang, *Applied Surface Science*, 2020, 147542.

4 S. Ullah, A. Hussain, W. Syed, M. A. Saqlain, I. Ahmad, O. Leenaerts and A. Karim, *RSC Advances*, 2015, **5**, 55762–55773.

5 C. Klain, S. Linde, R. Shikler and G. Sarusi, *Carbon*, 2020, **157**, 255–261.

6 K. C. Kwon, K. S. Choi and S. Y. Kim, *Advanced Functional Materials*, 2012, **22**, 4724–4731.

7 R. Martínez-Orozco, H. Rosu, S.-W. Lee and V. Rodríguez-González, *Journal of Hazardous Materials*, 2013, **263**, 52–60.

8 E. Rut'kov, E. Afanas'eva and N. Gall, *Diamond and Related Materials*, 2020, **101**, 107576.

9 T. Yoon, Q. Wu, D.-J. Yun, S. H. Kim and Y. J. Song, *Scientific Reports*, 2020, **10**, 9870.

10 R. Yan, Q. Zhang, W. Li, I. Calizo, T. Shen, C. A. Richter, A. R. Hight-Walker, X. Liang, A. Seabaugh, D. Jena, H. Grace Xing, D. J. Gundlach and N. V. Nguyen, *Applied Physics Letters*, 2012, **101**, 022105.

11 T. Takahashi, H. Tokailin and T. Sagawa, *Physical Review B*, 1985, **32**, 8317–8324.

12 Y. Shi, K. K. Kim, A. Reina, M. Hofmann, L.-J. Li and J. Kong, *ACS Nano*, 2010, **4**, 2689–2694.

13 C. Oshima and A. Nagashima, *Journal of Physics: Condensed Matter*, 1997, **9**, 1–20.

14 J.-Y. Syu, Y.-M. Chen, K.-X. Xu, S.-M. He, W.-C. Hung, C.-L. Chang and C.-Y. Su, *RSC Advances*, 2016, **6**, 32746–32756.

15 B. Shan and K. Cho, *Physical Review Letters*, 2005, **94**, 236602.

16 T. Hu and I. C. Gerber, *The Journal of Physical Chemistry C*, 2013, **117**, 2411–2420.

17 K. Nishidate, N. Yoshimoto, P. Chantngarm, H. Saito and M. Hasegawa, *Molecular Physics*, 2016, **114**, 2993–2998.

18 G. Bae, H. Jung, N. Park, J. Park, S. Hong and W. Park, *Applied Physics Letters*, 2012, **100**, 183102.

19 W. Hu, Z. Li and J. Yang, *The Journal of Chemical Physics*, 2013, **138**, 124706.

20 N. Yang, D. Yang, L. Chen, D. Liu, M. Cai and X. Fan, *Nanoscale Research Letters*, 2017, **12**, 642.

21 G. Giovannetti, P. A. Khomyakov, G. Brocks, V. M. Karpan, J. van den Brink and P. J. Kelly, *Physical Review Letters*, 2008, **101**, 026803.

22 S.-M. Choi, S.-H. Jhi and Y.-W. Son, *Physical Review B*, 2010, **81**, 081407.

23 O. Leenaerts, B. Partoens, F. M. Peeters, A. Volodin and C. Van Haesendonck, *Journal of Physics: Condensed Matter*, 2017, **29**, 035003.

24 H. Hibino, H. Kageshima, M. Kotsugi, F. Maeda, F.-Z. Guo and Y. Watanabe, *Physical Review B*, 2009, **79**, 125437.

25 A. Savin, R. Nesper, S. Wengert and T. F. Fässler, *Angewandte Chemie International Edition in English*, 1997, **36**, 1808–1832.
